# Genomic and Antigenic Differences Between Monkeypox Virus and Vaccinia Vaccines: Insights and Implications for Vaccinology

**DOI:** 10.3390/ijms26041428

**Published:** 2025-02-08

**Authors:** Jane Shen-Gunther, Hong Cai, Yufeng Wang

**Affiliations:** 1 Gynecologic Oncology & Molecular Medicine, Department of Molecular Medicine, University of Texas Health Science Center at San Antonio, San Antonio, TX 78229, USA; 2 Department of Molecular Microbiology and Immunology, University of Texas at San Antonio, San Antonio, TX 78249, USA; hong.cai@utsa.edu; 3 South Texas Center for Emerging Infectious Diseases, University of Texas at San Antonio, San Antonio, TX 78249, USA

**Keywords:** bioinformatics, disease outbreaks, monkeypox, monkeypox virus, phylogeny, poxvirus, taxonomic classification, vaccinia virus, virus database

## Abstract

Amid the current multi-country mpox outbreak, analyzing monkeypox virus (MPXV) and vaccinia virus (VACV) genomes is vital for understanding evolutionary processes that may impact vaccine efficacy and design. This study aimed to elucidate the phylogenetic relationships and structural features of viral antigens, which are crucial for developing effective vaccines. By aligning 1903 MPXV genomes from the NCBI Virus repository (released between 2022 and 2024), an increase in phylogenetic diversity was observed compared to previous studies. These genomes were grouped into Clade I (25 genomes) and Clade IIB (1898 genomes), with a new Clade I sub-lineage emerging from samples collected in Sud-Kivu province, Democratic Republic of the Congo (DRC). Comparing six key MPXV neutralization determinants (A29, A35, B6, E8, H3, and M1) of a novel 2024 Clade I MPXV isolate to those of the 1996 Zaire isolate revealed remarkable sequence conservation despite spanning 28 years. Homology-based modeling of the Clade I MPXV antigens (A29, A35, E8, H3, and M1) showed high-match identities (84% to 99%) with VACV templates (current mpox vaccine), with several amino acid variants near potential antibody binding sites. Phylogenomic analysis, combined with structural modeling and variant profiling, has yielded valuable insights into the virus and vaccine, guiding vaccine design and functional studies.

## 1. Introduction

On 14 August 2024, the World Health Organization (WHO) declared mpox a public health emergency of international concern (PHEIC) due to a surge in cases and fatalities, as well as its multi-country spread across the African continent [1]. Mpox, an infectious disease caused by the monkeypox virus (MPXV) of the *Orthopoxvirus* genus, has been endemic to Central and West Africa for over five decades [2,3,4,5,6]. The unprecedented global outbreak of mpox, declared a PHEIC on 23 July 2022, and its resurgence in 2024, marked a significant shift in transmission dynamics and the affected population, expanding from primarily gay/bisexual men to include women, children, and infants [2,7]. Consequently, concerns about viral mutagenesis and evolution have been raised. MPXV mutations have been linked to the activity of the human APOBEC3 (apolipoprotein B mRNA-editing catalytic polypeptide-like 3) enzymes, which induce cytidine deamination during viral replication, leading to mutations in the viral genome [8,9,10,11]. These mutations may inadvertently provide a fitness advantage during infection by altering viral proteins to facilitate transmission and immune evasion [9]. Therefore, the interaction between APOBEC3 and MPXV may be contributing to viral diversity and evolutionary fitness. This is indicated by the emergence of a new sub-lineage within Clade I of MPXV in 2023 and the upsurge in cases, particularly in the Democratic Republic of the Congo (DRC) (formerly Zaire) [12,13,14]. The latest WHO Situation Report from October 2024 disclosed alarming year-to-date figures for the DRC, with 31,350 suspected cases and 992 deaths among them [15].

Today’s mpox prophylactic vaccines are sourced from the live attenuated vaccinia virus (VACV) originally developed for smallpox [2]. Currently, there are only three licensed vaccines available globally, each subject to country-specific regulations and restrictions [2]. The most widely available vaccine is the modified vaccinia Ankara–Bavarian Nordic (MVA-BN), developed in Denmark and patented in 2007 for use in adults (18 years and older) [2,16,17]. In 2019, the United States Food and Drug Administration (FDA) approved MVA-BN for mpox prophylaxis in adults and granted emergency use authorization (EUA) in 2022 for individuals under 18 years of age [18]. On October 8, 2024, the WHO granted prequalification to the MVA-BN vaccine for individuals aged 12 to 17 [15]. In Japan, the LC16m8 vaccine was initially licensed in 1975 for smallpox prophylaxis and was expanded in 2022 to include mpox prophylaxis without age restrictions [2,19,20,21]. The third vaccine, ACAM2000, was developed by the United States for biological defense and approved by the FDA for smallpox prophylaxis [22]. It is available for use against mpox only under an expanded access investigational new drug (EA IND) protocol [23]. These vaccines originated from distinct lineages of VACV that have been propagated over the last century, resulting in differences in their genomes, antigens, and safety profiles [24,25,26,27,28]. Various laboratory and propagation techniques, including the use of different host species, cell cultures, and passage numbers, have modified the VACV genomes [24]. In recent years, genome sequencing has decoded several ancestral poxviruses and VACV genomes [21,29,30,31,32]. These advancements now facilitate lineage tracing and the comparative analysis of genomes and antigens.

**Figure 1:**
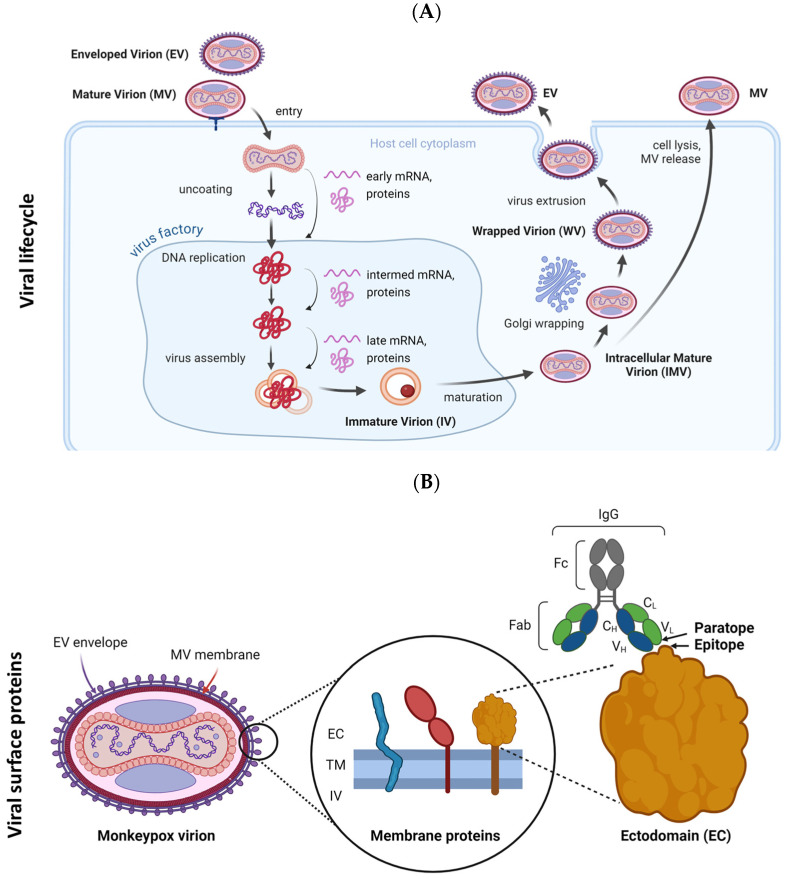
Monkeypox viral lifecycle and antigenic surface proteins. (**A**) The viral lifecycle comprises several distinct stages, namely attachment to the host cell, entry, uncoating, protein expression, DNA replication, assembly, maturation, and release. Pox virions exist as either infectious mature virions (MVs) or the less abundant enveloped virions (EVs) [38,39]. (**B**) The MV features an outer membrane composed of lipids and viral proteins, while the EV is encased in an additional lipid bilayer with unique virus-encoded proteins. The proteins in the EV envelope (A35, B6) and MV membrane (A29, E8, H3, M1) are immunogenic and can be neutralized by cross-reactive antibodies derived from vaccination with attenuated vaccinia virus [37,38]. EC, ectodomain; IV, intraviral domain; TM, transmembrane domain (figure created with BioRender.com).

The MVA vaccines have demonstrated an effectiveness of 66 to 89% with two doses, though vaccine breakthroughs and failures have been reported [33,34,35]. Factors such as host immunodeficiency, viral immune escape, and emerging variants are recognized contributors to these events [33,34]. For the vaccines to be efficacious, their neutralization determinants must be target-specific and induce a cross-neutralizing redundant antibody response [36]. Among *Orthopoxviruses*, six key neutralization determinants have been identified, which are expressed at distinct stages of the viral lifecycle (Figure 1) [36,37]. The emergence of the new sub-lineage within Clade I of MPXV has raised concerns about nonsynonymous mutations causing structural changes in virion epitopes. These changes could lead to ineffective binding by host cross-neutralizing antibodies, potentially resulting in vaccine failures and breakthroughs [25,26,33,34,35].

This study aimed to investigate the unique evolutionary paths of MPXV and VACV genomes, as well as to characterize and compare their present neutralization determinants. Focusing on the highly virulent MPXV Clade I genome, we analyzed the encoded protein sequences of six key neutralization determinants shared between MPXV and VACV virions and performed 3D structural modeling for variant profiling and visualization [36,37]. This preliminary in silico study provides valuable insights into the virus and vaccine, paving the way for future experimental research.

## 2. Results

### 2.1. MPXV Genomes from 2022 to 2024 Clustered with Clades I and IIB, with a New Clade I Sub-Lineage Emerging from Sud-Kivu, DRC

**Figure 2:**
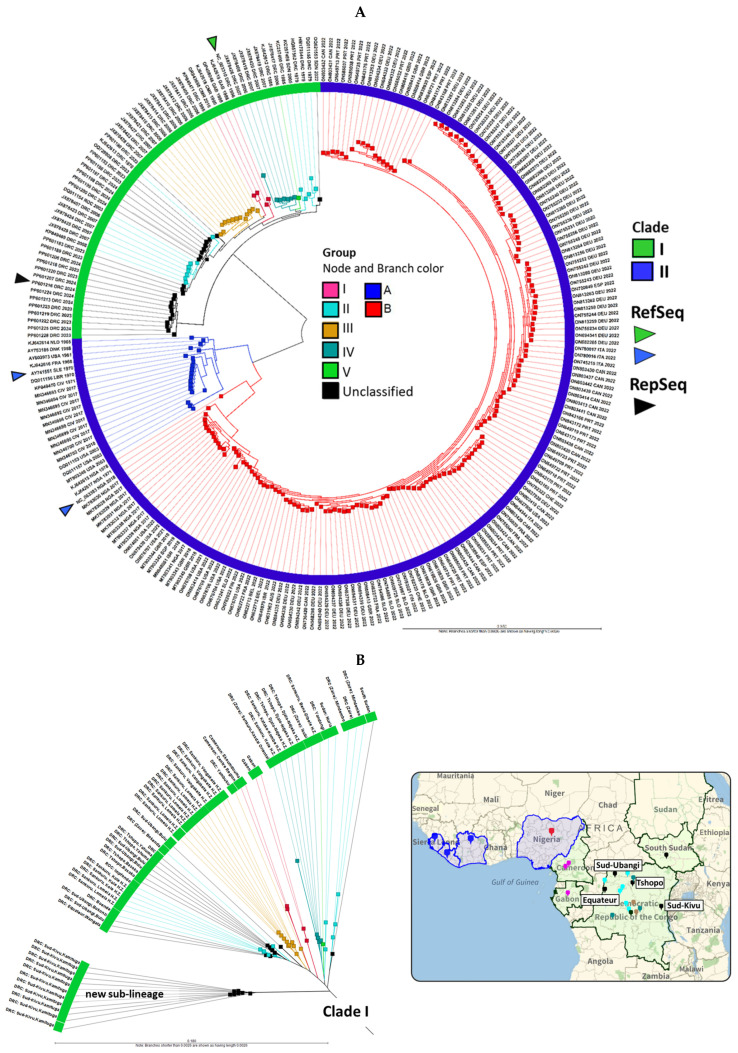
Phylograms of MPXV genomes for the visualization of evolutionary and geo-temporal relationships. (**A**) Phylogram of complete MPXV genomes (*n* = 243) by clades. Clades I and II originated from Central and West Africa, respectively, with divergent branches. The sub-lineages (groups) reveal the relatedness of its member samples. Three attributes of each genome (sequence AN, 3-letter country code, and collection year) are displayed as the outermost ring. Arrowheads indicate the MPXV reference sequences (RefSeq) for Clades I and II, as well as the representative sequence (RepSeq) (PP601216) from the novel sub-lineage. (**B**) The radial phylogram of Clade I MPXV genomes, annotated with country and city/province attributes, reveals phylogeography. The map links the colors of the clades and groups to respective countries and cities/provinces (drop pins). The ANI NJ unrooted trees were constructed from the pairwise comparison table.

The whole-genome alignment (WGA) of 1903 MPXV genomes was performed in batches of 200 alongside reference genomes from Clades I (NC_003310), IIA (AY741551), and IIB (NC_063383). Phylogenomic analysis showed 25 genomes aligned with Clade I and 1898 with Clade IIB. The 25 Clade I genomes were added to our initial MPXV database (218 genomes) for further analysis and displayed as a neighbor joining (NJ) phylogram with 243 MPXV genomes in Figure 2A. MPXV Clades I and II, originating from Central and West Africa, respectively, have distinct branches, comprising groups I-V and A-B [5,6,40]. The outermost ring of the phylogram shows three attributes for each genome, namely the sequence AN, three-letter country code, and year, enhancing the visualization of geo-temporal data. Of the 25 new Clade I genomes, all hosts were human except for AN OR943698, which came from a captive chimpanzee during a 2016 outbreak at the Mefou Primate Sanctuary in Cameroon and was clustered with Group I [41]. Another sample, AN OP498046, collected from a 9-month-old girl in Gabon in 1988 but sequenced in 2022, was also clustered with Group I [42]. Twelve samples (AN OQ621553, OQ729808, and PP601183-PP601206), unclassified by the authors, closely aligned with Group II [12]. Eleven additional unclassified genomes (AN PP601207-PP601228) by the same authors collected in Sud-Kivu formed a new Clade I sub-lineage (Figure 2A) [12].

The MPXV Clade I genomes from the complete set of 243 genomes were extracted and displayed as a radial phylogram by group, country, and city/province (if available) in Figure 2B. The map correlates the phylogenetic and geospatial relationship. The metadata for the 243 genomes is provided in Supplementary Table S1. The Kamituga Health Zone in Sud-Kivu province is easily identifiable on the eastern border of the DRC near Tanzania, where the new sub-lineage had emerged. In contrast, genomes from Sud-Ubangi, Tshopo, and Equateur, DRC, as well as South Sudan, aligned with the pre-existing Group II genomes. Furthermore, the limited number of genomes (*n* = 11) from Sud-Kivu in the NCBI Virus does not reflect the disease’s extent and virulence, with 31,350 suspected mpox cases reported in the DRC between January and October 2024 [15]. The distinct novel sub-lineage has raised concerns about APOBEC3-mediated intra-host mutagenesis and viral microevolution [12]. Consequently, we selected the most ancestral genome from Sud-Kivu (PP601216) as the representative genome, along with the Clade I RefSeq (NC_003310), for downstream comparative analysis and antigen modeling. Phylograms from Figure 2, displayed at page width, are shown in Supplementary Figure S1. The 1898 genomes released between 2022 and 2024 that grouped with Clade IIB are displayed as phylograms in Supplementary Figure S2, with metadata provided in Supplementary Table S2. The maximum likelihood (ML) trees for MPXV Clade I genomes are shown in Supplementary Figure S3 as five smaller trees, reflecting the partitioned nature of the analysis. The overall lineage structure is consistent between the NJ and ML trees. The separation of the two sub-lineages for Group II observed in both NJ and ML trees compared to the original Bayesian tree by Berthet et al. [5] is likely due to differences in tree construction methods. Importantly, this did not affect the alignment and clustering of the novel Clade I genomes.

### 2.2. VACV Genomes Feature a Conserved Central Region and Variable Terminal Regions, Which Exhibit Rearrangements, Truncations, and Deletions in Later Generations of the Vaccine

Genome alignment and a comparative analysis of nine representative VACV, two cowpox virus (CPXV), and one horsepox virus (HPXV) genomes revealed a conserved central region, variable terminal synteny blocks, and shortened genome lengths (Supplementary Figure S4). The longest VACV genome was VACV-ACAM2000 (199,234 bp), while the shortest was VACV MVA-BN (165,041 bp). The VACV RefSeq genome length (194,711 bp) was 17,922–29,788 bp shorter than the RefSeqs for HPXV (212,633 bp) and CPXV (224,499 bp). The distal synteny blocks beyond the 200,000 bp position, which are homologous to those in the CPXV, HPXV, and VACV RefSeqs, were truncated, rearranged, or deleted in later generations of VACV. Remarkably, the genes for the six neutralization determinants were identified in the genomes of two current vaccines, VACV MVA-BN and LC16m8. However, the B5 protein of LC16m8 is known to be truncated and induces low levels of anti-B5 IgG in mouse models [43].

**Figure 3:**
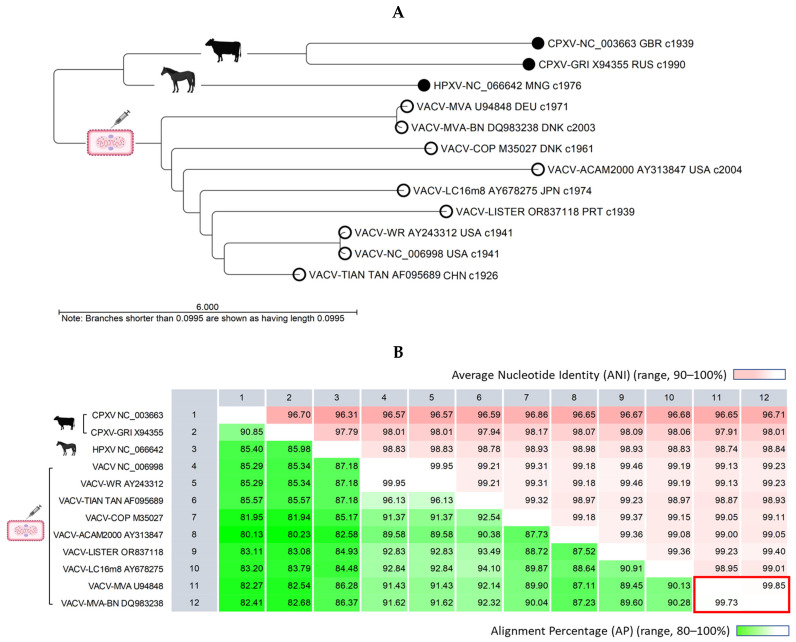
Comparative analysis of representative vaccinia, cowpox, and horsepox viral genomes. (**A**) The phylogenetic tree depicts the genetic distances between VACV (white circle) and ancestral zoonotic pox genomes (black circle), indicating a closer relationship to HPXV compared to CPXV (unrooted AP NJ tree). Within the VACV clade, genetic variations are apparent, with divergent branches. The labels display four attributes of each genome (virus strain, sequence AN, 3-letter country code, and approximate collection year). (**B**) The pairwise comparison table reveals alignment statistics. The VACV RefSeq (NC_006998) exhibited greater similarity to HPXV compared to CPXV, with alignment percentages of 87% vs. 85%, respectively. The alignment similarity between VACV genomes exceeded 87%, and within these aligned regions, the nucleotide identity surpassed 98%. The current Bavarian Nordic vaccine (VACV-MVA-BN) compared to its ancestral modified vaccinia Ankara genome was highly conserved, with over 99% AP and ANI (red rectangle). AP, alignment percentage; ANI, average nucleotide identity; CPXV, cowpox virus; HPXV, horsepox virus; NJ, neighbor-joining; VACV, vaccinia virus.

The unrooted API NJ phylogenetic tree illustrates genetic distances, showing VACV is closer to HPXV than CPXV (Figure 3A). Pairwise genome comparisons revealed alignment similarities of 82–87% between VACV and HPXV and 80–85% between VACV and CPXV (Figure 3B). Within the VACV clade, the distinct divergent branches indicated genetic differences. A pairwise comparison (PWC) showed alignment similarity exceeding 87% between VACV genomes, with nucleotide identity surpassing 98% within aligned regions (Figure 3B).

### 2.3. MPXV Clade I Genomes and Current VACV Vaccines Differ in Genome Lengths and Terminal Regions but Share over 85% Alignment Similarity

**Figure 4:**
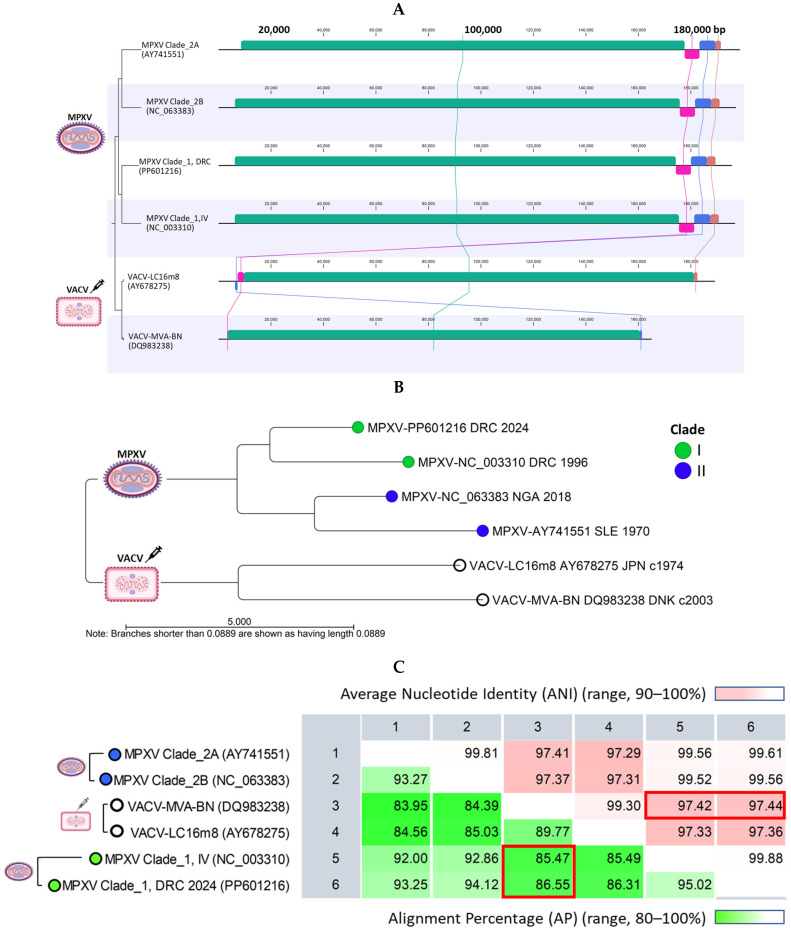
Comparative analysis of representative monkeypox and vaccinia viral genomes. (**A**) WGA revealed a highly conserved central region for representative MPXV Clades I and II genomes (green synteny blocks). Significant variability was observed at the distal ends, before 6500 bp and beyond 190,000 bp. In comparison, the VACV-MVA-BN and VACV-LC16m8 are shortened, with synteny blocks at the distal ends being truncated, deleted, or rearranged (pink, blue, and brown synteny blocks). (**B**) The unrooted AP NJ tree illustrates the genetic distances between representative MPXV and VACV genomes (white circle), forming two distinct clades. Genetic differences are evident between the MPXV Clades and current vaccines (VACV-MVA-BN and VACV-LC16m8). (**C**) Pairwise comparison between the MPXV Clade I and VACV MVA-BN genomes quantified alignment similarity at over 85–86% and ANI at over 97% (red rectangles). The statistics for VACV-LC16m8 were nearly identical to those for VACV MVA-BN. AP, alignment percentage; ANI, average nucleotide identity; NJ, neighbor joining; VACV, vaccinia virus.

Genome alignment revealed a highly conserved central region in MPXV Clades I and II, spanning approximately from 6500 to 175,000 bp (Figure 4A). Significant variability was observed at the genome’s distal ends. In contrast, the VACV genomes (MVA-BN and LC16m8) are shorter, with genes at the distal ends curtailed, rearranged, or deleted, as indicated by the pink, blue, and brown synteny blocks in Figure 4A. These deletions align with the characteristics of attenuated, replication-proficient (LC16m8), and replication-deficient (MVA-BN) vaccines, which have undergone extensive serial passages. A phylogenetic tree was constructed from the pairwise comparison of representative MPXV and VACV (MVA-BA and LC16m8) genomes. Figure 4B shows the two distinct clades formed by MPXV and VACV genomes, with genetic differences evident between individual genomes (divergent branches). The representative genome from Sid Kivu (PP601216), closely aligned with the Clade I RefSeq, shows 95% alignment similarity and 99.88% ANI (Figure 4C). Pairwise comparisons between MPXV Clade I and the genomes of VACV MVA-BN and VACV-LC16m8 showed alignment similarities exceeding 85–86% and ANI over 97% (Figure 4C).

### 2.4. Variations in Sequence and Structure Between Homologous MPXV and VACV Antigens May Impact Epitope/Paratope Binding

**Figure 5:**
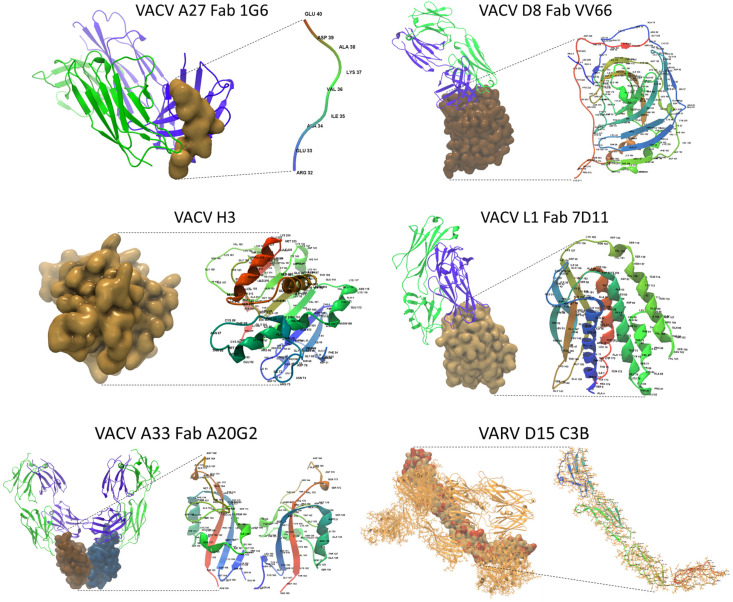
Vaccinia and variola virus template structures. A collage of five vaccinia and one variola virus antigenic ectodomains used as templates for MPXV 3D modeling. Each ectodomain is shown as a surface model (**left**) and an AA-labeled backbone model (**right**). The backbone model uses a rainbow color scale which transitions from blue to green, yellow, and red, corresponding to the residue number The Fab regions of monoclonal antibodies are depicted as backbone models, with VH (blue) or VL (green) binding to the surface ectodomains. VACV A33 is shown as a homodimer. For VACV H3 and VARV D15, only an ectodomain and an ectodomain–C3b complex, respectively, were available in the PDB. (PDB Entry IDs listed in Data Availability Statement).

Table 1 presents a summary of six crystallographic structures identified for the surface antigenic proteins of MPXV Clade I (NC 003310) by the “Find and Model Structure” tool. The MV proteins (A29L, E8L, H3L, and M1R) and EV proteins (A35R and B6R) showed highly significant matches (E-value range, 3.1 × 10^−20^ to 1.7 × 10^−158^) and match identities (range, 84 to 99%) with homologous proteins on VACV, except for B6R. The MPXV B6R had a satisfactory match (E-value, 1.0 × 10^−11^; match identity, 30%) with the structure of the smallpox inhibitor of complement (SPICE). The percentage coverage, indicating the portion of the full-length AA sequence covered by the ectodomain sequence encoding the crystallographic structures, ranged from 38.7% to 75.4%. Remarkably, the structural modeling results for the representative MPXV genome from Sud-Kivu (PP601216) matched those of the 1996 Clade I RefSeq (NC_003310) (Supplementary Tables S3 and S4), highlighting well-preserved neutralization determinants. This was consistent with the near perfect match between the six full-length antigenic sequences of Sud-Kivu (PP601216) and RefSeq (NC_003310). Only one AA differed in the A35 sequence. For MPXV antigen modeling, top homologous structures, preferably complexed with monoclonal antibodies, were selected. This approach visualized AA differences between the constructed model and the template, along with epitope–paratope binding sites. The vaccinia virus surface antigens, shown as a collage of ectodomains in Figure 5, served as templates for MPXV 3D modeling. The VACV proteins homologous to MPXV included MV proteins (A27, D8, H3, L1) and the EV protein (A33). For MPXV B6, the top homologous structure was the variola virus protein (VARV D15). Each ectodomain is shown as a surface and an AA-labeled backbone model in Figure 5. The Fab regions of monoclonal antibodies are shown in complex with the ectodomains. The epitope/paratope binding sites may help identify potential binding discrepancies resulting from structural variations in the homologous MPXV antigens. The VARV D15 is shown as an ectodomain-complement C3b complex.

**Figure 6:**
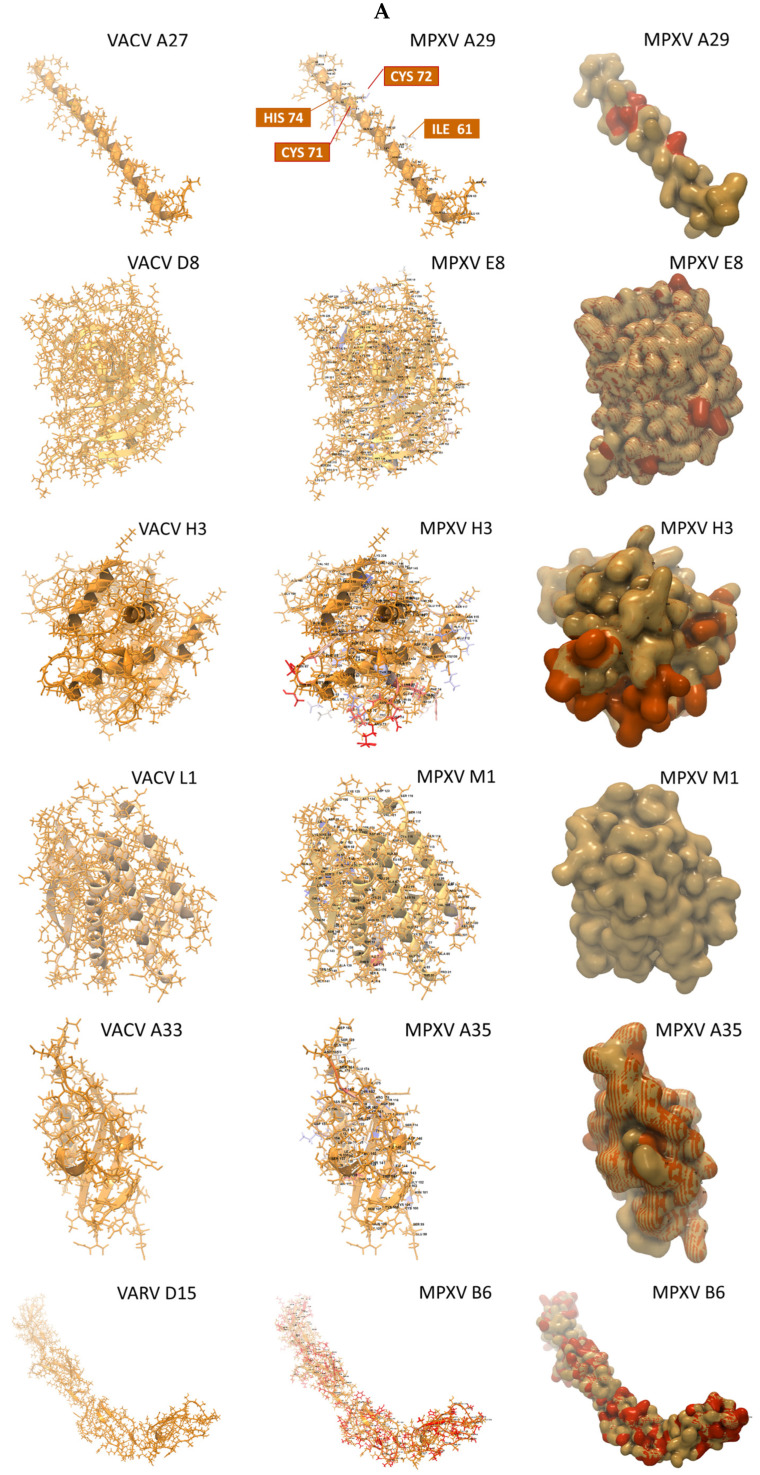

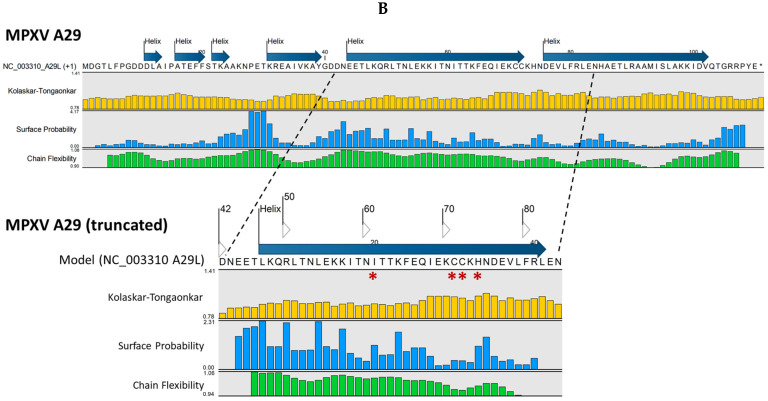
Monkeypox virus surface antigen models with protein analysis. (**A**) Collage of six MPXV antigenic ectodomains generated by homology-based 3D modeling. The top ranking VACV and VARV ectodomain templates are shown as stick and backbone models in monochrome gold (left column). The superimposed MPXV backbone models highlight nonsynonymous AA in non-gold colors (middle column). The MPXV surface models reveal variant AA (orange) superimposed over the gold template (right column). For MPXV B6R, the highest-ranking template was VARV D15, a structure of smallpox inhibitor of complement (SPICE). All models are shown as monomers. (**B**) Protein analyses are displayed as tracks, showcasing the AA sequence, structural features, antigenicity, and physicochemical properties [46,47,48,49]. The representative complete sequence of MPXV A29, along with its predicted secondary structure (strand or helix), is displayed over the sequence. The Kolaskar–Tongaonkar, surface probability, and chain flexibility tracks (colored) indicate predicted antigenic regions based on (1) hydrophilicity, surface accessibility, and flexibility; (2) surface probability; and (3) backbone chain flexibility, respectively [46,47,48,49]. The zoomed-in truncated segment with the predicted antigenic potential of four variant AA (*) (ILE, CYS, CYS, HIS) corresponds to the surface model and variants depicted in panel (**A**). The asterisk (*) indicates a stop codon.

In Figure 6A, the VACV ectodomain templates are juxtaposed with the homologous MPXV 3D models. The VACV templates are depicted as stick and backbone models in monochrome gold. The superimposed MPXV stick and backbone models highlight AA variants (labeled) in non-gold colors (page-width models are shown in Supplementary Figure S5). The MPXV surface models superimposed over the gold templates reveal AA variants in orange. For example, the MPXV A29 models display four labeled AA variants (ILE, CYS, CYS, HIS) on the backbone model and orange molecules on the surface model.

The AA alignment for the six MPXV antigen models and templates are shown in Supplementary Figure S6. A comparison of full-length antigen sequences between MPXV and the two current VACV vaccines was also conducted. The alignment and pairwise comparison showed that the percent identities for A29L, A35L, E8L, and H3L between MPXV (NC003310) and the VACV vaccines (MCV-BN and LC16m8) xwere notably high, ranging from 93% to 95%, with M1 being the highest at 98.8% (Supplementary Figure S7). In contrast, the percent identity for B6 between MPXV (NC_003310) and the VACV vaccines (MCV-BN and LC16m8) showed a significant difference, at 96% and 41%, respectively. This finding is consistent with the known truncated B6 in LC16m8 [43].

Protein analysis of the six MPXV antigenic proteins was also conducted. The complete AA sequence of each protein was entered into the “Predict Secondary Structure” tool, and the resulting predicted structure (strand or helix) was displayed above the sequence. Additional “protein information” tracks were selected to view the predicted antigenic potential of an AA or segment (Supplementary Figure S8). A representation of protein analysis using MPXV A29 as an example is shown in Figure 6B. The Kolaskar–Tongaonkar, surface probability, and chain flexibility tracks are predictions of antigenic regions based on (1) hydrophilicity, surface accessibility, and flexibility; (2) surface probability; and (3) backbone chain flexibility, respectively [46,47,48,49]. Generally, increased surface accessibility and chain flexibility correlate with antigenicity. A surface residue is usually one having more than 20 Å (2.0 nm) of water-accessible surface area [48]. The surface probability (S) at a specific sequence position (*n*) for a random hexapeptide sequence is set to 1.0. Probabilities > 1.0 suggest a higher likelihood of the residue being on the surface [48]. For protein chain flexibility, B-Factors (temperature factors) are indicative of atomic displacement or flexibility within the protein structure. Higher B-factors (>1.0) suggest greater flexibility [49]. The Kolaskar–Tongaonkar composite measure defines potential antigenic residues as having an average antigenic propensity (Ap) value ≥ 1.0 for seven consecutive residues [47]. In Figure 6B, the MPXV A29 full-length AA sequence is shown. The truncated A29 segment with the predicted antigenic potential of four variant AAs (ILE, CYS, CYS, HIS) corresponds to the surface model and variants in Figure 6A.

## 3. Discussion

In this study, we investigated the evolution of MPXV and VACV genomes and compared homologous antigens relevant to current vaccine efficacy and design. Leveraging our expanded MPXV database, we efficiently performed WGA, PWC, and phylogenetic analysis through automated workflows. The overall lineage structure of the NJ trees was validated by the more accurate yet computationally intensive ML method. Crucial insights into the geo-temporal relationships of the phylogenetic trees, vital for outbreak tracking, were derived from the database metadata. The alignment of 1903 newly deposited MPXV genomes from 30 countries revealed greater phylogenetic diversity compared to our previous study [6]. We identified 25 genomes clustering with Clade I and 1898 with Clade IIB. Eleven genomes from Sud-Kivu, DRC, formed a new sub-lineage within Clade I, presumably due to human APOBEC3 [8,9,10,11,50]. Remarkably, the six neutralization determinants encoded by the 2024 novel sub-lineage genome (PP601216) remained conserved compared to the Clade I RefSeq (NC_003310) from 1996. These viral surface proteins, essential for cell attachment and entry, are typically conserved due to their critical function, structural necessity, and strong evolutionary pressures to maintain viral infectivity and transmissibility. Thus, the six conserved neutralization determinants are ideal candidates as antigens in future vaccine design. Nevertheless, continuous MPXV genomic surveillance is essential for detecting changes in viral evolution and dynamics within individuals or populations. Mutations that provide a functional selective advantage could eventually dominate, potentially leading to resistance against current vaccines.

A comparative analysis of three generations of VACV genomes with HPXV and CPXV reference genomes has revealed notable evolutionary insights. It was found that VACV is distinct but phylogenetically closer to HPXV than to CPXV, consistent with prior studies [51,52]. However, the limited availability of HPXV and CPXV genomes from primary hosts, along with the age of the samples, may introduce bias into the findings. In particular, the RefSeq for HPXV is the only field isolate (MNR-76) available in NCBI Virus, collected during a horsepox outbreak in 1976 in Khentii province of northeastern Mongolia [29]. Another HPXV genome (VK05) was sequenced from historical vaccination kits stored at the Mütter Museum in Philadelphia, dating back to the American Civil War era (circa 1859–1873) [30]. The CPXV RefSeq (Brighton Red strain) was isolated in 1937 from a milker in Brighton, England, while the CPXV GRI-90 strain was isolated in 1990 from a four-year-old Russian child infected by a mole (mammal) [53,54]. Regarding the VACV genomes, we noted variations in sequence length, as well as deletions and rearrangements of synteny blocks at the terminal regions. VACV MVA-BN, a third-generation vaccine, exhibited the shortest genome with a truncated distal synteny block but still retained the six neutralization determinants. These genomic modifications underscore the profound effect of serial passage, a process pioneered by Louis Pasteur, on genome reductive evolution [25,27,28].

A comparison of the structural models of the six neutralization determinants from MPXV RefSeq (NC_003310) with five VACV crystallographic templates and one VARV template revealed a few but significant amino acid differences near potential epitope/paratope binding sites. Quantitatively, four of the MPXV models exhibited approximately 90% identity to the VACV template. MPXV M1 had an almost perfect match at 99%, whereas the top homologous B6 template (variola SPICE) was less than ideal, with only 29.6% identity. Due to the absence of an experimental model for VACV B5, Riccardo et al. utilized AlphaFold2 to predict an in silico model [37]. Given that MPXV B6 and VACV MVA-BN B5 share a 96% AA sequence identity based on our alignment results, their structures are presumed to be similar. However, only an experimental model can validate the differences in epitopes. In contrast, our alignment showed a 41% AA sequence identity between MPXV B6 and VACV LC16m8 B5, which is consistent with the truncated B5 protein of LC16m8 [43]. Considering the essential functions of these viral surface proteins in cell attachment and entry, it is probable that they have evolved to adapt to host-specific cell membrane proteins and structures, leading to the observed differences. Although imperfect, the long-standing vaccinia vaccines, initially created for smallpox, still play a crucial role in safeguarding humanity against mpox today. Computationally, we have showcased a streamlined method of in silico modeling for investigating structural differences. These findings can guide experimental testing and validate their significance through antigen–antibody (Ag-Ab) binding assays, which are essential for vaccine design.

The strength of this study lies in the extensive number of MPXV genomes analyzed, enabling the identification and analysis of novel Clade I genomes. Additionally, the complete coding sequences were utilized for the structural modeling and comparative analysis of antigenic proteins. Furthermore, the streamlined step-by-step process we have outlined for investigating newly sequenced or publicly deposited samples will facilitate scientific discovery and enhance the surveillance and monitoring efforts of novel variants.

We acknowledge that our study has limitations, particularly the scarcity of fully sequenced novel Clade I genomes available in the NCBI repository. In fact, all these novel genomes were deposited by Vakaniaki et al. [12]. Although the NCBI Virus Database is highly regarded for its comprehensiveness, it may not include all emerging viruses, especially those that are newly discovered or understudied. This limitation can impede access to critical information about novel viral genotypes or variants, especially during outbreaks [55]. The database may also lag in updating the latest sequences, leading to data gaps for research needs [55]. Consequently, small sample sizes may fail to capture the full extent of viral diversity within the population, potentially skewing results. The underrepresentation in the database might stem from a lack of resources, research funding, public health interest, or the availability of sequencing technologies [56]. In fact, these and other challenges were highlighted in a recent feature article, “Congo’s Mpox Crisis”, in *Science* [57]. Hence, public health funding and support are essential for ongoing disease surveillance, sequencing, and data sharing to combat (re)emerging virus outbreaks.

Another limitation is the lack of experimental crystal structures for the six MPXV surface proteins. Crystal structures are crucial for validating our homology-based models for downstream applications such as antigen selection or vaccine design. Furthermore, the quality of structures in the PDB vary widely. Entries from X-ray crystallography may have errors due to low resolution and model bias [58,59,60]. Additionally, the absence of standardized validation criteria across entries has resulted in inconsistent or incomplete data, affecting protein modeling outcomes [58,59,60]. While structural methods like X-ray crystallography are fundamental to analyzing antibody–antigen complexes, computational approaches are gaining prominence. Studies on antigen mutations and their effects on antibody binding, utilizing resources like the SKEMPI 2.0 (https://life.bsc.es/pid/skempi2/ (accessed on 1 February 2025)) database, have provided significant insights [61,62]. These findings have been instrumental in repurposing antibodies to target evolving pathogens [63]. Computational modeling offers advantages such as predicting binding affinities, modeling complex interactions, and being more cost- and time-efficient [61,62,63]. Thus, employing the publicly accessible SKEMPI 2.0 to model mutated antigen–antibody interactions presents a promising in silico approach.

Finally, human APOBEC3 enzymes play a significant role in MPXV infections by their mutagenic activity, affecting viral evolution, immune evasion, and outbreak dynamics [10,64]. This phenomenon was evident in the 2022 outbreak, where genomic analyses showed frequent C-to-T transitions at specific motifs, indicating APOBEC3-mediated editing [64,65]. These mutations likely play a crucial role in the virus’s adaptive evolution, affecting its transmissibility and virulence [9]. Future in silico and laboratory investigations into these interactions and mutations are crucial for devising effective strategies to prevent and treat mpox.

## 4. Materials and Methods

### 4.1. MPXV-Customized Reference Database and Genome Dataset (2022–2024)

**Figure 7:**
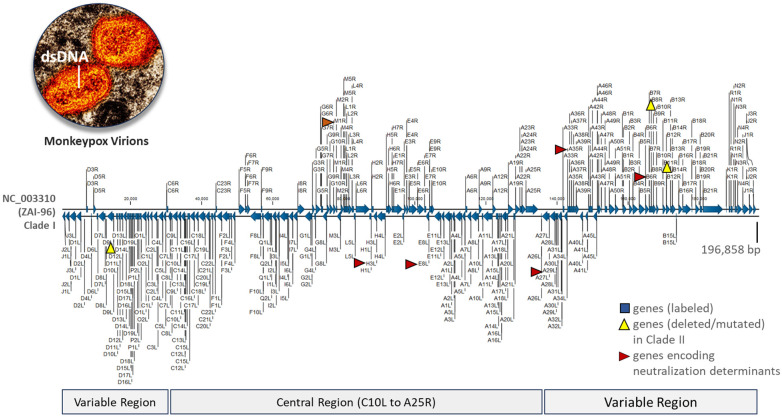
Representative monkeypox virus genome. The linear reference genome (NC_003310) from the ZAI-96-I-16 (MPV-ZAI) isolate was collected from a patient during the 1996 outbreak in Zaire (now DRC) [3,4]. This 196,858 bp double-stranded DNA sequence encodes 190 open reading frames (ORFs), featuring a highly conserved central region and bilateral terminal variable regions enclosed in hairpin loops. ORFs (*A29L*, *A35R*, *B6R*, *E8L*, *H3L*, *M1R*) encoding the antigenic proteins, homologous to those of the vaccinia virus, are indicated by red arrows [37]. Three genes that are either deleted (*D14L*) or mutated (*B10R* and *B14R*) in the less virulent prototypic Clade II MPXV isolate (SL-V70) compared to Clade I are marked by yellow triangles [4]. An electron micrograph of two MPXV virions reveals the genome-containing dumbbell-shaped inner core [67].

Our initial MPXV genome reference database included the MPXV RefSeq and 217 additional genomes obtained from NCBI Virus (https://www.ncbi.nlm.nih.gov/labs/virus/vssi/#/ (accessed on 26 June 2024)) [6,66]. These sequence files were derived from human (*n* = 203) and animal (*n* = 15) hosts, with collection dates spanning from 1958 to July 2022. For this study, we conducted another search in the NCBI Virus repository for complete MPXV genomes under Taxid: 10244, restricting the release dates from July 2022 to June 2024. After identifying the genomes and their accession numbers (ANs), we downloaded the raw sequencing files and metadata directly into CLC Genomics Workbench Premium 23.0.4 (Redwood City, CA, USA) using the “Search for Sequences at NCBI” tool with the search term “monkeypox virus complete genome”. The genome files (*n* = 1903) underwent WGA, annotation, and clade/sub-lineage determination, as described in Section 2.3. The annotations adhered to the Clade I MPXV RefSeq (NC_003310) convention. The clade/sub-lineage nomenclature is based on two publications, as the International Committee on Taxonomy of Viruses has not classified beyond the species level [5,6,40]. Figure 7 displays the MPXV RefSeq along with six genes encoding major neutralization determinants that are homologous to those of VACV [6,37].

### 4.2. Vaccinia Virus (VACV) Genome Dataset and Major Neutralization Determinants

The vaccinia virus genome dataset was compiled by searching the NCBI Virus repository (https://www.ncbi.nlm.nih.gov/labs/virus/vssi/#/ (accessed on 26 June 2024)) for complete vaccinia virus genomes, using the term “vaccinia virus” [66]. After identifying both historical and currently relevant genomes (*n* = 9), we downloaded the raw sequencing files and metadata directly into CLC Genomics Workbench using the “Search for Sequences at NCBI” tool with NCBI GenBank ANs. The genomes downloaded were VACV RefSeq (NC_006998), VACV-WR (Western Reserve) (AY243312), VACV-Tian Tan (AF095689), VACV-Copenhagen (M35027), VACV-Acam2000 (AY313847), VACV-Lister (OR837118), VACV-LC16m8 (Lister Clone 16, m8) (AY678275), VACV-MVA (Modified Vaccinia Ankara) (U94848), and VACV-MVA-BN (modified vaccinia Ankara–Bavarian Nordic) (DQ983238). Additionally, the CPXV and HPXV genomes—(NC_003663.2), CPXV-GRI-90 (X94355), and HPXV RefSeq (NC_066642)—were downloaded and used as an outgroup of ancestral pox genomes for lineage tracing and directionality in the phylogenomic analyses. The vaccinia virus genome dataset then underwent WGA, PWC, and phylogenomic analysis, as described in Section 2.3. Notably, the NCBI RefSeq for VACV and CPXV originated from the VACV-WR and CPXV Brighton Red strains, respectively [66]. As for the naming convention of VACV ORF or genes, it is based on the Copenhagen strain using three characters, with letters A to O for HindIII restriction fragments, numbers 1 to 56 for ORFs within these fragments, and R or L for transcription direction (omitted for corresponding proteins) [38].

Functionally, the six antigenic VACV membrane proteins are crucial for cell attachment and entry [38]. MV proteins A27, D8, and H3 bind to heparan sulfate or chondroitin sulfate for cell attachment. The MV L1 protein, part of the entry–fusion complex (EFC), is essential for cell entry through fusion with the plasma or endocytic membrane. A33 and B5 proteins are present on both EV and MV. EV, less abundant than MV, is involved in cell-to-cell spread within the host. EV adheres to cell surfaces to prevent superinfection through a “repulsion mechanism” induced by A33. Finally, B5 disrupts the plasma membrane to aid EV entry. The corresponding proteins for VACV are A27, A33, B5, D8, H3, and L1, and for MPXV, they are A29, A35, B6, E8, H3, and M1 [37].

### 4.3. Whole-Genome Alignment, Pairwise Comparison, and Phylogenomic Analysis

CLC Genomics Workbench Premium 23.0.4 (Redwood City, CA, USA) was installed on an HP notebook with Windows 10, an Intel i7-7500U processor, and 8 GB RAM. The “Whole Genome Alignment” plugin tools were used for automated data analysis, involving three steps, namely (1) importing sequences, (2) selecting alignment parameters, and (3) optionally copying annotations from a reference genome (Supplementary Figure S9A,B). The WGA tool functions by identifying seeds (short nucleotide sequences) shared across multiple genomes. These sequences are then extended using a scoring system known as the HOXD scoring matrix to find the best matches. This process culminates in the creation of a distance matrix, which highlights the similarities or differences among the input genomes [68]. The default parameters for our analyses are shown in Supplementary Figure S9B. The WGA output displays aligned synteny blocks, which are conserved DNA regions across genomes connected by a phylogenetic tree.

The WGA output file was inputted into the “Create Average Nucleotide Identity Comparison” tool to quantify genome similarity (Supplementary Figure S9A,C). For each genome pair, the aligned regions were identified to calculate two metrics, which were (1) alignment percentage (AP), which is the average percentage of alignment between two genomes, and (2) average nucleotide identity (ANI), which is the average percentage of matching nucleotides in the aligned regions. The AP or ANI pairwise comparison table generated was utilized in the “Create Tree from Comparison” tool (Supplementary Figure S9A) to build a NJ tree. The MPXV genome trees were initially constructed using the NJ method. For a more refined analysis of Clade I genomes, ML trees were created after determining the optimal substitution model with the CLC “Model Testing” tool. The final ML trees were built using the general time reversible (GTR) substitution model, with tree reliability assessed by the bootstrap method with 100 replicates. Due to the computational and time-intensive nature of the ML method, only batches of fewer than 30 whole MPXV genomes could be processed at a time. The main differences between NJ and ML methods are that NJ is a distance-based approach, less computationally intensive, and suitable for large datasets. In contrast, ML is a model-based method that utilizes maximum likelihood estimation, which is more accurate [69]. Finally, the geographical map of sample collection sites was generated using Wolfram Mathematica 13.0 (Champaign, IL, USA).

### 4.4. Antigenic Protein Structure Modeling

The “Download 3D protein structure database” tool was used first to install the necessary database within CLC. The “Find and Model Structure” tool in the CLC Protein Analysis tool kit (Supplementary Figure S9A) was used for the protein structural modeling of MPXV neutralization determinants. A homology-based model of an amino acid (AA) sequence of interest was generated using a BLAST-identified template from within the CLC Genomics Workbench Premium 23.0.4. The workflow included the following four steps: (1) importing the AA sequence, (2) performing a BLAST search against the protein structure sequence database, (3) filtering out low-quality hits (identity < 20%) and poor resolution (<4 Å) PDB structures, and (4) ranking the structures. The output lists the available Protein Data Bank (PDB) (https://www.rcsb.org/ (accessed on 23 August 2024)) [44] structures for creation of the structural model, BLAST statistics, and model rankings. The PDB was also searched for crystallographic structures of VACV MV (A27, D8, H3, L1) and EV (A33, B5) proteins. Structures preferably complexed with targeted antibodies were used for reconstructing and visualizing epitope/paratope binding sites in CLC Genomics Workbench.

## 5. Conclusions

This study has significantly enhanced our understanding of the distinct evolutionary paths of MPXV and VACV genomes. It has also identified important structural differences in key neutralization determinants, which are valuable for vaccine design. The workflows for viral whole-genome analysis and homology-based structural modeling will facilitate discoveries and advancements in MPXV research and vaccinology. Despite some limitations, the findings provide a solid foundation for future research. Looking ahead, the use of advanced computational modeling holds promise to yield deeper insights into MPXV antigen–antibody interactions.

## Figures and Tables

**Table 1:** Summary of structures found for MPXV Clade I (NC_003310) antigenic proteins.

MPXV Virion	MPXV Gene (Protein)	Structures Found (*n*)	Available Structures ^1^	Rank ^2^	E-Value	Match ID (%)	Coverage (%)
MV	*A29L* (A29)	1	3VOP ^3^	1	3.1 × 10^−20^	90.7	38.7
MV	*E8L* (E8)	1309	5USH ^4^	3	1.7 × 10^−158^	92.7	75.4
MV	*H3L* (H3)	1	5EJ0 ^5^	1	1.6 × 10^−142^	84.4	65.2
MV	*M1R* (M1)	6	2I9L ^6^	3	4.5 × 10^−125^	99.4	68.9
EV	*A35R* (A35)	7	4LU5 ^7^	5	1.1 × 10^−50^	90.4	45.1
EV	*B6R* (B6)	36	5FOB ^8^	1	1.0 × 10^−11^	29.6	67.9

EV, enveloped virion; ID, identity; MPXV, monkeypox virus; MV, mature virion; ^1^ Available crystallographic structure(s) are found in RCSB Protein Data Bank (PDB) [44]. All structures are limited to the ectodomains of the antigenic proteins. The complete table is provided in Supplementary Table S3. ^2^ The rank of the structure(s) found is scored based on homology to the query sequence and the structural quality. Specifically, the variables include three BLAST statistics (E-value, % Match identity, % Coverage), resolution (of crystal structure), and free R-value (R-free of crystal structure) [45]. ^3^ 3VOP: PDB Entry ID for structure of VACV A27 antigenic protein. ^4^ 5USH: PDB Entry ID for structure of VACV D8 antigenic protein bound to human Fab vv66. ^5^ 5EJ0: PDB Entry ID for structure of VACV H3 antigenic protein. ^6^ 2I9L: PDB Entry ID for structure of VACV L1 antigenic protein bound to Fab 7D11. ^7^ 4LU5: PDB Entry ID for structure of VACV A33 antigenic protein bound to murine IgG2a Fab A20G2. ^8^ 5FOB: PDB Entry ID for structure of smallpox inhibitor of complement (SPICE) bound to complement C3b.

## Data Availability

The MPXV and VACV genomes are publicly available from NCBI Virus (https://www.ncbi.nlm.nih.gov/labs/virus/vssi/#/) under respective Taxid numbers 10244 and 10245 (accessed on 26 June 2024). The crystallographic structures used for structural modeling are publicly available from PDB (https://www.rcsb.org/) under the respective structure IDs VACV A27 (3VOP); VACV 27-Fab 1G6 (5EOQ); VACV D8 (5USH); VACV H3 (5EJ0); VACV L1 (2I9L); VACV A33 (4LU5); and VARV (5FOB) (accessed on 23 August 2024).

## Supplementary Materials

The following supporting information can be downloaded at: https://www.mdpi.com/article/10.3390/ijms26041428/s1.

## Abbreviations

AA, amino acid; AP, alignment percentage; APOBEC3, apolipoprotein B mRNA-editing catalytic polypeptide-like 3; ANI, average nucleotide identity; CLC MGM, CLC Microbial Genomics Module; CPXV, cowpox virus; DRC, Democratic Republic of the Congo; dsDNA, double-stranded DNA; DB, database; EA IND, expanded access investigational new drug protocol; ICTV, International Committee on Taxonomy of Viruses; MPXV, monkeypox virus; NCBI, National Center for Biotechnology Information; NJ, neighbor joining; ORF, open reading frame; PHEIC, public health emergency of international concern; PDB, Protein Data Bank; PWC, pairwise comparison; RefSeq, reference sequence; RepSeq, representative sequence; VACV, vaccinia virus; VARV, variola virus; WGA, whole-genome alignment; WHO, World Health Organization.
