# Supplementary material for: Genomic and Antigenic Differences Between Monkeypox Virus and Vaccinia Vaccines: Insights and Implications for Vaccinology
Source: Int J Mol Sci. 2025 Feb 8;26(4):1428. doi: 10.3390/ijms26041428 (PMC11855751; doi:10.3390/ijms26041428)
Supplement: Supplementary file 1 [file ijms-26-01428-s001.zip › Fig S1 MPXV WGA_N243 02-07-25.pdf]

A

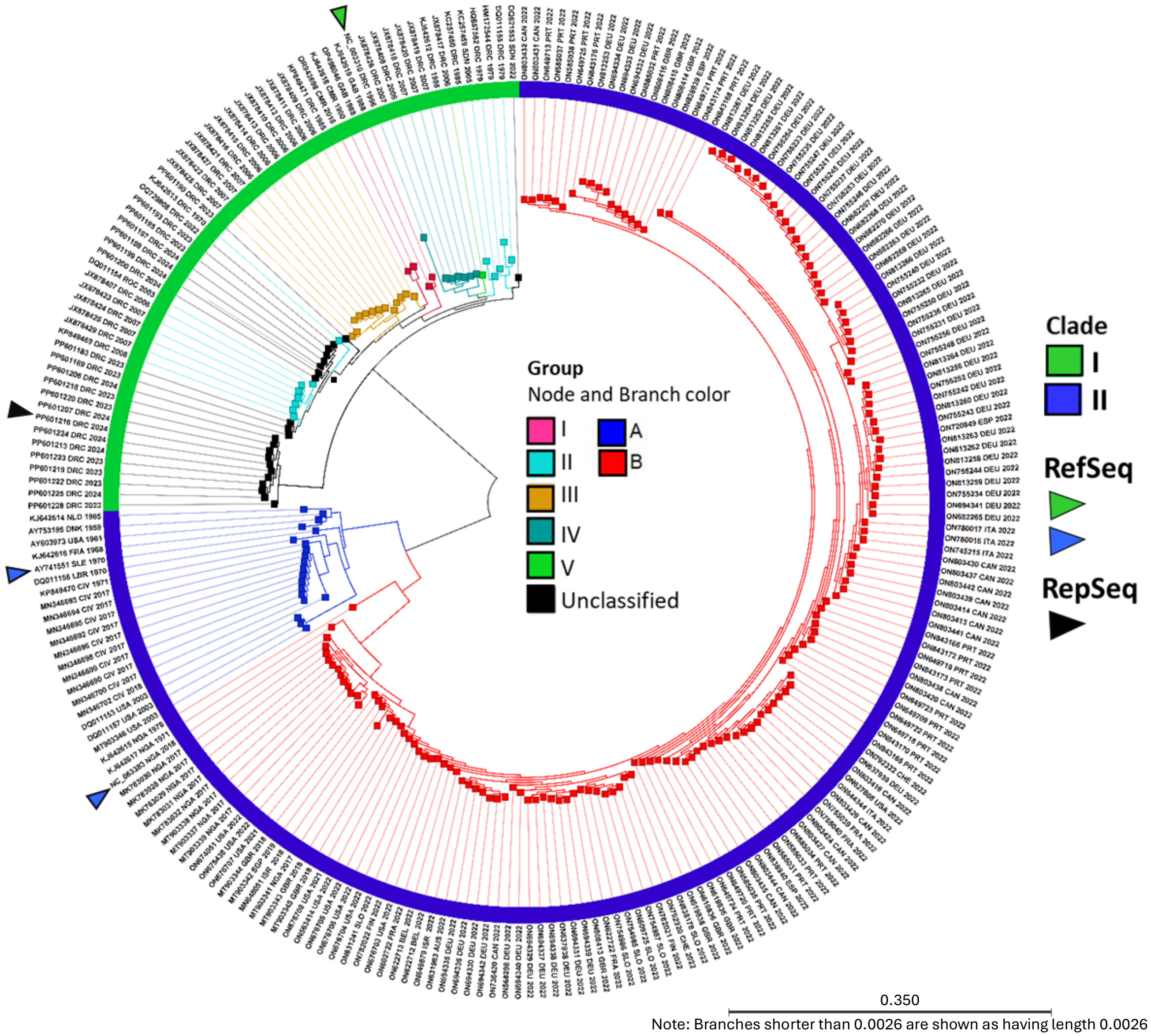
Group
Node and Branch color
I
II
III
IV
V
Unclassified
A
B
Clade
I
II
RefSeq
RepSeq
0.350
Note: Branches shorter than 0.0026 are shown as having length 0.0026

B

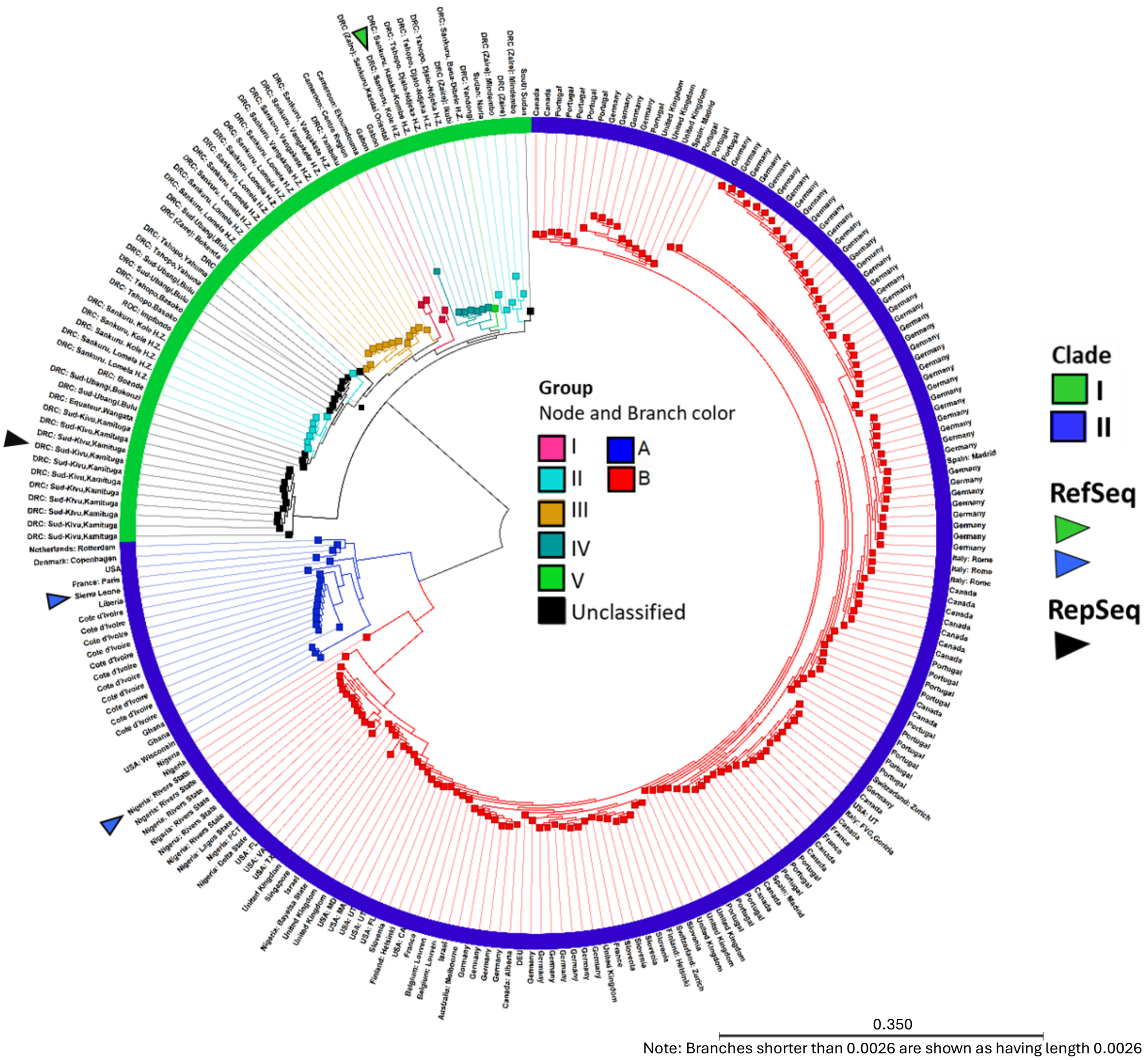

Supplementary Figure S1. Enlarged phylograms of Figures 2A (**A**) and 2B (**B**) for enhanced visualization. Refer to the Figure 2 legend for details.
