# Supplementary material for: Genomic and Antigenic Differences Between Monkeypox Virus and Vaccinia Vaccines: Insights and Implications for Vaccinology
Source: Int J Mol Sci. 2025 Feb 8;26(4):1428. doi: 10.3390/ijms26041428 (PMC11855751; doi:10.3390/ijms26041428)
Supplement: Supplementary file 1 [file ijms-26-01428-s001.zip › Fig S2 MPXV WGA N1878.pdf]

A

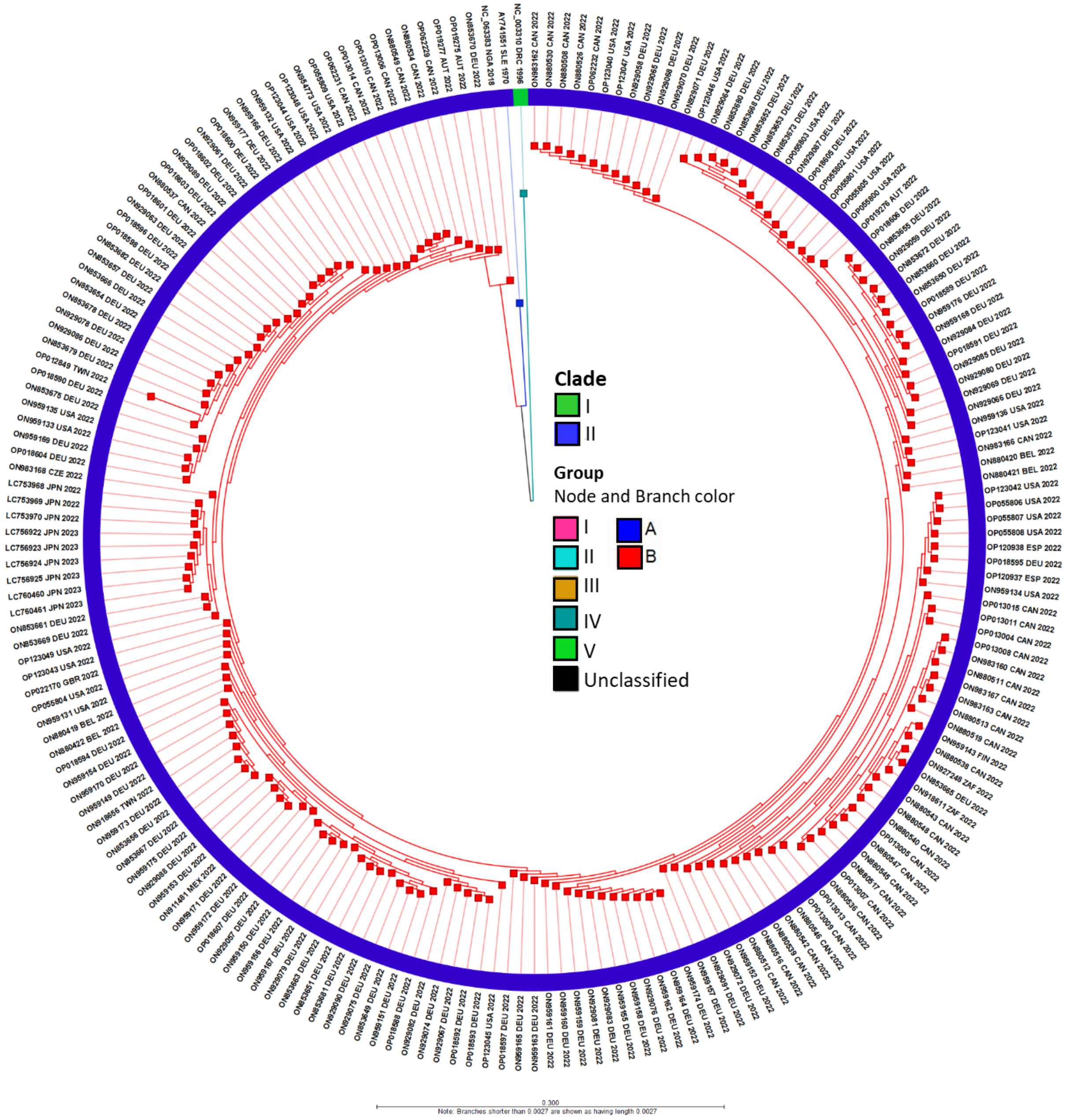
Clade
I
II
Group
Node and Branch color
I
II
III
IV
V
Unclassified
A
B
0.300
Note: Branches shorter than 0.0027 are shown as having length 0.0027

B

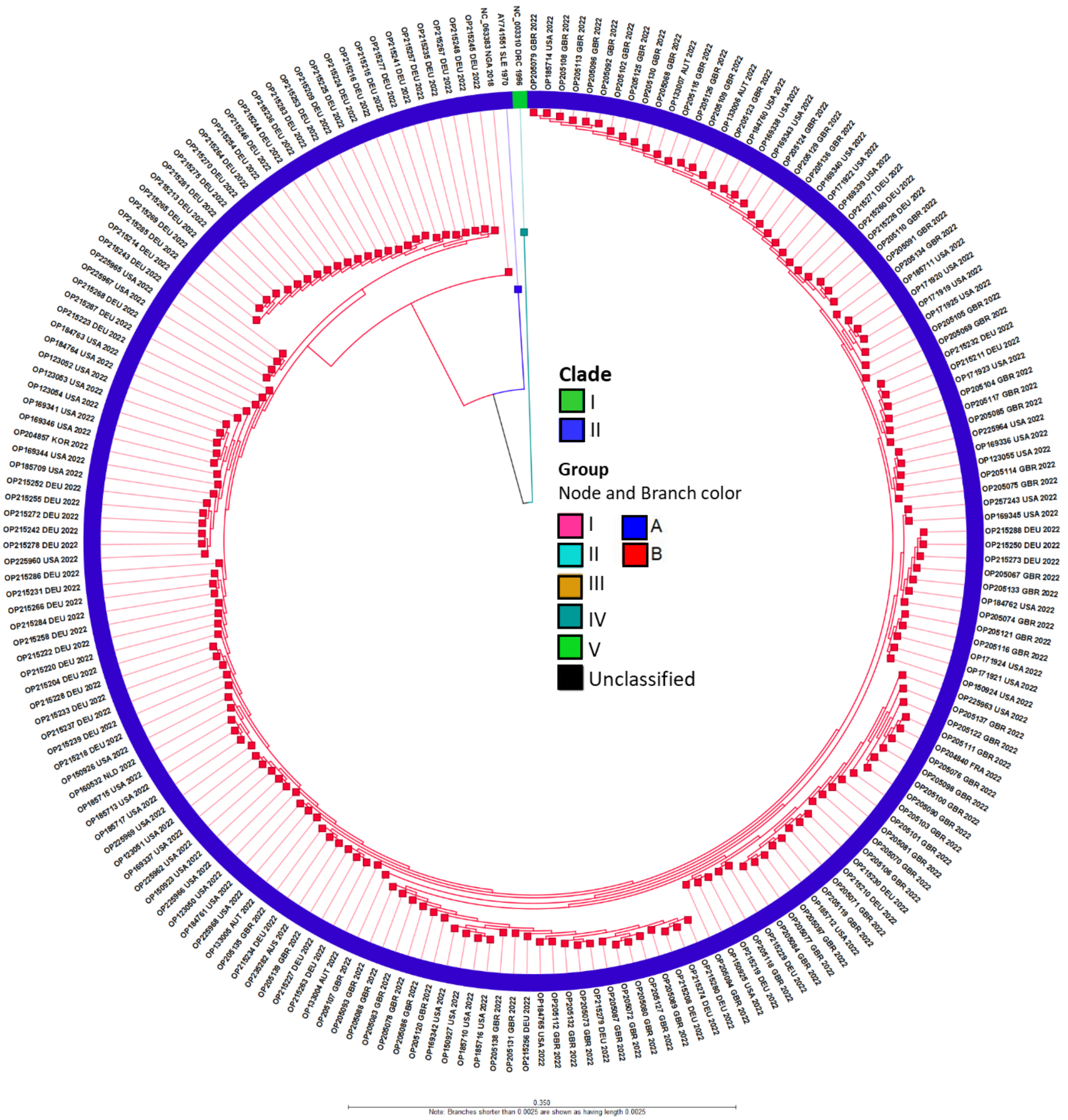
Clade
I
II
Group
Node and Branch color
I
II
III
IV
V
Unclassified
A
B
0.350
Note: Branches shorter than 0.0025 are shown as having length 0.0025

C

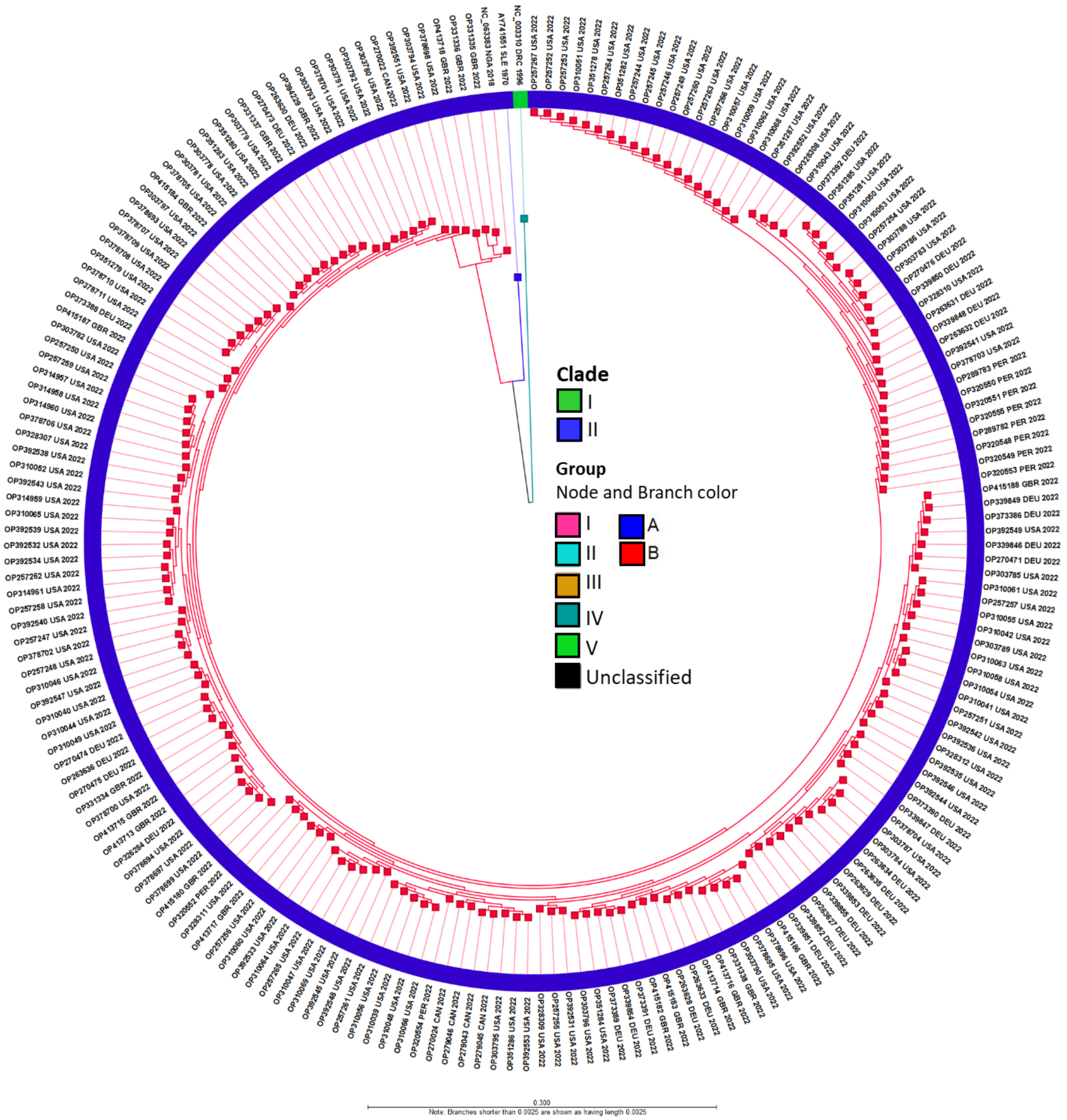
Clade
I
II
Group
Node and Branch color
I
II
III
IV
V
Unclassified
A
B
0.300
Note: Branches shorter than 0.0025 are shown as having length 0.0025

D

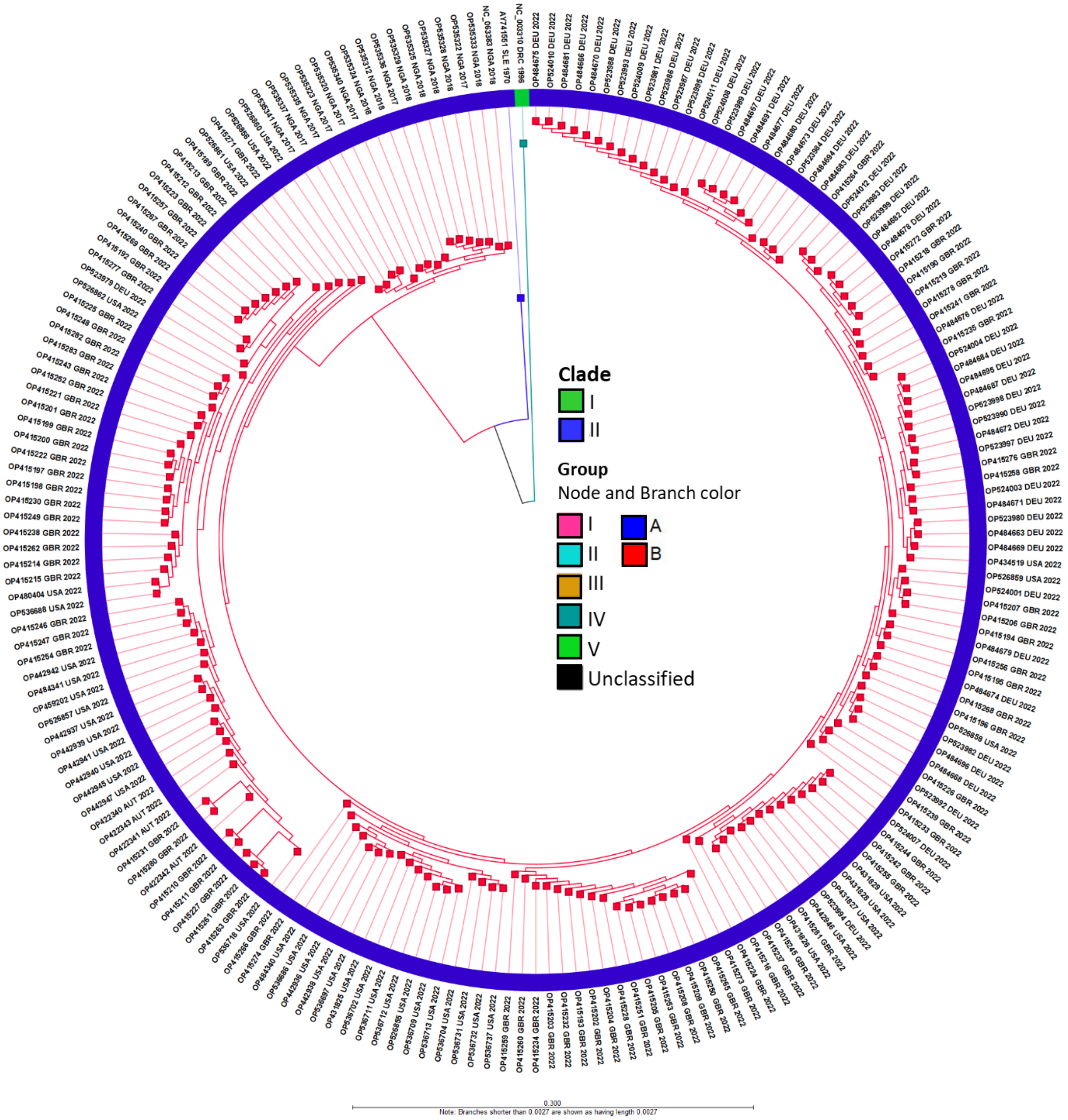
Clade
I
II
Group
Node and Branch color
I
II
III
IV
V
Unclassified
A
B
0.300
Note: Branches shorter than 0.0027 are shown as having length 0.0027

E

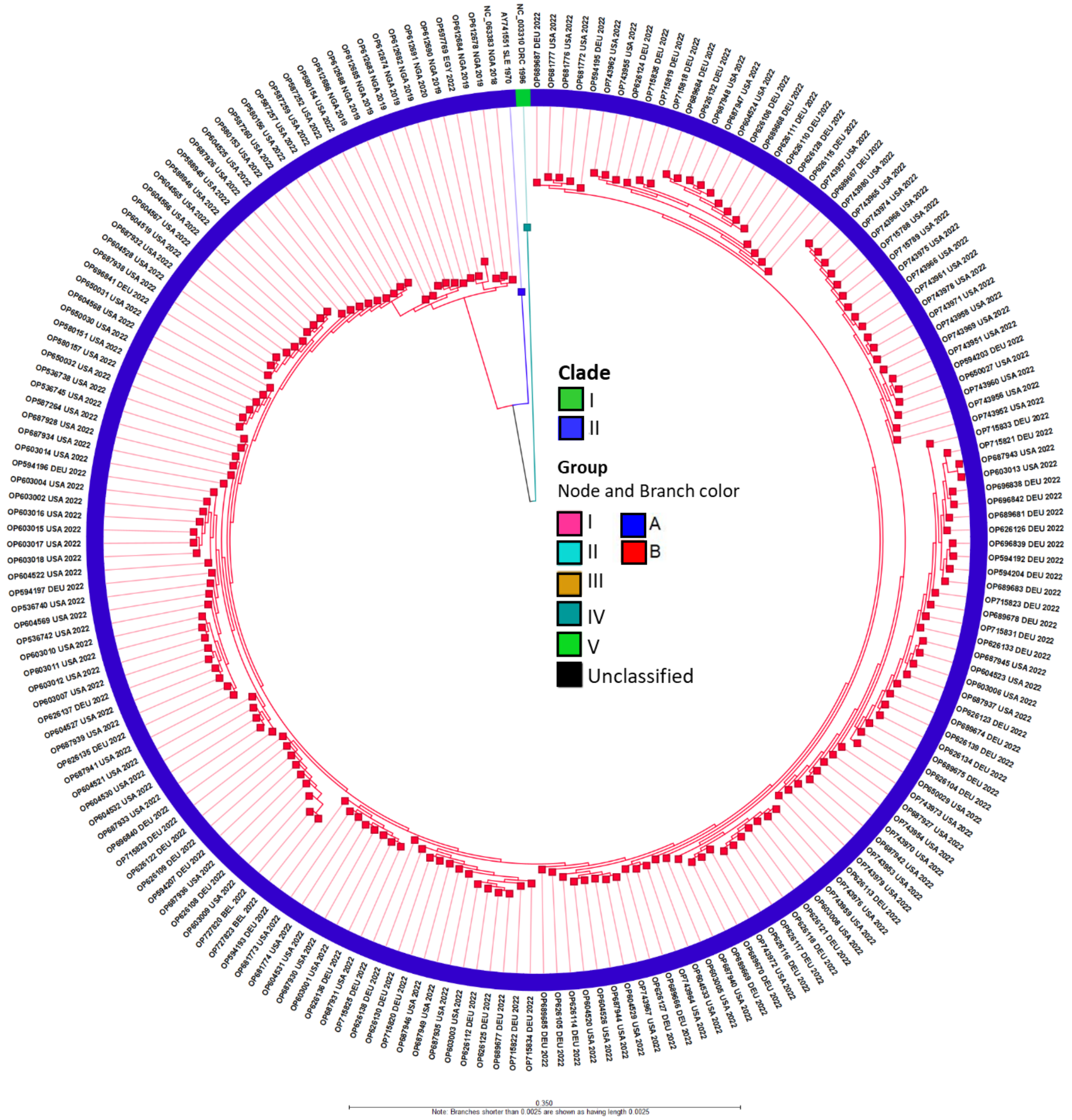

Clade
I
II
Group
Node and Branch color
I
II
III
IV
V
Unclassified
A
B
0.350
Note: Branches shorter than 0.0025 are shown as having length 0.0025

F

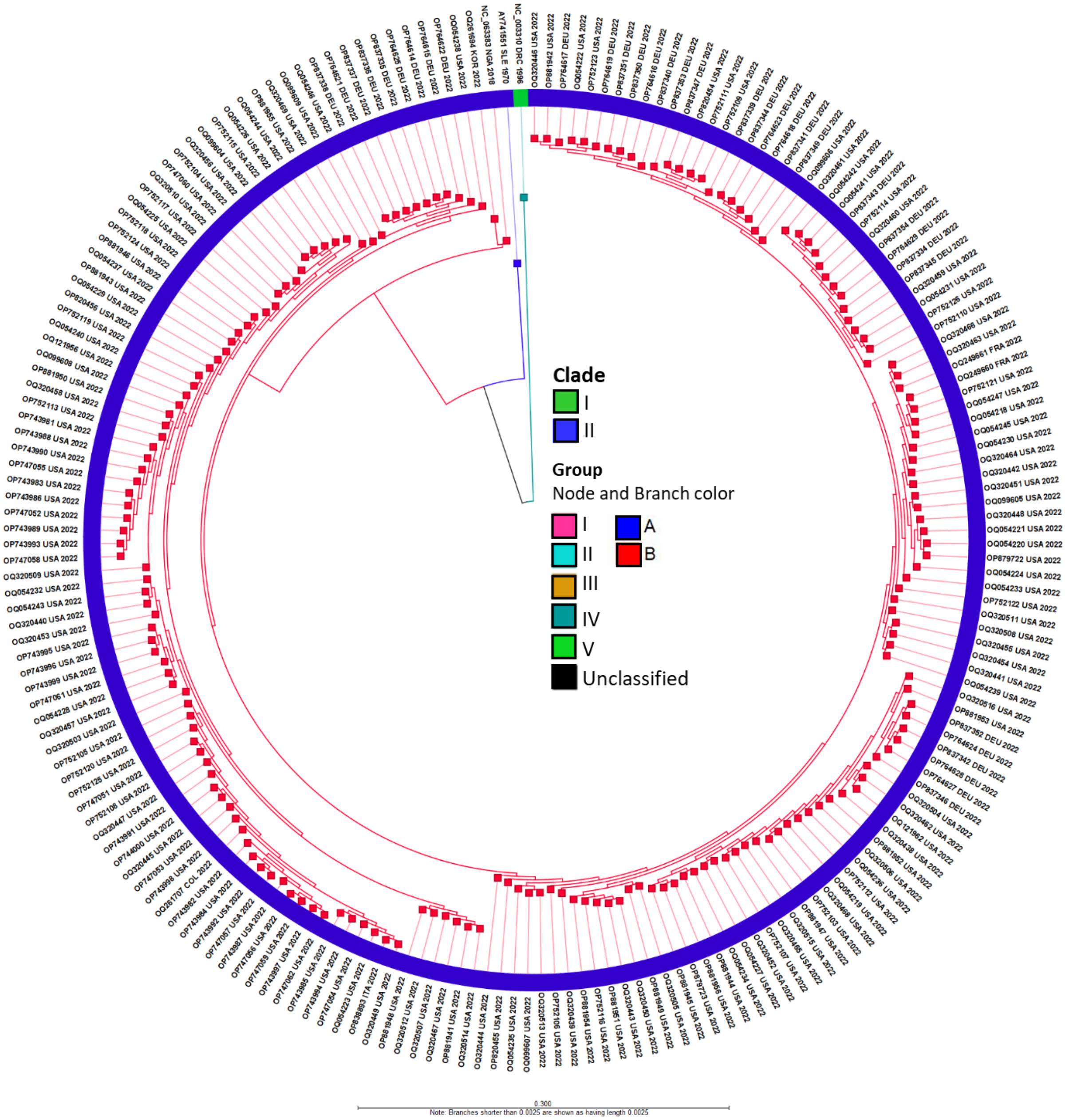
Clade
I
II
Group
Node and Branch color
I
II
III
IV
V
Unclassified
A
B
0.300
Note: Branches shorter than 0.0025 are shown as having length 0.0025

G

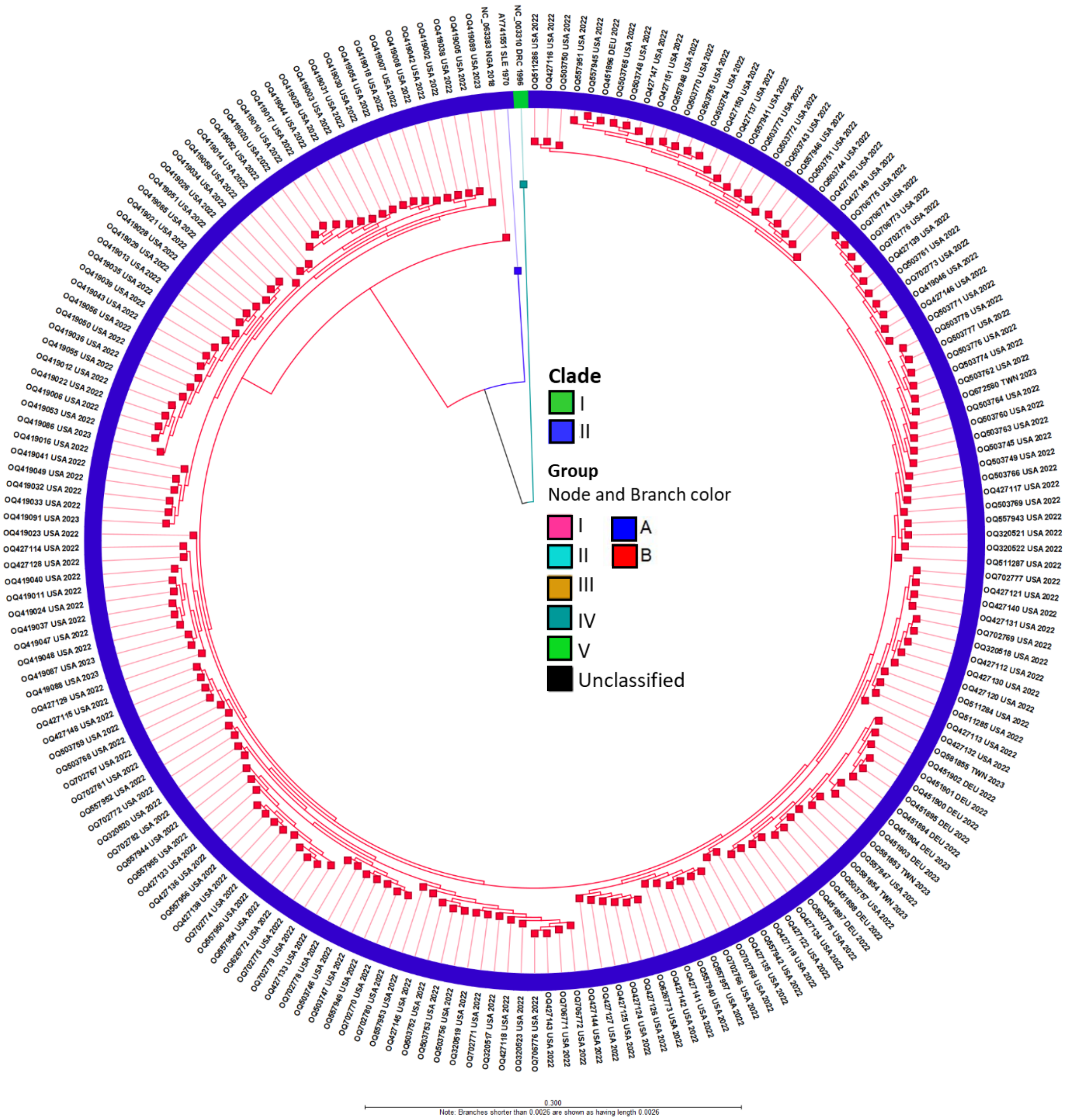

Clade
I
II
Group
Node and Branch color
I
II
III
IV
V
Unclassified
A
B
0.300
Note: Branches shorter than 0.0026 are shown as having length 0.0026

H

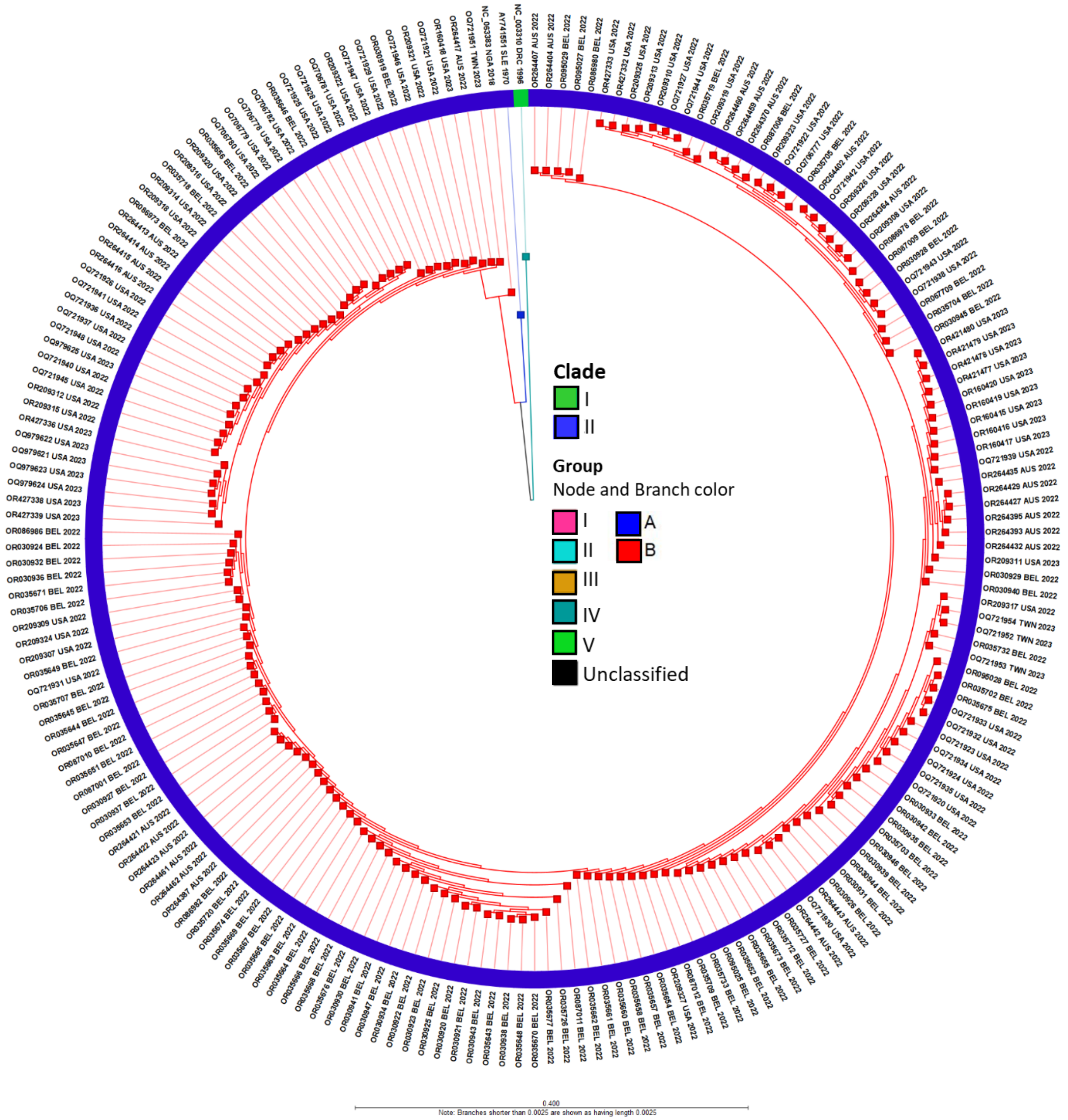
Clade
I
II
Group
Node and Branch color
I
II
III
IV
V
Unclassified
A
B
0.400
Note: Branches shorter than 0.0025 are shown as having length 0.0025

I

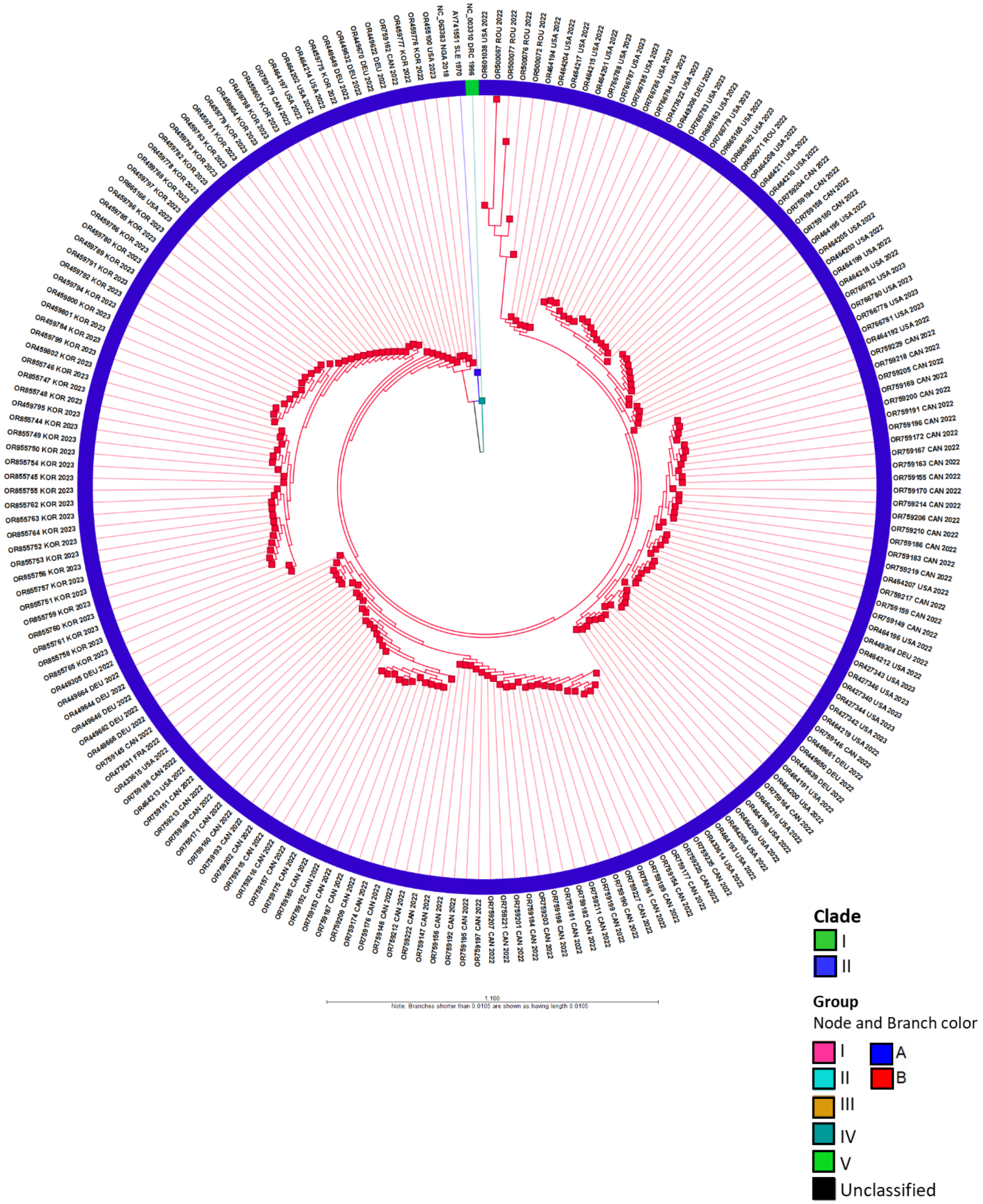

1.100
Note: Branches shorter than 0.0105 are shown as having length 0.0105
Clade
I
II
Group
Node and Branch color
I
II
III
IV
V
Unclassified
A
B

J

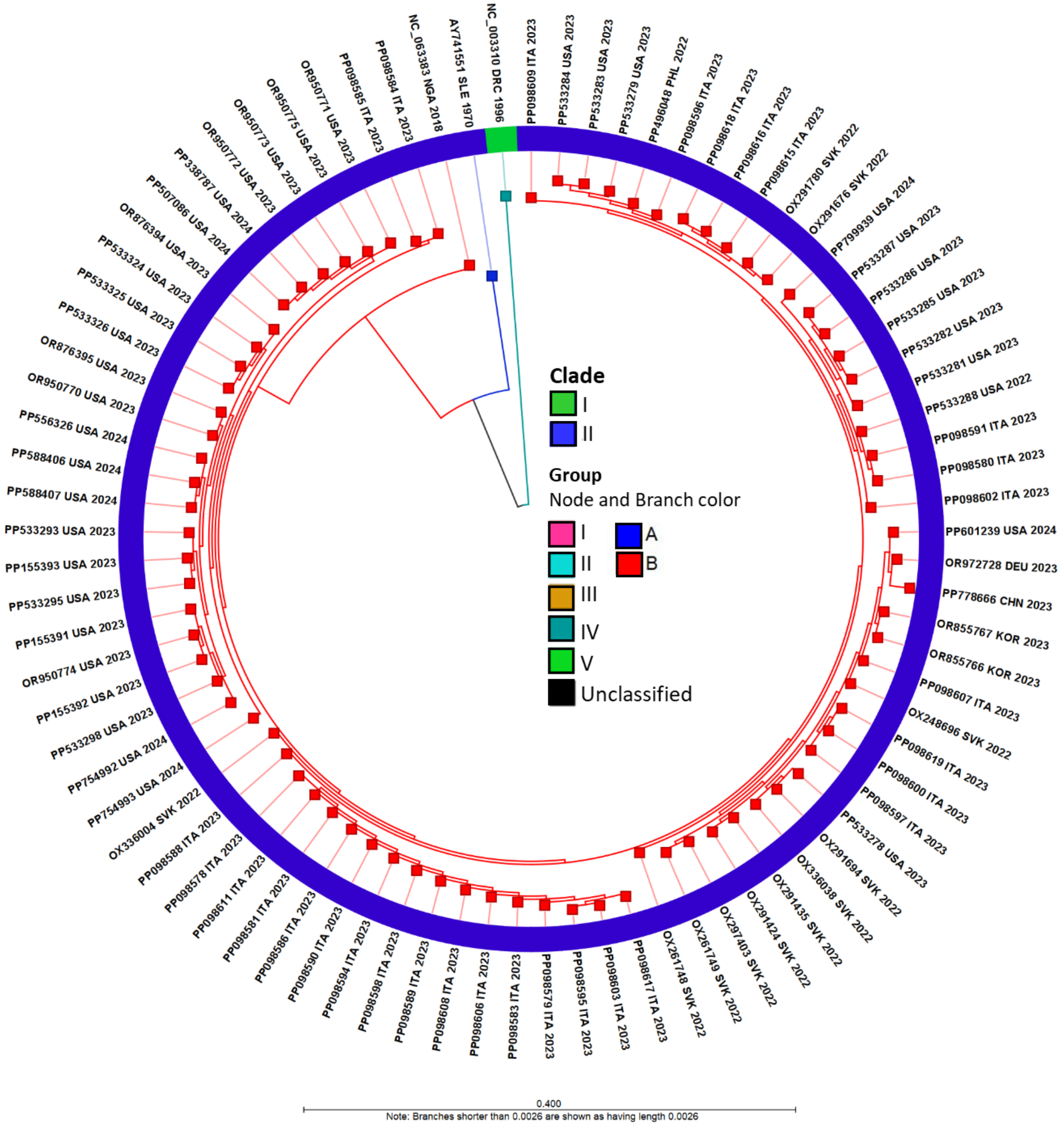

Supplementary Figure S2. Circular phylograms of 1,878 MPXV genomes clustering with Clade IIB. Three attributes of each genome (sequence accession number, 3-letter country code, and year collected) are displayed as the outermost ring for providing geo-temporal and evolutionary relationships. The MPXV reference genomes used in the alignments were from Clades I (NC_003310), IIA (AY741551), and IIB (NC_063383). (**A**) Phylogram of 199 genomes from LC753968_JPN_2022 to OP123049_USA_2022, (**B**) Phylogram of 200 genomes from OP123050_USA_2022 to OP257243_USA_2022, (**C**) Phylogram of 200 genomes from OP257244_USA_2022 to OP415188_GBR_2022, (**D**) Phylogram of 199 genomes from OP415189_GBR_2022 to OP536737_USA_2022, (**E**) Phylogram of 200 genomes from OP536738_USA_2022

to OP743980_USA_2022, (**F)** Phylogram of 200 genomes from OP743981_USA_2022 to OQ320516_USA_2022, (**G)** Phylogram of 199 genomes from OQ320517_USA_2022 to OQ706776_USA_2022, (**H)** Phylogram of 199 genomes from OQ706777_USA_2022 to OR427339_USA_2023, (**I)** Phylogram of 200 genomes from OR427340_USA_2023 to OR855765_KOR_2023, (**J)** Phylogram of 82 genomes from OR855766_KOR_2023 to PP799939_USA_2024. Genome accession numbers (alphanumeric) are listed in ascending order for (A-J). The ANI NJ unrooted trees were constructed from the pairwise comparison table.
