# Supplementary material for: Genomic and Antigenic Differences Between Monkeypox Virus and Vaccinia Vaccines: Insights and Implications for Vaccinology
Source: Int J Mol Sci. 2025 Feb 8;26(4):1428. doi: 10.3390/ijms26041428 (PMC11855751; doi:10.3390/ijms26041428)
Supplement: Supplementary file 1 [file ijms-26-01428-s001.zip › Fig S3 MPXV clade I ML trees.pdf]

A MPXV Clade 1, representative ancestral sequences from Groups I-V alongside RefSeq genome (NC_003310)

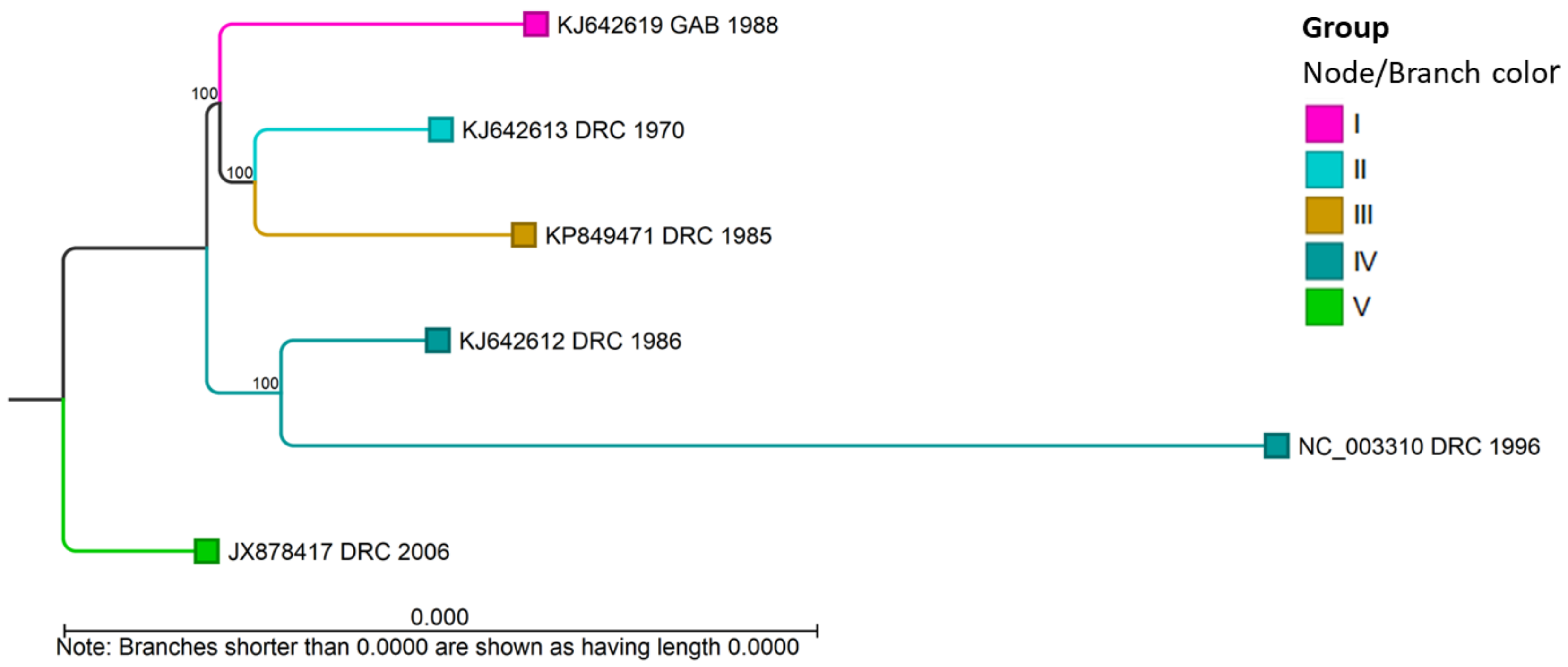

B MPXV Clade 1, Groups I and II sequences alongside representative ancestral sequences from Groups I-V (*)

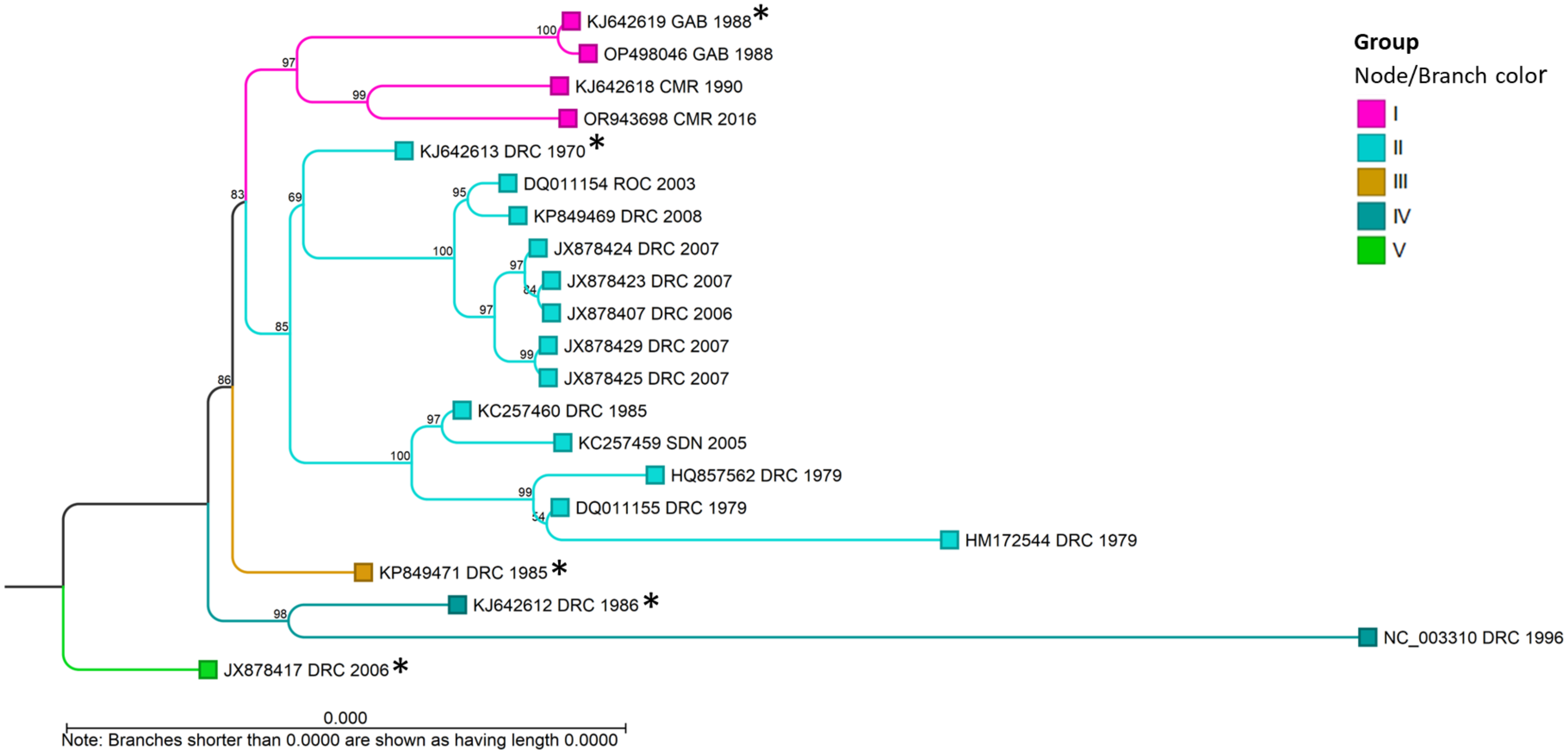

C MPXV Clade 1, Group III sequences alongside representative ancestral sequences from Groups I-V (*)

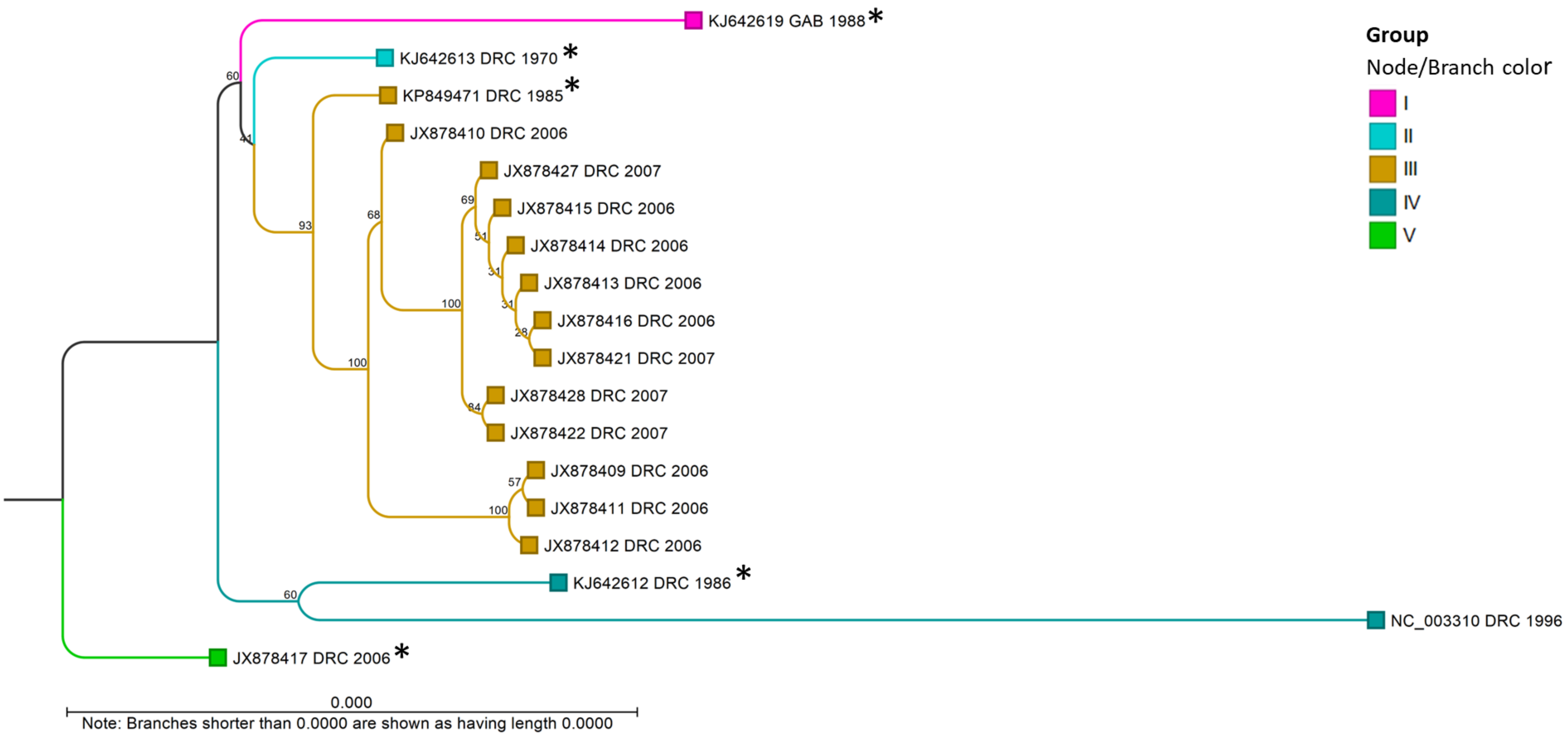

D MPXV Clade 1, Group IV sequences alongside representative ancestral sequences from Groups I-V (*)

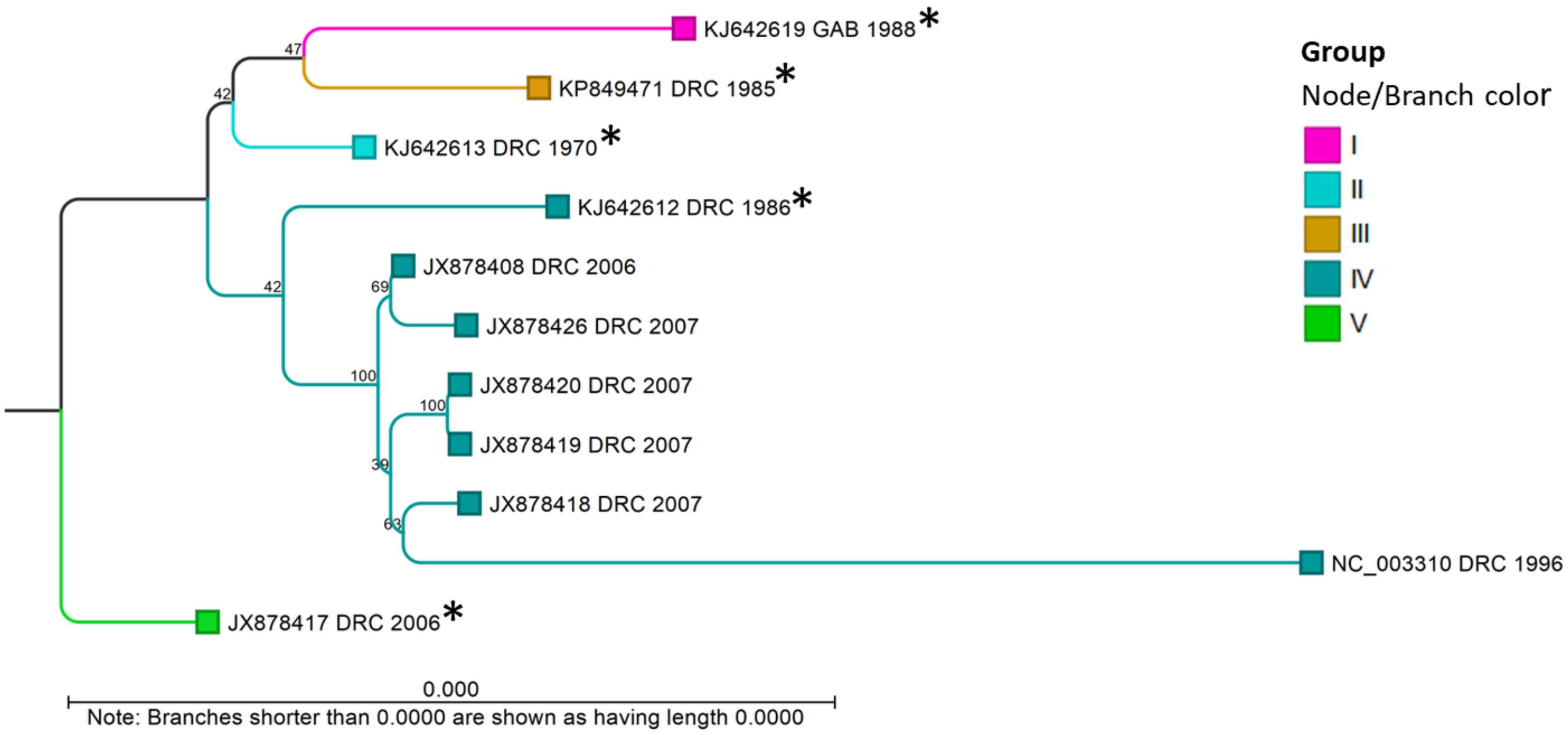

E MPXV Clade 1, unclassified sequences alongside representative ancestral sequences from Groups I-V (*)

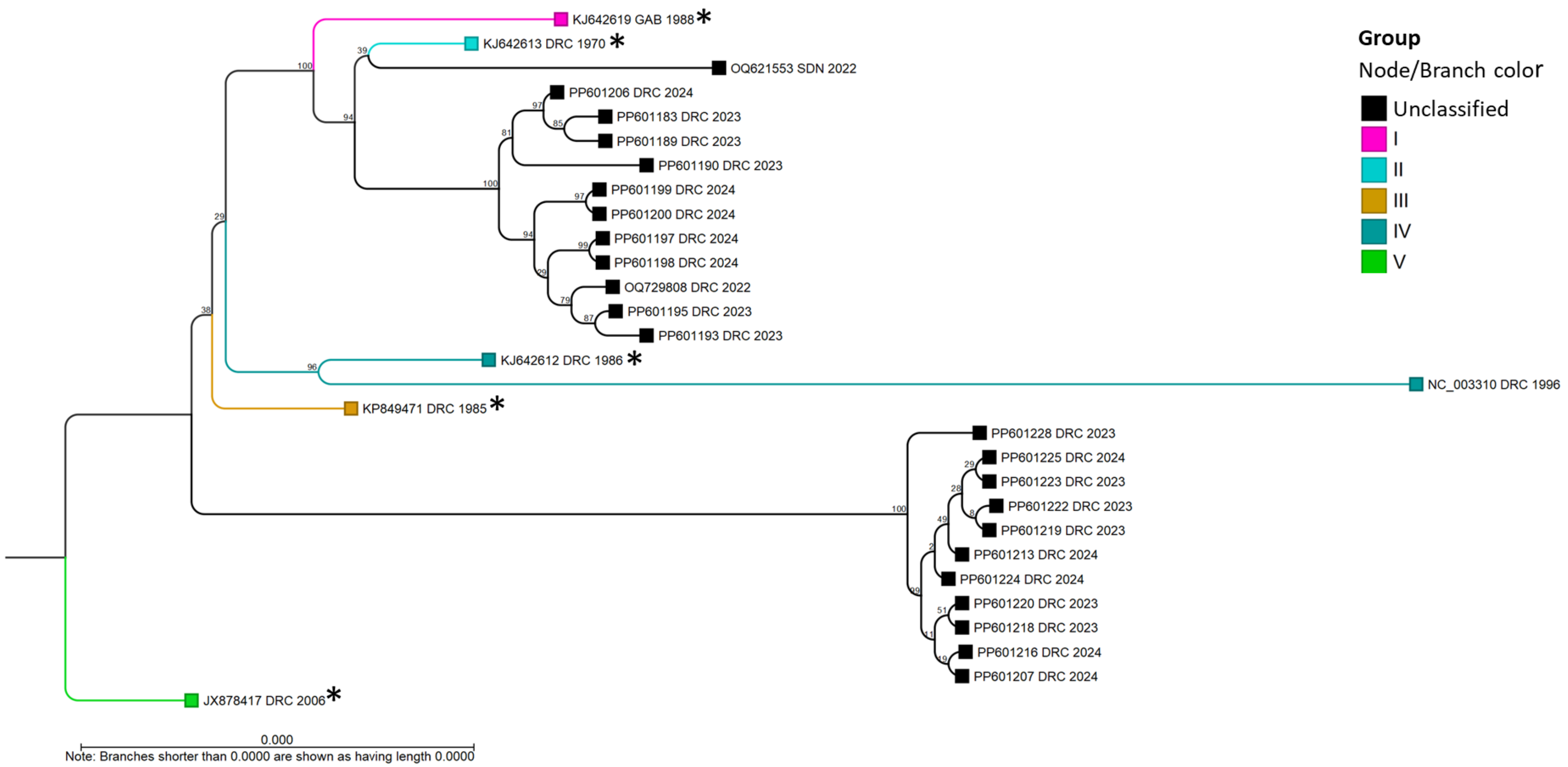

Supplementary Figure S3. Maximum Likelihood phylogeny of MPXV Clade I, classified and unclassified genomes. (**A-E)** The phylogenetic trees illustrate the genetic relationships and lineages among Clade I MPXV genomes. These Maximum Likelihood (ML) trees were constructed using the General Time Reversible (GTR) substitution model, with tree reliability estimated by the bootstrap method with 100 replicates; (**A)** The ML tree of the most ancestral sequences from Groups I-V depicts the overall relationships among the groups. The Clade I RefSeq (NC_003310), commonly used as a reference genome, is aligned under Group IV; (**B**) The ML tree of Group I and II sequences, including

group-specific ancestral sequences, shows the bifurcation of Group I and II into two sub-lineages; (**C)** The ML tree of Group III sequences, including group-specific ancestral sequences, shows a monophyletic clade; (**D)** The ML tree of Group IV sequences, including group-specific ancestral sequences, shows a monophyletic clade; (**E)** The ML tree of new, unclassified sequences shows twelve genomes clustering with Group II, while eleven genomes form a new sub-lineage descending from Group V.
