# Supplementary material for: Genomic and Antigenic Differences Between Monkeypox Virus and Vaccinia Vaccines: Insights and Implications for Vaccinology
Source: Int J Mol Sci. 2025 Feb 8;26(4):1428. doi: 10.3390/ijms26041428 (PMC11855751; doi:10.3390/ijms26041428)
Supplement: Supplementary file 1 [file ijms-26-01428-s001.zip › Fig S4 VACV w OUTGRP_02-07-25.pdf]

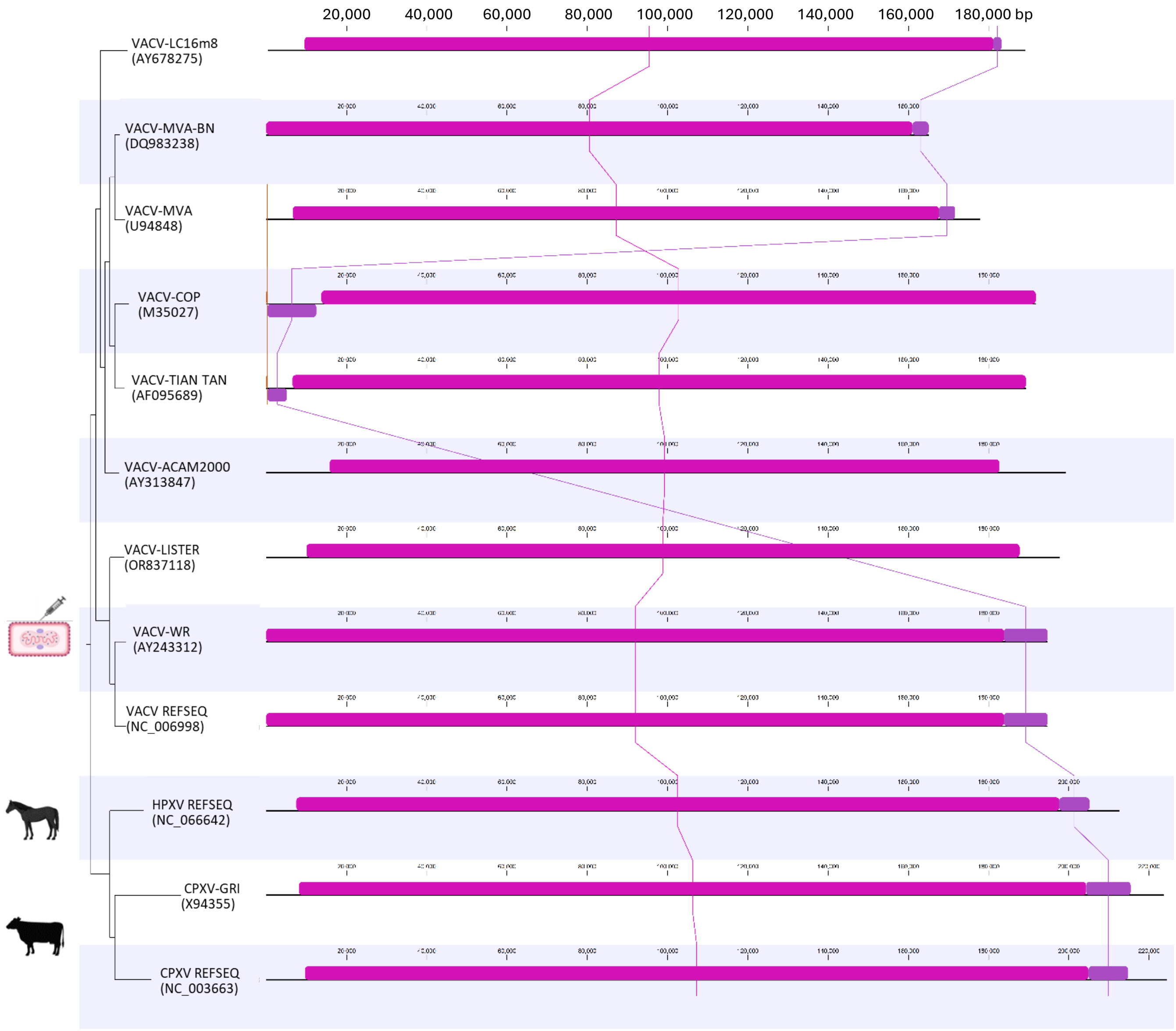

Supplementary Figure S4. Whole genome alignment of Vaccinia, Horsepox and Cowpox viral genomes. The depicted genome alignment of 9 representative VACV, 2 CPXV, and 1 HPXV genomes revealed a conserved central region (magenta), but variable terminal synteny blocks (violet and brown) and genome lengths. The longest VACV genome was VACV-ACAM2000 (199,234 bp), while the shortest was VACV MVA-BN (165,041 bp). The VACV RefSeq genome length (194,711 bp) was shorter by 17,922-29,788 bp than the RefSeqs for HPXV (212,633 bp) and CPXV (224,499 bp). The distal synteny block beyond the 200,000 bp position identified in the CPXV, HPXV, and VACV RefSeqs was truncated, rearranged, or deleted in subsequent generations of VACV. Notably, the genes for the 6 neutralization determinants were identified in the genomes of two current vaccines, VACV MVA-BN and LC16m8.
