# Supplementary material for: Genomic and Antigenic Differences Between Monkeypox Virus and Vaccinia Vaccines: Insights and Implications for Vaccinology
Source: Int J Mol Sci. 2025 Feb 8;26(4):1428. doi: 10.3390/ijms26041428 (PMC11855751; doi:10.3390/ijms26041428)
Supplement: Supplementary file 1 [file ijms-26-01428-s001.zip › Fig S5 MPXV Ag Models_backbone.pdf]

A MPXV Clade 1, Group IV, Protein model A29 (NC_003310)

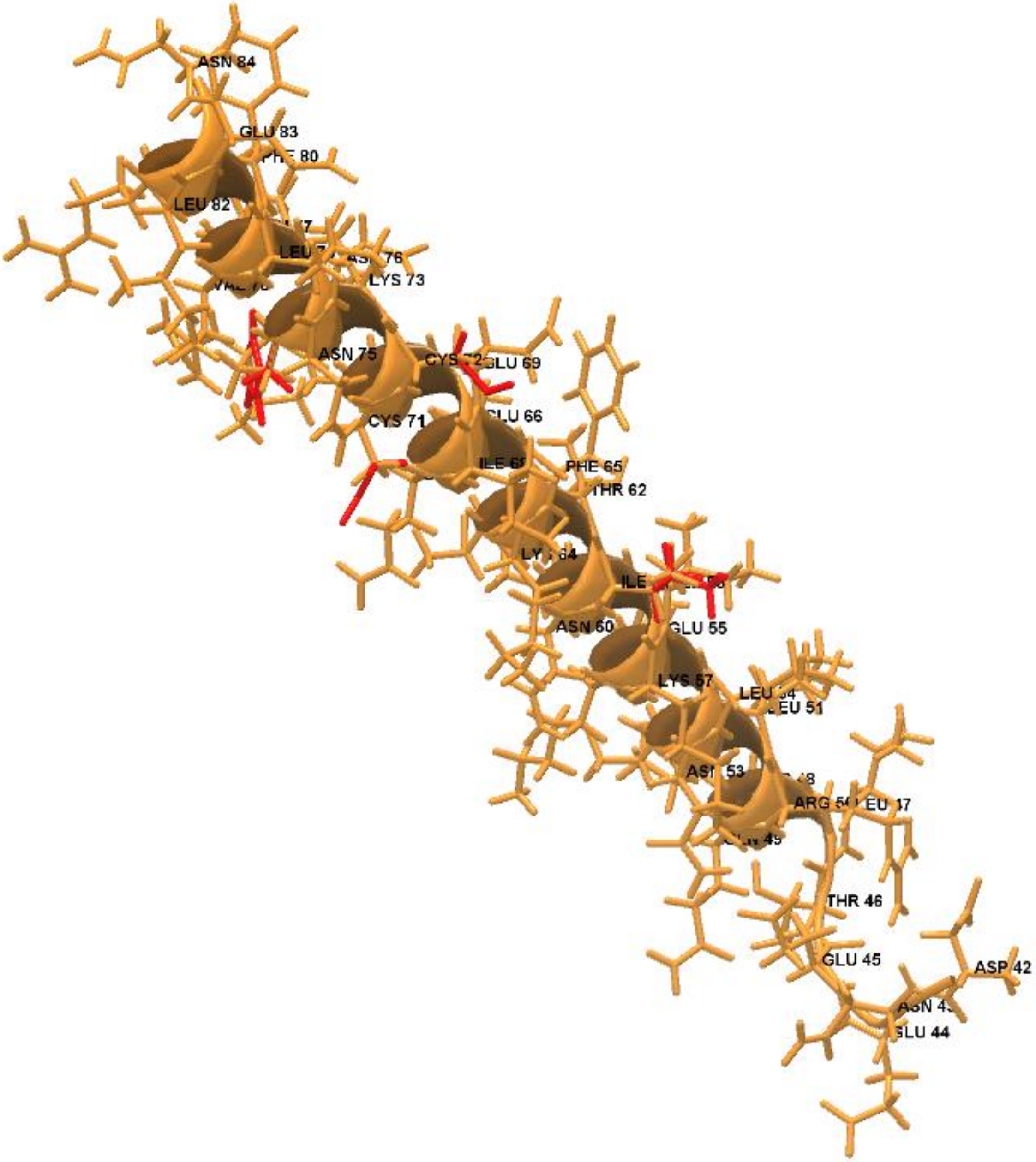

B MPXV Clade 1, Group IV, Protein model E8 (NC_003310)

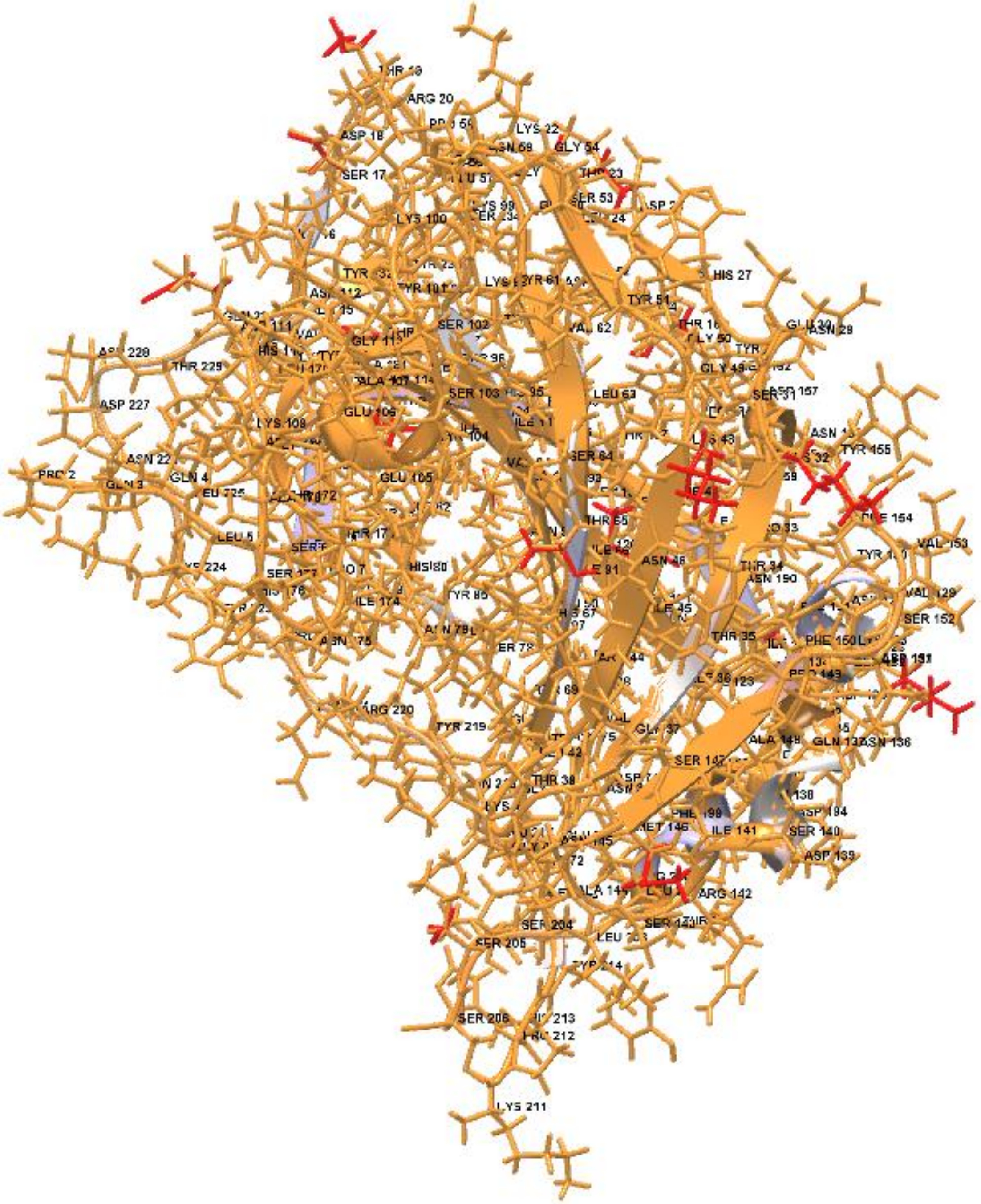

C MPXV Clade 1, Group IV, Protein model H3 (NC_003310)

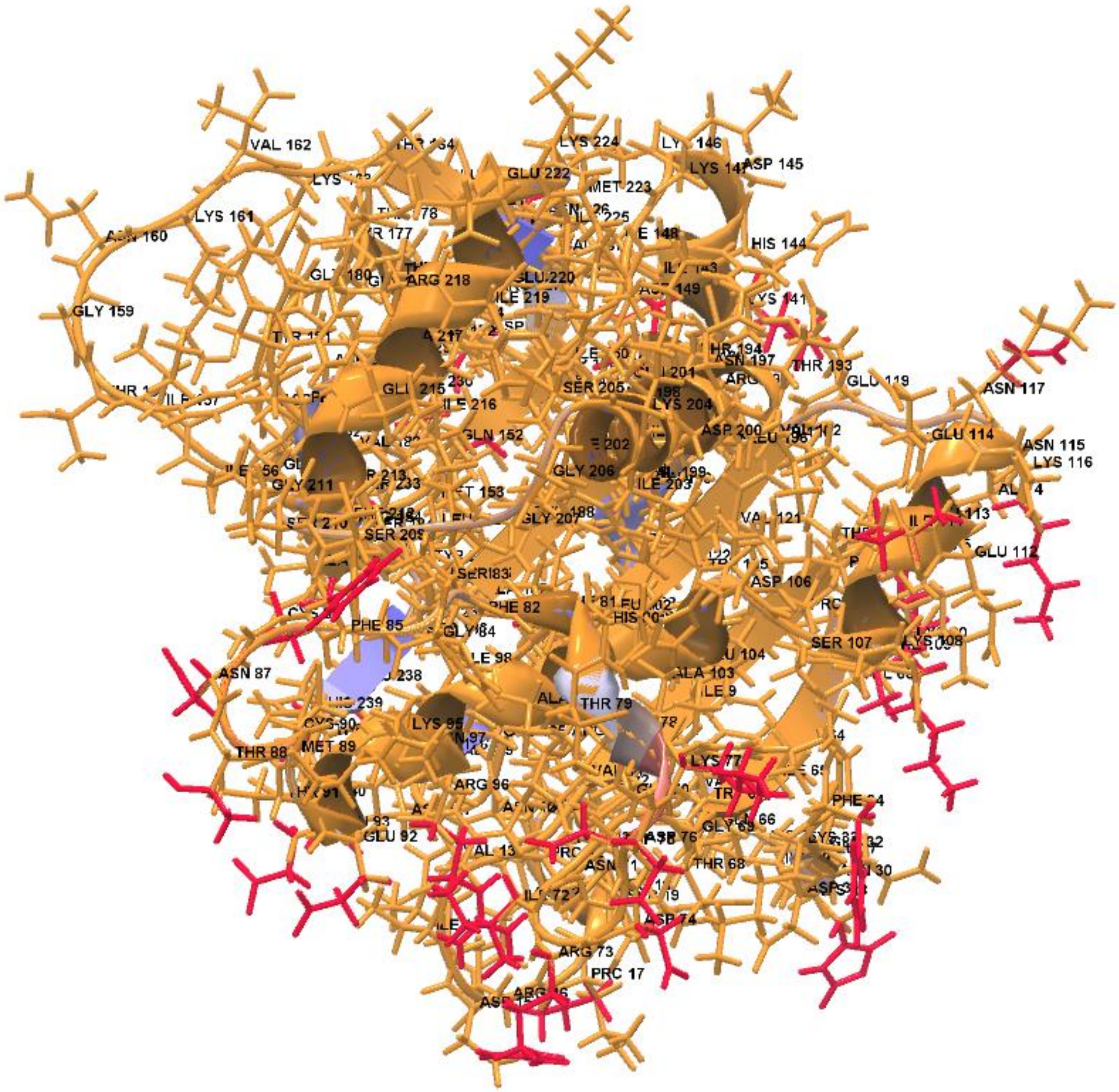

D MPXV Clade 1, Group IV, Protein model M1 (NC_003310)

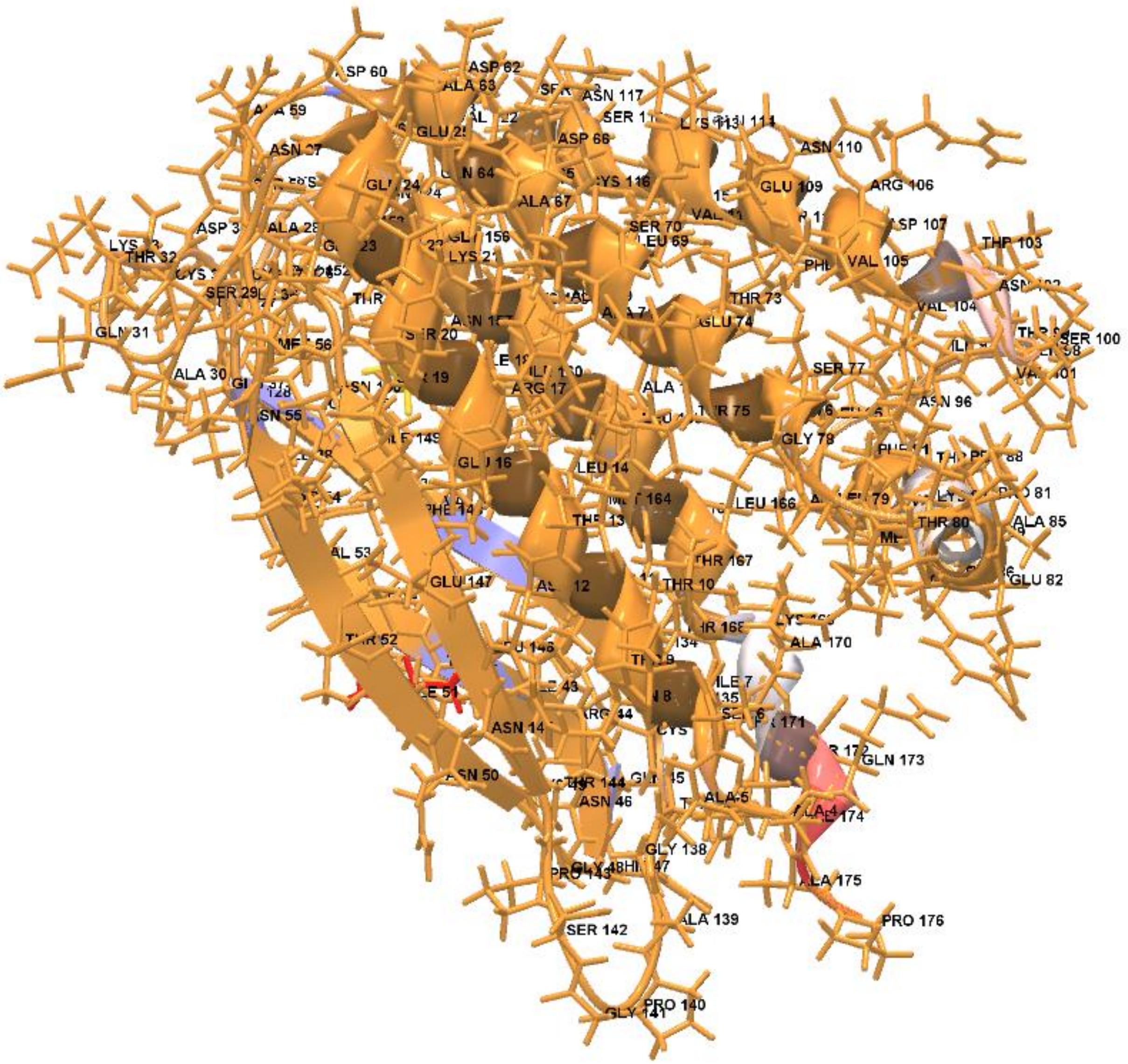

E MPXV Clade 1, Group IV, Protein model A35 (NC_003310)

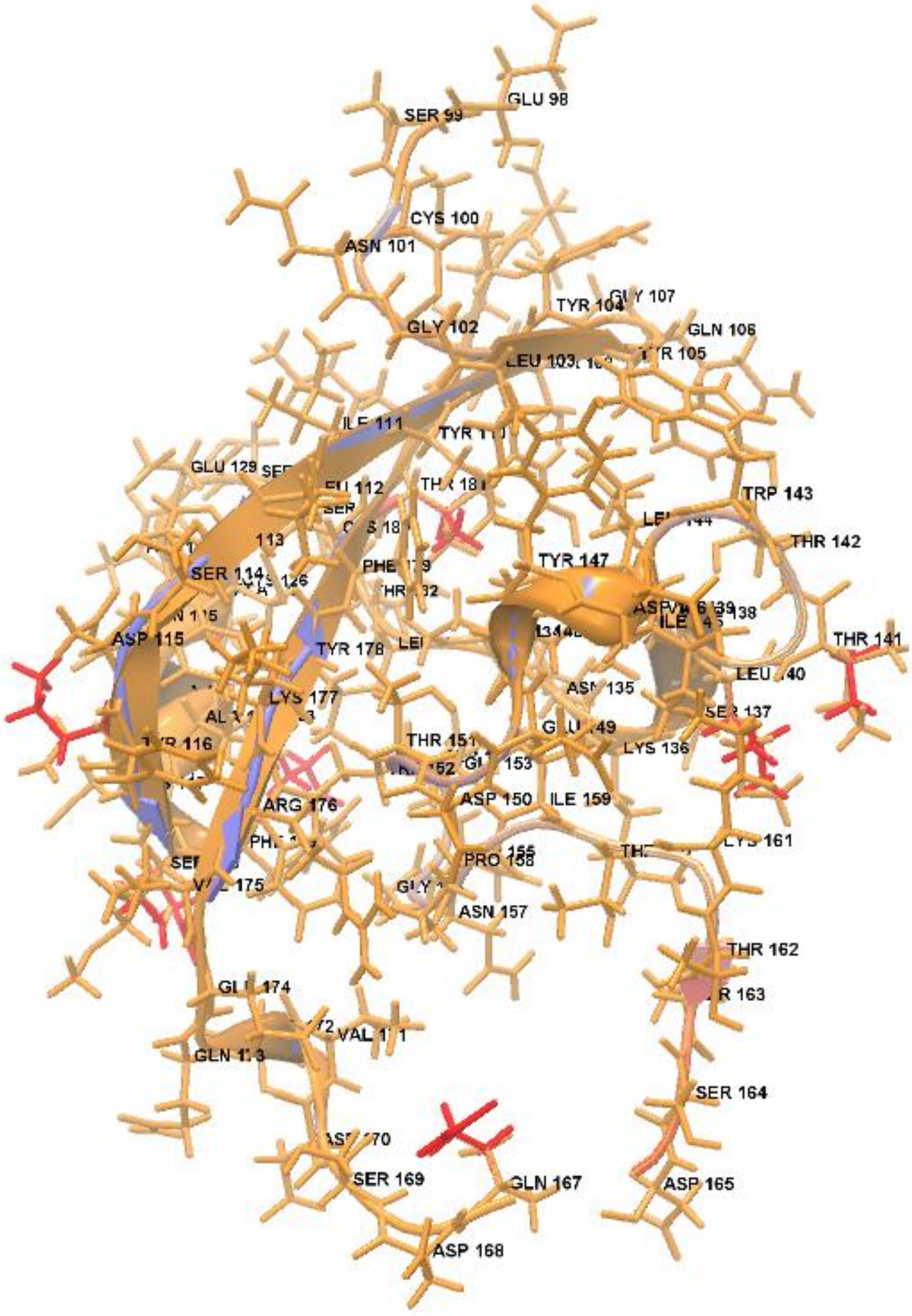

F MPXV Clade 1, Group IV, Protein model B6 (NC_003310)

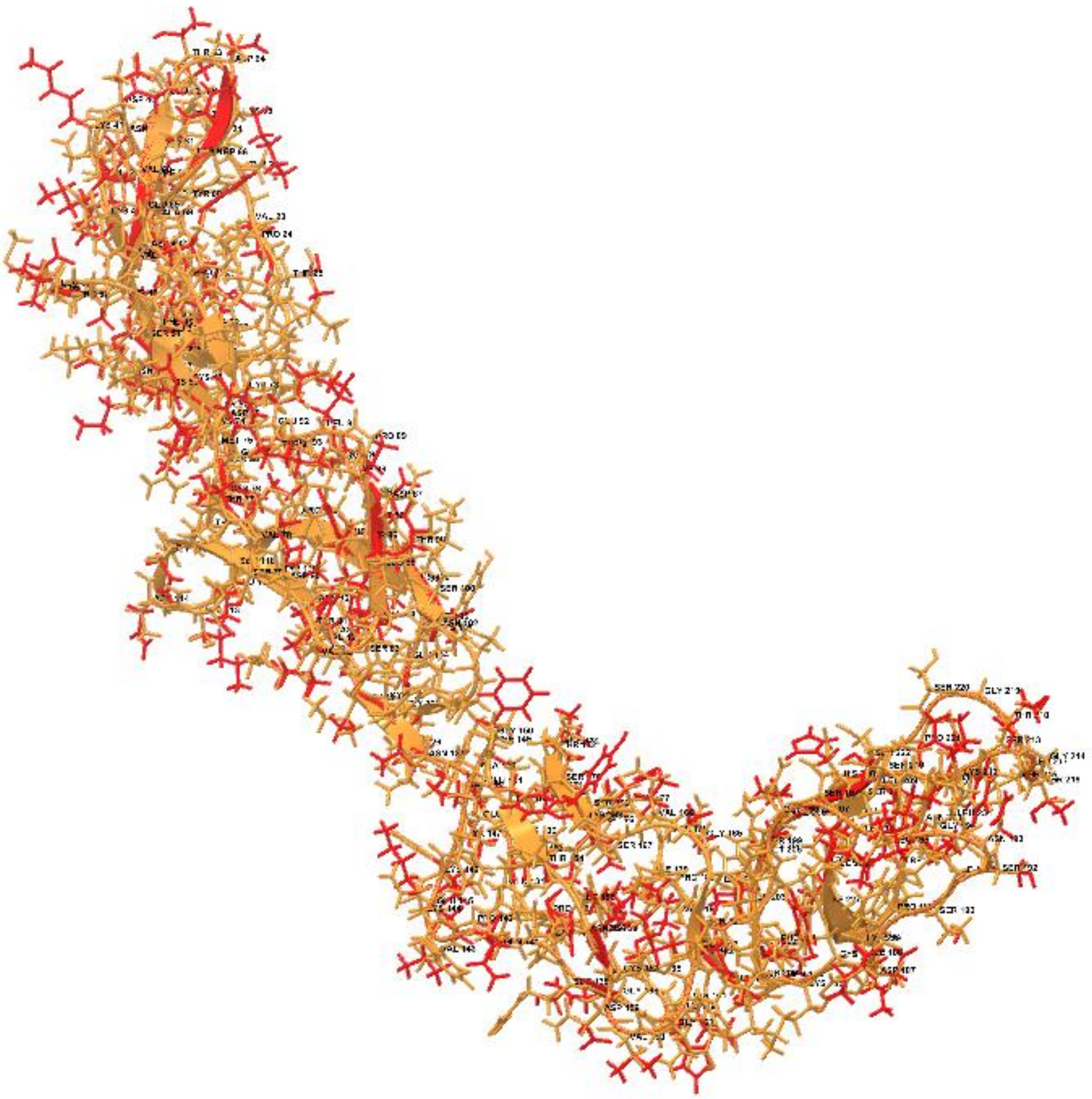

Supplementary Figure S5. MPXV antigen models (enlarged, labeled, stick and backbone structures) of Figure 6A for enhanced visualization. Refer to the Figure 6 legend for details.
