# Supplementary material for: Genomic and Antigenic Differences Between Monkeypox Virus and Vaccinia Vaccines: Insights and Implications for Vaccinology
Source: Int J Mol Sci. 2025 Feb 8;26(4):1428. doi: 10.3390/ijms26041428 (PMC11855751; doi:10.3390/ijms26041428)
Supplement: Supplementary file 1 [file ijms-26-01428-s001.zip › Fig S7 MPXV VACV MVA-BN, LC16m8 align 02-07-25.pdf]

A MPXV Clade 1, Group IV, Protein A29 (NC_003310)

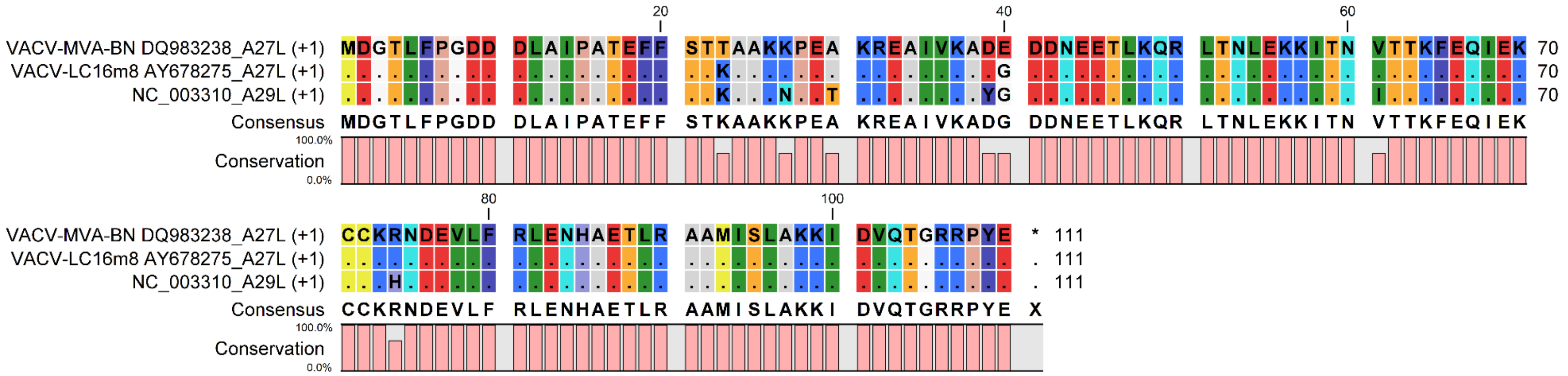

		1	2	3
VACV-MVA-BN DQ983238_A27L (+1)	1		98.20	93.69
VACV-LC16m8 AY678275_A27L (+1)	2	109		95.50
NC_003310_A29L (+1)	3	104	106	

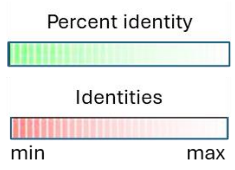

B MPXV Clade 1, Group IV, Protein A35 (NC_003310)

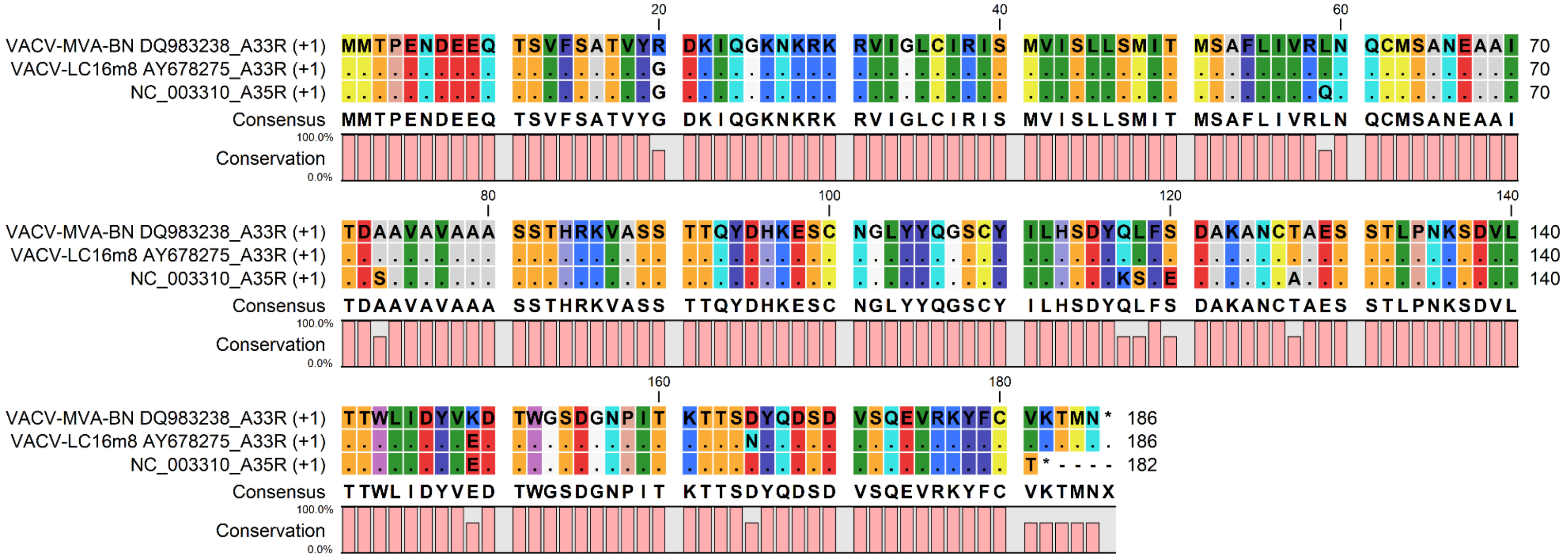

		1	2	3
VACV-MVA-BN DQ983238_A33R (+1)	1		98.39	93.01
VACV-LC16m8 AY678275_A33R (+1)	2	183		93.55
NC_003310_A35R (+1)	3	173	174	

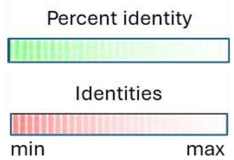

C MPXV Clade 1, Group IV, Protein B6 (NC_003310)

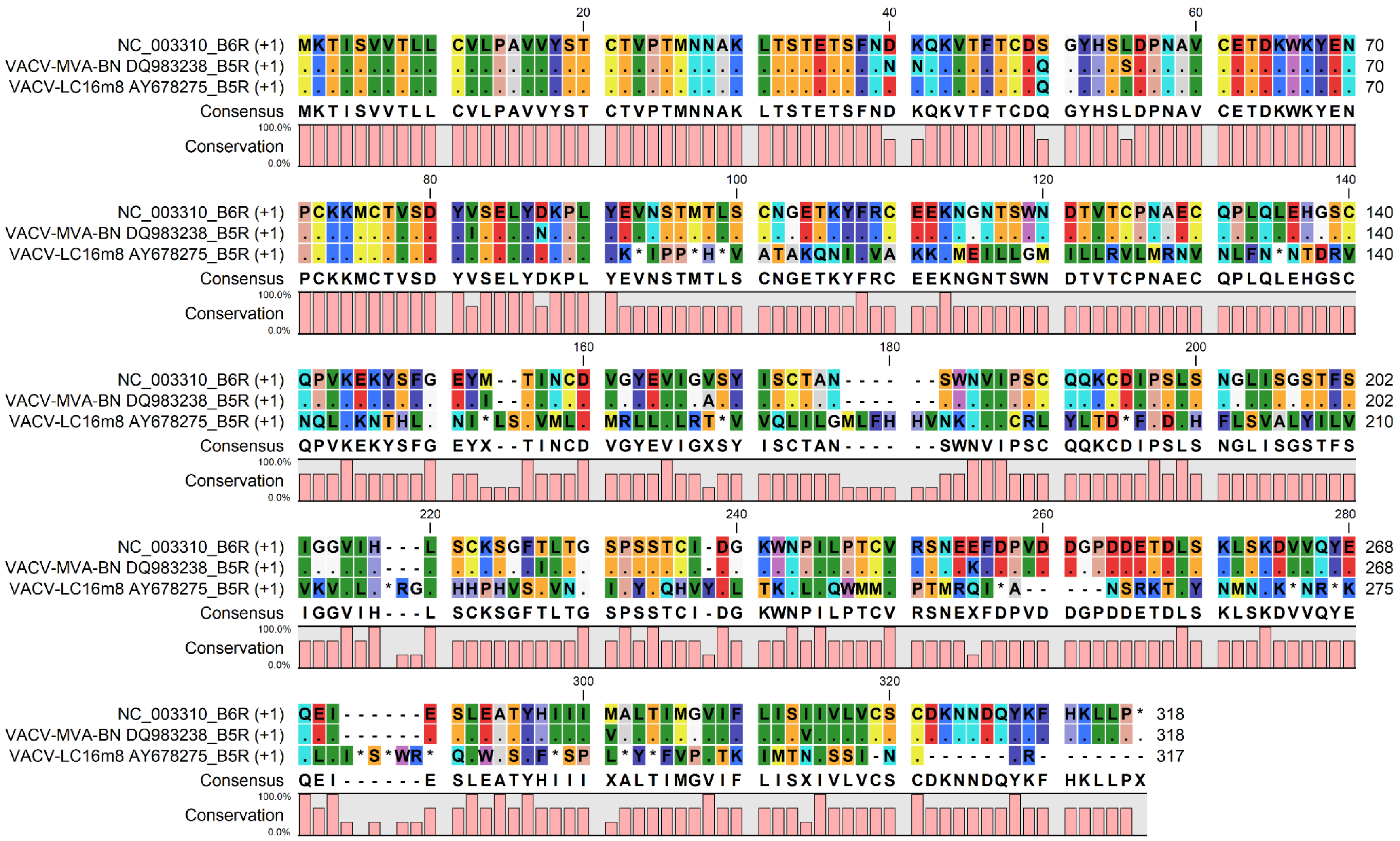

		1	2	3
NC_003310_B6R (+1)	1		96.23	41.07
VACV-MVA-BN DQ983238_B5R (+1)	2	306		39.58
VACV-LC16m8 AY678275_B5R (+1)	3	138	133	

Percent identity

Identities

min max

D MPXV Clade 1, Group IV, Protein E8 (NC_003310)

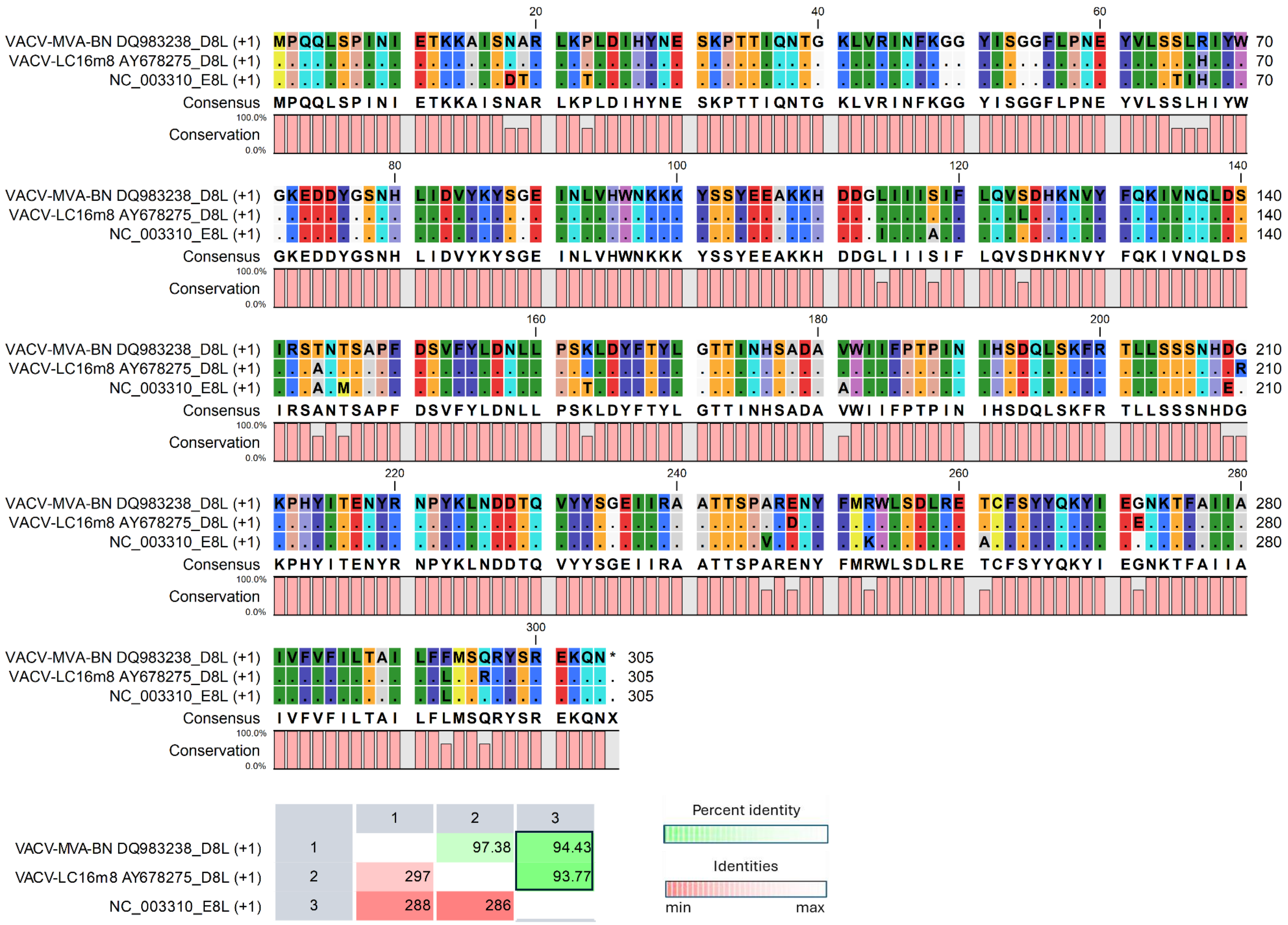

E MPXV Clade 1, Group IV, Protein H3 (NC_003310)

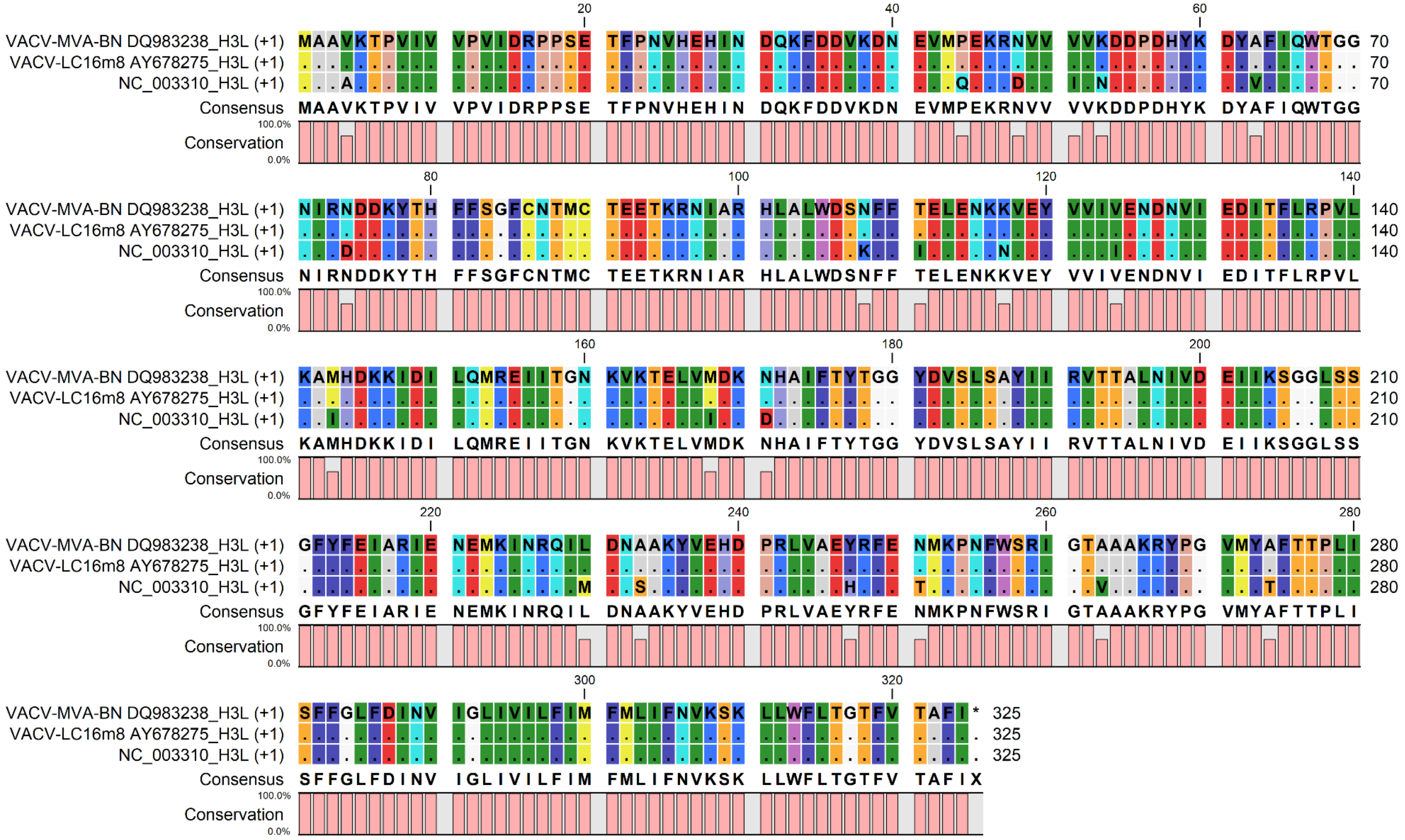

		1	2	3
VACV-MVA-BN DQ983238_H3L (+1)	1		100.00	93.85
VACV-LC16m8 AY678275_H3L (+1)	2	325		93.85
NC_003310_H3L (+1)	3	305	305	

Percent identity

Identities

min max

F MPXV Clade 1, Group IV, Protein M1 (NC_003310)

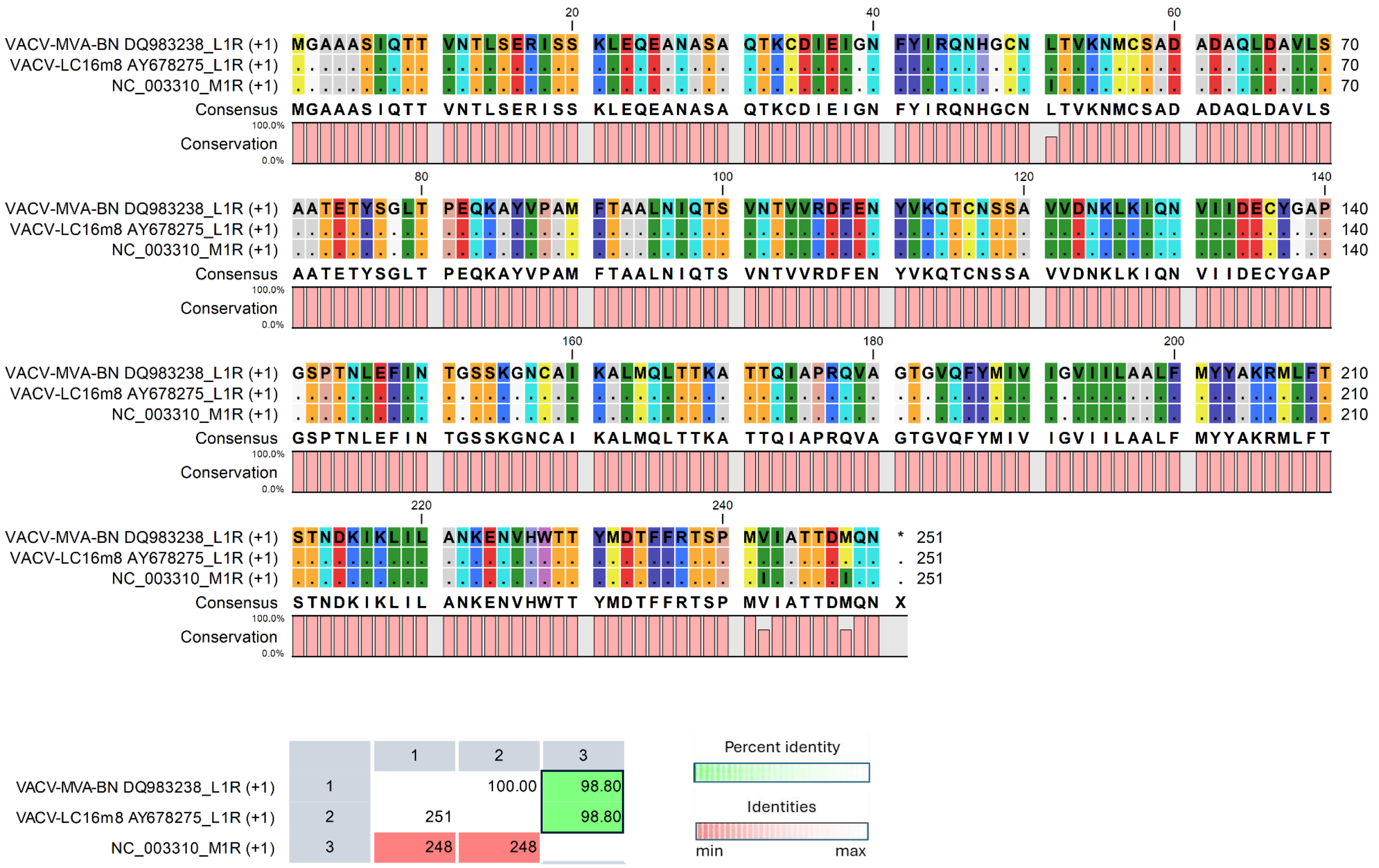

Supplementary Figure S7. Alignment and comparison of antigen sequences between MPXV and two current VACV vaccines. (**A-F)** This section presents the alignment of amino acid (AA) sequences for four MV membrane proteins (A29L, E8L, H3L, M1R) and two EV envelope proteins (A35L, B6R) from MPXV Clade 1 (NC_003310) and the VACV vaccines (MCV-BN and LC16m8). Matching residues are denoted by dots, while non-matching residues are explicitly labeled in the sequence. The pairwise comparison table below the alignment shows the calculated identities (the number of identical alignment positions between two sequences) and the percent identity (the proportion of identical residues in alignment positions between two sequences) highlighted by a box. The percent identities for A29, A35, E8, and H3 between MPXV (NC003310) and the VACV vaccines (MCV-BN and LC16m8) were notably high, ranging from 93% to 95%, with M1 being the highest at 98.8% (panel **F**). In contrast, the percent identity for B6 (panel **C**) between MPXV (NC003310) and the VACV vaccines (MCV-BN and LC16m8) showed a significant difference, at 96% and 41%, respectively. The symbol (*) represents an ambiguous residue in the AA sequence and (X) in the consensus sequence.
