# Supplementary material for: Genomic and Antigenic Differences Between Monkeypox Virus and Vaccinia Vaccines: Insights and Implications for Vaccinology
Source: Int J Mol Sci. 2025 Feb 8;26(4):1428. doi: 10.3390/ijms26041428 (PMC11855751; doi:10.3390/ijms26041428)
Supplement: Supplementary file 1 [file ijms-26-01428-s001.zip › Fig S8 MPXV antigens and AA sequences 02-07-25.pdf]

A MPXV Clade 1, Group IV, Protein A29 (NC_003310)

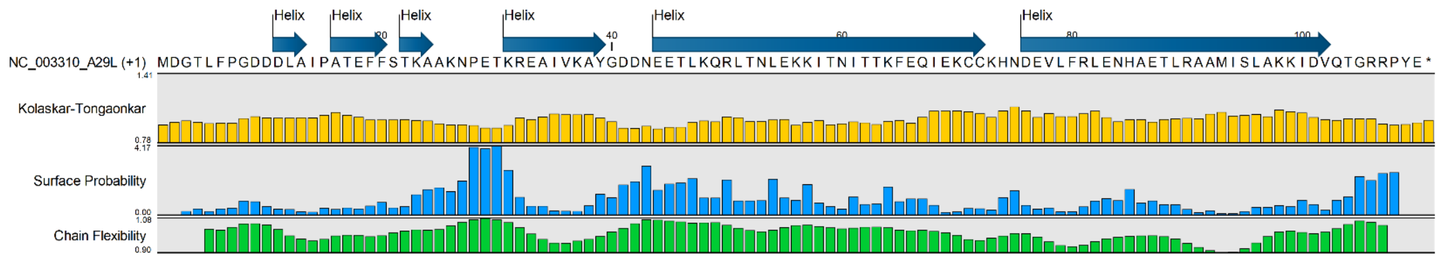

B MPXV Clade 1, Group IV, Protein A33 (NC_003310)

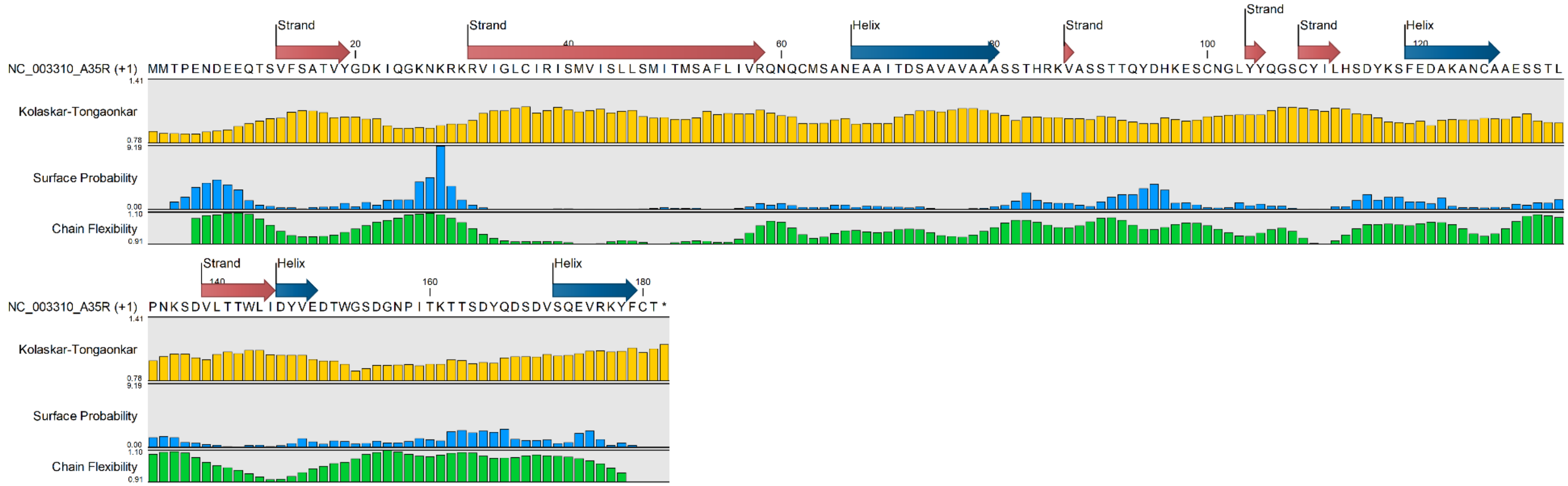

C

MPXV Clade 1, Group IV, Protein B6 (NC_003310)

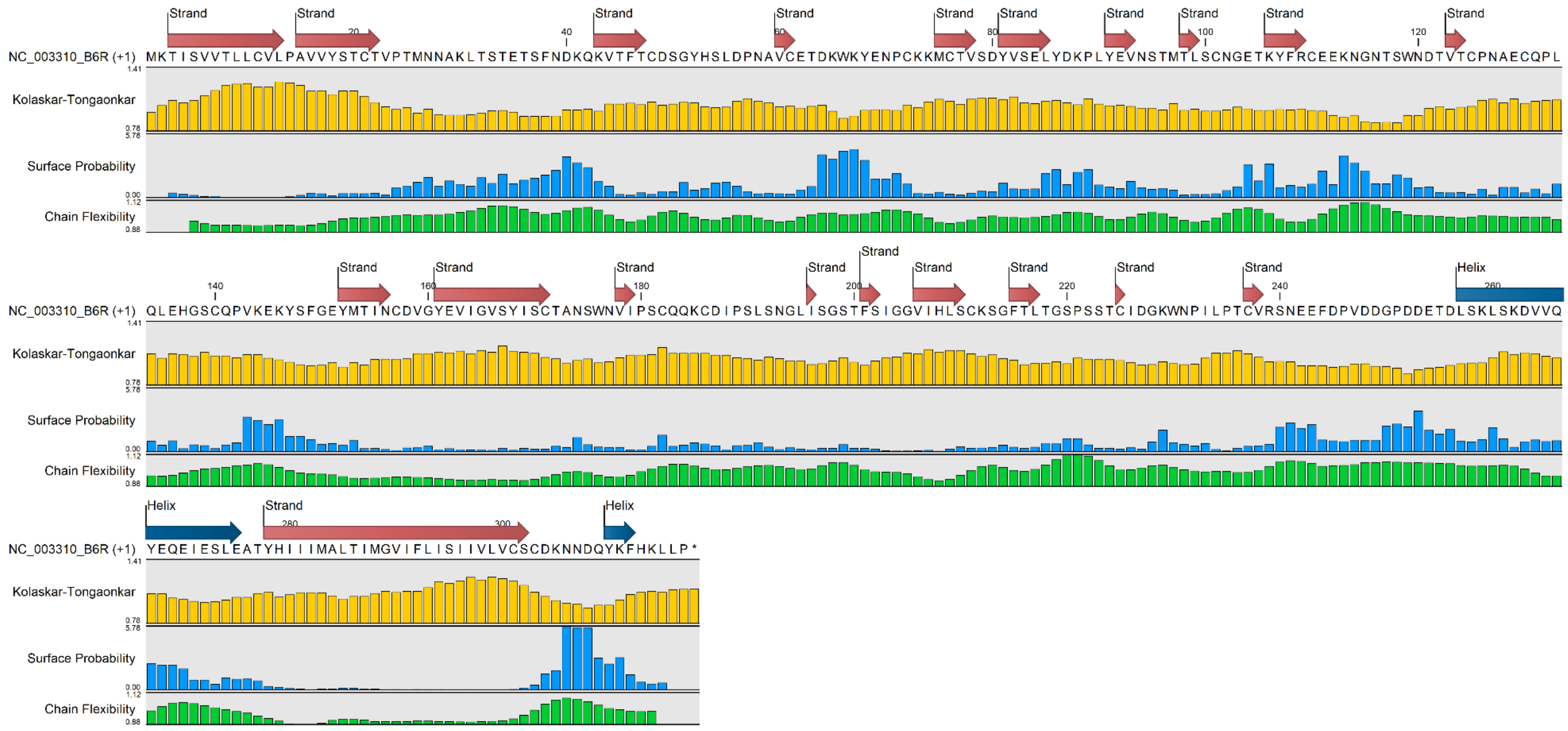

D

MPXV Clade 1, Group IV, Protein E8 (NC_003310)

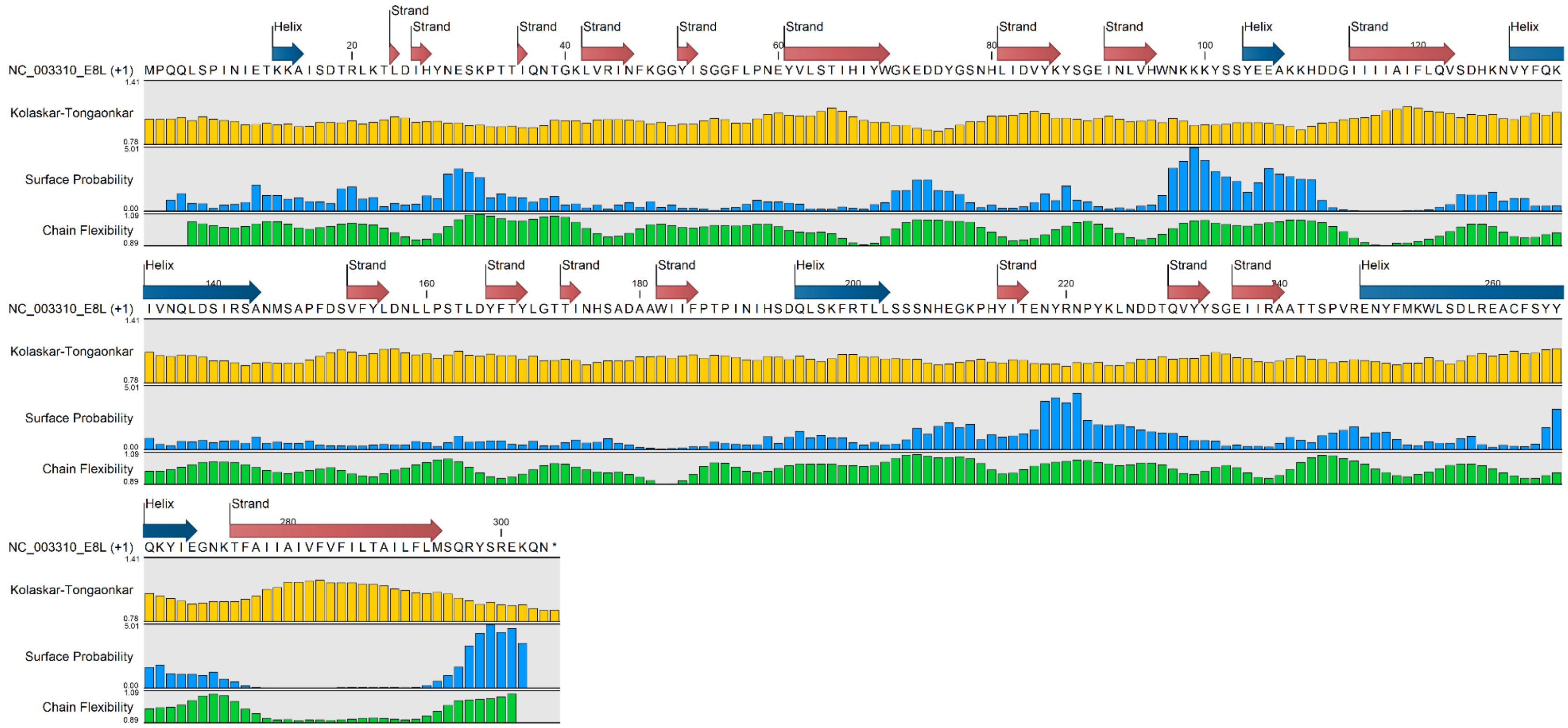

E

MPXV Clade 1, Group IV, Protein H3 (NC_003310)

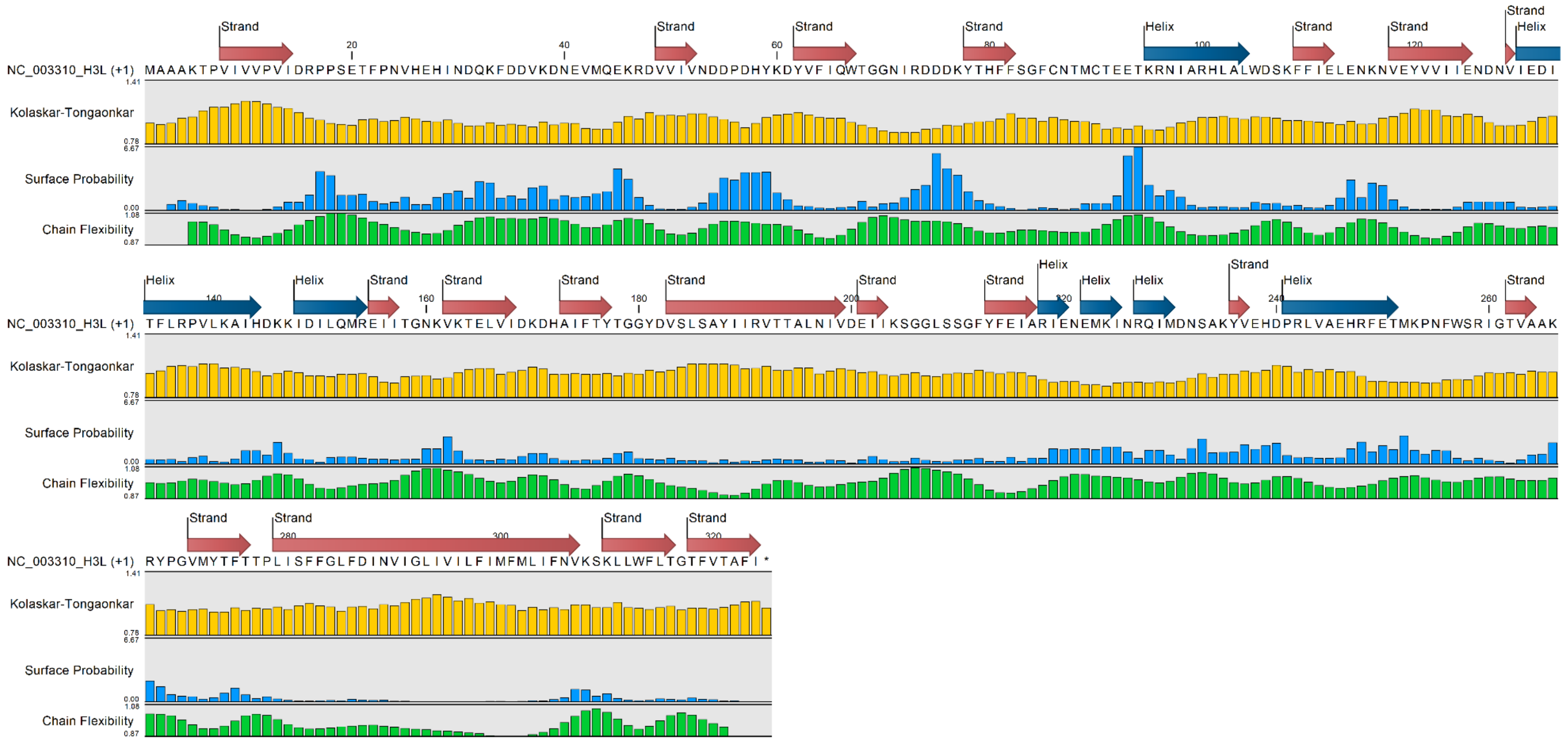

F MPXV Clade 1, Group IV, Protein M1 (NC_003310)

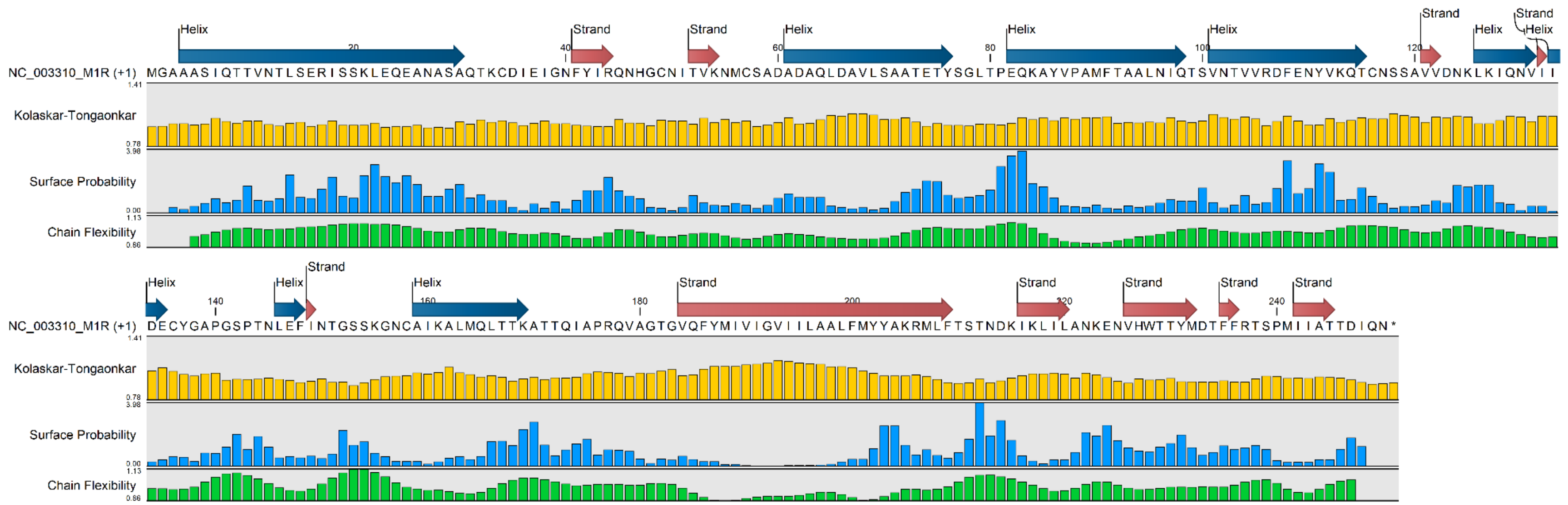

Supplementary Figure S9. Predicted protein properties of six neutralization determinants of MPXV Clade 1, Group IV (NC_003310). (**A-F)** Amino acid sequences of four MV membrane proteins (A29, E8, H3, M1) and two EV envelope proteins (A35, B6). The predicted structure (strand or helix) is shown above the amino acid sequence. The Kolaskar-Tongaonkar, Surface Probability and Chain Flexibility tracks (colored) are predictions of antigenic regions based on 1) hydrophilicity, surface accessibility and flexibility, 2) surface probability, and 3) backbone chain flexibility, respectively [47-50]. Generally, increased surface accessibility and chain flexibility correlate with antigenicity. A surface residue is one having more than 20 Å (2.0 nm) of water-accessible surface area as characterized by Emini et al [49]. The surface probability (S) at a specific sequence position (n) for a random hexapeptide sequence is set at 1.0. Probabilities > 1.0 suggest a higher likelihood of the residue being on the surface. For protein chain flexibility, B-Factors (Temperature Factors) are indicative of atomic displacement or flexibility within the protein structure. Higher B-factors (> 1.0) suggest greater flexibility [50]. The Kolaskar-Tongaonkar composite measure defines potential antigenic residues as having an average antigenic propensity (A_P) value ≥ 1.0 for 7 consecutive residues [48]. The asterisk (*) indicates a stop codon in the AA sequence.
