# Supplementary material for: Genomic and Antigenic Differences Between Monkeypox Virus and Vaccinia Vaccines: Insights and Implications for Vaccinology
Source: Int J Mol Sci. 2025 Feb 8;26(4):1428. doi: 10.3390/ijms26041428 (PMC11855751; doi:10.3390/ijms26041428)
Supplement: Supplementary file 1 [file ijms-26-01428-s001.zip › Fig S9 Bioinformatics Methods.pdf]

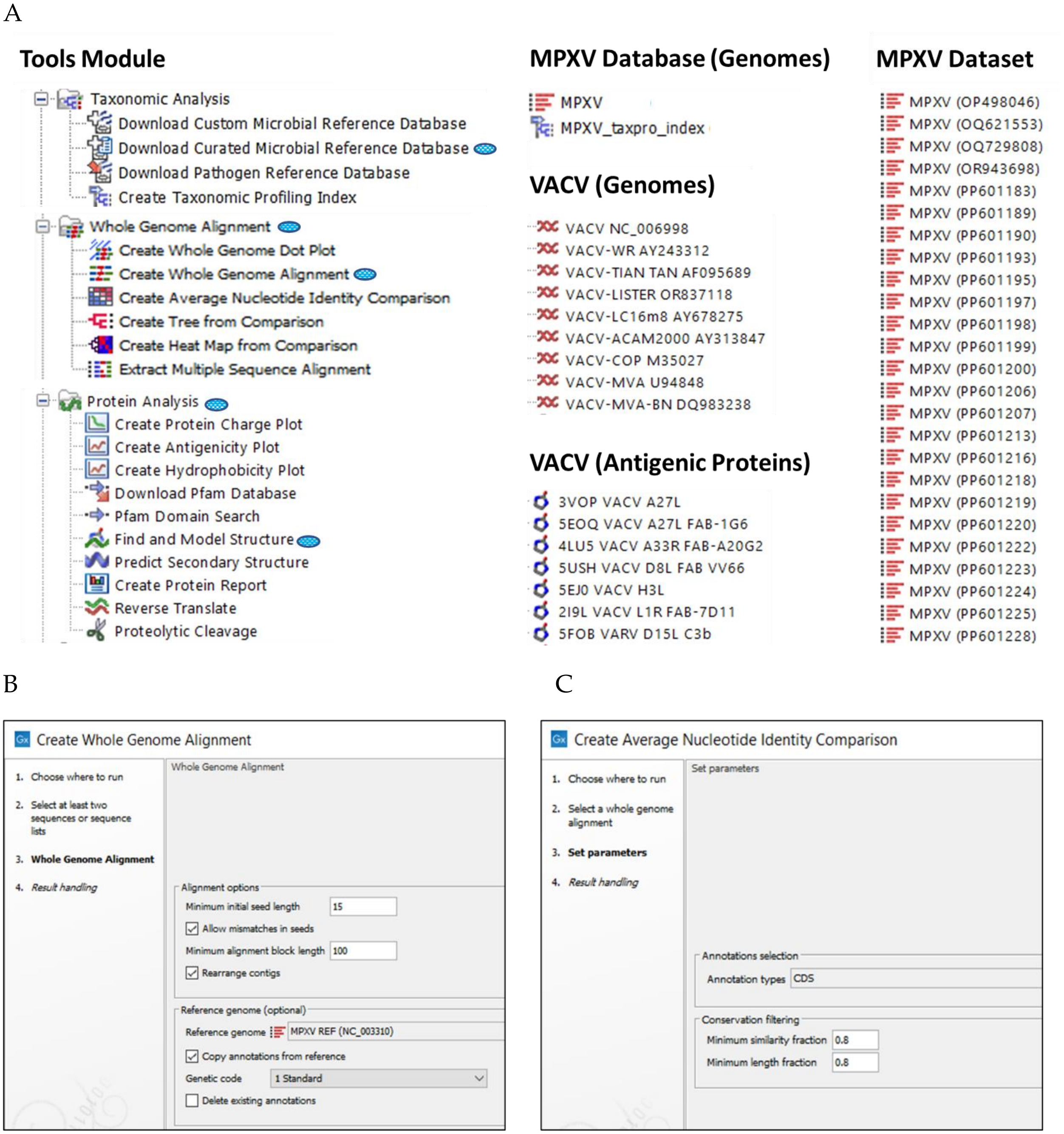

Supplementary Figure S9. Bioinformatics methods (**A**) CLC Microbial Genomics Module, database and datasets used for Monkeypox Virus (MPXV), Vaccinia Virus (VACV) Whole Genome Alignment (WGA) and protein analysis. Primary workflows and tools used for this study are designated by the blue virus icon. A MPXV dataset of 25 new Clade I genomes is shown; (**B**) WGA workflow steps (1-4) with parameter settings for WGA (bold) and annotation, e.g., MPXV REF genome; (**C**) The pairwise comparison workflow uses the WGA file as input to measure genome similarity and produces a matrix as output.
