# Supplementary material for: Genomic and Antigenic Differences Between Monkeypox Virus and Vaccinia Vaccines: Insights and Implications for Vaccinology
Source: Int J Mol Sci. 2025 Feb 8;26(4):1428. doi: 10.3390/ijms26041428 (PMC11855751; doi:10.3390/ijms26041428)
Supplement: Supplementary file 1 [file ijms-26-01428-s001.zip › Table S4 MPXV PP601216 Find and Model Structures.pdf]

Table S4. Summary of hits and structures found for MPXV (PP601216) antigenic proteins

MPXV virion	MPXV protein	Structures found (n)	Available structures	Chain	Rank	E-value	% Match identity	% Coverage
MV	A29	1	**3VOP**	C	1	**3.13752E-20**	**90.69767442**	**38.73873874**
MV	E8	1309	4E9O	X	1	1.2234E-158	93.13304721	75.40983607
			4ETQ	X	2	2.5556E-156	91.4893617	74.75409836
			**5USH**	X	3	**1.7373E-158**	**92.73504274**	**75.40983607**
			4ETQ	C	4	2.8765E-153	90.98712446	73.7704918
			5USH	A	5	1.201E-152	90.94827586	73.1147541
			5USL	X	6	1.5539E-155	92.24137931	74.42622951
			6B9J	X	7	2.0503E-155	91.45299145	74.42622951
			5USL	A	8	9.871E-155	91.81034483	74.09836066
			6KLZ	A	9	2.22323E-36	37.33333333	69.83606557
			6KM2	A	10	2.22323E-36	37.33333333	69.83606557
MV	H3	1	**5EJ0**	A	1	**1.6013E-142**	**84.38818565**	**65.23076923**
MV	M1	6	1YPY	A	1	1.093E-131	98.9010989	72.50996016
			1YPY	B	2	3.0146E-121	99.4047619	66.93227092
			**2I9L**	I	3	**4.5356E-125**	**99.42196532**	**68.92430279**
			4U6H	J	4	2.2423E-121	99.4047619	66.93227092
			4U6H	E	5	5.7892E-120	99.39759036	66.13545817
			6CJ6	B	6	1.67502E-08	26.31578947	57.37051793
EV	A35	7	4M1G	A	1	2.51943E-48	87.80487805	43.40659341
			3K7B	B	2	3.55025E-50	88.23529412	45.05494505
			3K7B	A	3	3.96318E-48	87.95180723	43.95604396
			4M1G	B	4	1.08783E-44	82.92682927	41.20879121
			**4LU5**	A	5	**1.23916E-50**	**90.36144578**	**45.05494505**
			4LU5	B	6	8.7988E-50	89.15662651	44.50549451
			4LQF	A	7	9.42671E-44	81.70731707	40.65934066
EV	B6	36	**5FOB**	C	1	**1.00571E-11**	**29.58333333**	**67.9245283**
			6V06	A	2	1.35695E-07	33.05785124	34.90566038
			6XSD	A	3	1.35695E-07	33.05785124	34.90566038
			6V08	A	4	1.46339E-07	33.05785124	34.90566038
			2UWN	A	5	5.34893E-08	28.05755396	39.93710692
			7JIK	A	6	1.39262E-07	33.05785124	34.90566038
			1QUB	A	7	1.36882E-07	33.05785124	34.90566038
			2V8E	A	8	1.47132E-07	28.05755396	39.93710692
			1C1Z	A	9	1.33245E-07	33.05785124	34.90566038
			4AYI	E	10	2.204E-07	31.95876289	28.30188679

Table S4. Summary of hits and structures found for MPXV (PP601216) antigenic proteins

Description
**STRUCTURE OF VACCINIA VIRUS A27**
VACCINIA D8L ECTODOMAIN STRUCTURE
VACCINIA VIRUS D8L IMV ENVELOPE PROTEIN IN COMPLEX WITH FAB OF MURINE IGG2A LA5
**STRUCTURE OF VACCINIA VIRUS D8 PROTEIN BOUND TO HUMAN FAB VV66**
VACCINIA VIRUS D8L IMV ENVELOPE PROTEIN IN COMPLEX WITH FAB OF MURINE IGG2A LA5
STRUCTURE OF VACCINIA VIRUS D8 PROTEIN BOUND TO HUMAN FAB VV66
STRUCTURE OF VACCINIA VIRUS D8 PROTEIN BOUND TO HUMAN FAB VV304
STRUCTURE OF VACCINIA VIRUS D8 PROTEIN BOUND TO HUMAN FAB VV138
STRUCTURE OF VACCINIA VIRUS D8 PROTEIN BOUND TO HUMAN FAB VV304
HUMAN CARBONIC ANHYDRASE II V143I VARIANT 00 ATM CO2
HUMAN CARBONIC ANHYDRASE II V143I VARIANT 15 ATM CO2
**VACCINIA VIRUS H3 ENV PROT; TARGET OF NEUT AB, EXHIBITS GLYCOSYLTRANSFERASE FOLD & BINDS UDP-GLUCOSE**
CRYSTAL STRUCTURE OF VACCINIA VIRUS L1 PROTEIN
CRYSTAL STRUCTURE OF VACCINIA VIRUS L1 PROTEIN
**STRUCTURE OF FAB 7D11 FROM A NEUTRALIZING ANTIBODY AGAINST THE POXVIRUS L1 PROTEIN**
VACCINIA L1/M12B9-FAB COMPLEX
VACCINIA L1/M12B9-FAB COMPLEX
STRUCTURE OF THE POXVIRUS PROTEIN F9
STRUCTURE OF MURINE IGG2A A27D7-FAB IN COMPLEX WITH VACCINIA ANTIGEN A33R AT THE RESOLUTION OF 1.6 ANG.
THE STRUCTURE OF THE POXVIRUS A33 PROTEIN REVEALS A DIMER OF UNIQUE C- TYPE LECTIN-LIKE DOMAINS.
THE STRUCTURE OF THE POXVIRUS A33 PROTEIN REVEALS A DIMER OF UNIQUE C- TYPE LECTIN-LIKE DOMAINS.
STRUCTURE OF MURINE IGG2A A27D7-FAB IN COMPLEX WITH VACCINIA ANTIGEN A33R AT THE RESOLUTION OF 1.6 ANG.
**STRUCTURE OF MURINE IGG2A A20G2-FAB IN COMPLEX WITH VACCINIA ANTIGEN A33R AT RESOLUTION OF 2.9 ANG.**
STRUCTURE OF MURINE IGG2A A20G2-FAB IN COMPLEX WITH VACCINIA ANTIGEN A33R AT THE RESOLUTION OF 2.9 ANG.
STRUCTURE OF MURINE IGG2B A2C7-FAB IN COMPLEX WITH VACCINIA ANTIGEN A33R AT THE RESOLUTION OF 2.3 ANG.
**CRYSTAL STRUCTURE OF HUMAN COMPLEMENT C3B IN COMPLEX WITH SMALLPOX INHIBITOR OF COMPLEMENT (SPICE)**
CRYSTAL STRUCTURE OF BETA-2 GLYCOPROTEIN I PURIFIED FROM PLASMA (PB2GPI)
PATIENT-DERIVED B2GPI
CRYSTAL STRUCTURE OF HUMAN RECOMBINANT BETA-2 GLYCOPROTEIN I (HRB2GPI)
CRYSTAL STRUCTURE OF HUMAN COMPLEMENT FACTOR H, SCR DOMAINS 6-8 (H402 RISK VARIANT).
HUMAN RECOMBINANT BETA-2-GLYCOPROTEIN 1
CRYSTAL STRUCTURE OF THE GLYCOSYLATED FIVE-DOMAIN HUMAN BETA2- GLYCOPROTEIN I.
CRYSTAL STRUCTURE OF HUMAN COMPLEMENT FACTOR H, SCR DOMAINS 6-8 (H402 RISK VARIANT).
CRYSTAL STRUCTURE OF HUMAN BETA-2-GLYCOPROTEIN-I (APOLIPOPROTEIN-H)
STRUCTURE OF A COMPLEX BETWEEN CCPS 6 AND 7 OF HUMAN COMPLEMENT FACTOR H.
