# Supplementary figures and images for: Genomic and Antigenic Differences Between Monkeypox Virus and Vaccinia Vaccines: Insights and Implications for Vaccinology
Source: Int J Mol Sci. 2025 Feb 8;26(4):1428. doi: 10.3390/ijms26041428 (PMC11855751; doi:10.3390/ijms26041428)

A MPXV Clade 1, Group IV, Protein A29 (NC\_003310)

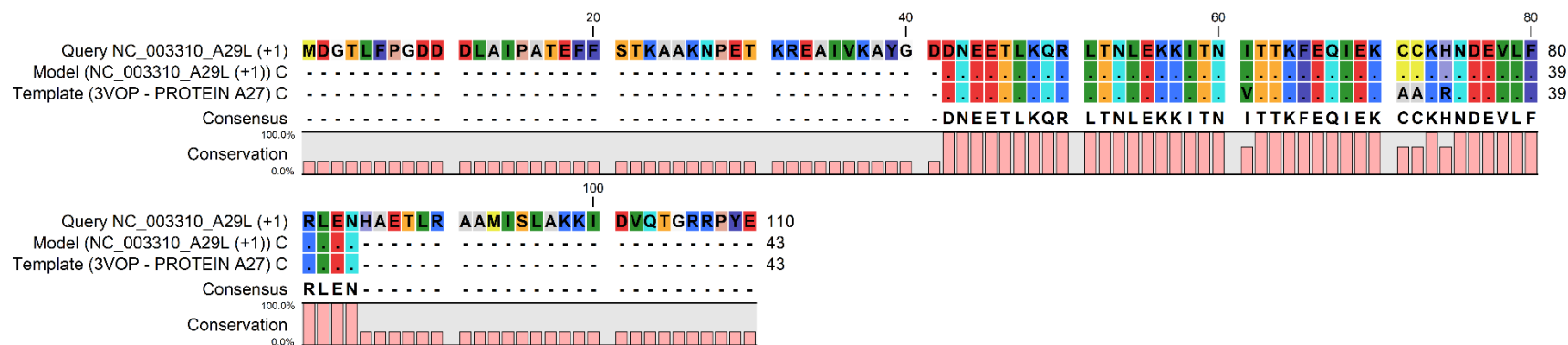

B MPXV Clade 1, Group IV, Protein A35 (NC\_003310)

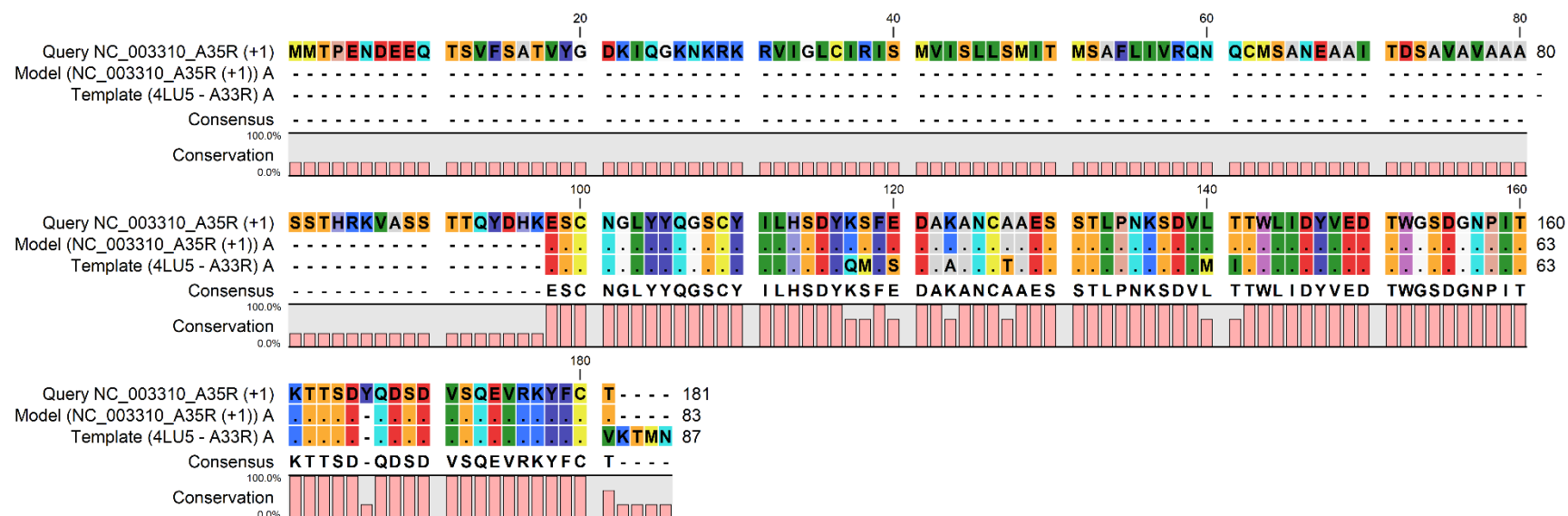

## C

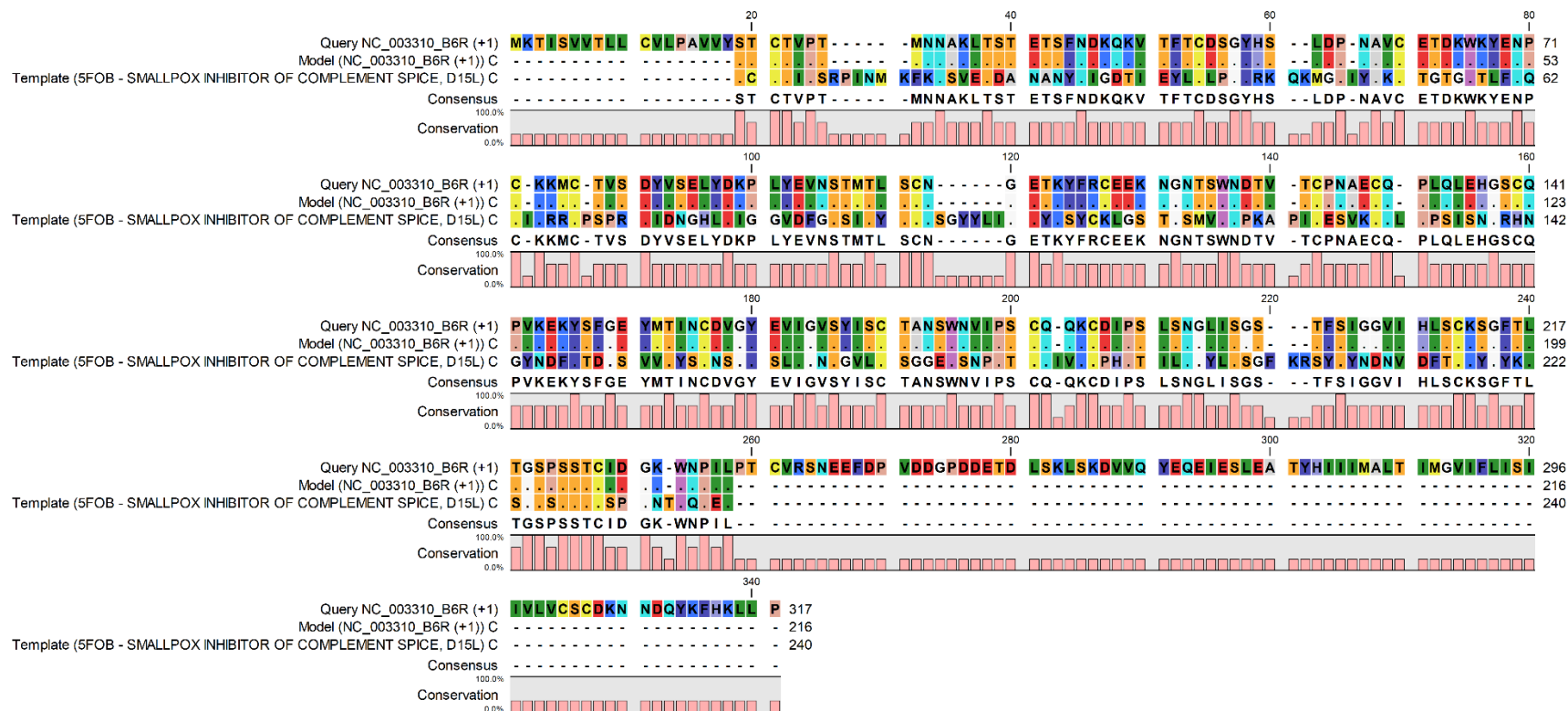

Supplement: Supplementary file 1 [file ijms-26-01428-s001.zip › Fig S6 MPXV model_VACV template align.pdf]
